# Qualitative study exploring the feasibility, usability and acceptability of neonatal continuous monitoring technologies at a public tertiary hospital in Nairobi, Kenya

**DOI:** 10.1136/bmjopen-2021-053486

**Published:** 2022-01-11

**Authors:** Mai-Lei Woo Kinshella, Violet Naanyu, Dorothy Chomba, Mary Waiyego, Jessica Rigg, Jesse Coleman, Bella Hwang, J Mark Ansermino, William M Macharia, Amy Sarah Ginsburg

**Affiliations:** 1 Department of Obstetrics and Gynecology, British Columbia Children’s and Women’s Hospital and The University of British Columbia, Vancouver, British Columbia, Canada; 2 Centre for International Child Health, BC Children's Hospital, Vancouver, British Columbia, Canada; 3 School of Arts and Sciences, Moi University, Eldoret, Uasin Gishu County, Kenya; 4 Department of Pediatrics, The Aga Khan University - Kenya, Nairobi, Kenya; 5 Department of Pediatrics, Pumwani Maternity Hospital, Nairobi, Kenya; 6 Department of Anesthesiology, The University of British Columbia, Vancouver, British Columbia, Canada; 7 Evaluation of Technologies for Neonates in Africa, Seattle, Washington, USA; 8 Clinical Trial Center, University of Washington, Seattle, Washington, USA

**Keywords:** continuous monitoring technologies, neonate, Africa, medical technology design, user perspectives, qualitative research

## Abstract

**Objective:**

To assess the feasibility, usability and acceptability of two non-invasive, multiparameter, continuous physiological monitoring (MCPM) technologies for use in neonates within a resource-constrained healthcare setting in sub-Saharan Africa.

**Design:**

A qualitative study using in-depth interviews and direct observations to describe healthcare professional and caregiver perspectives and experiences with investigational MCPM technologies from EarlySense and Sibel compared with selected reference technologies.

**Setting:**

Pumwani Maternity Hospital is a public, high-volume, tertiary hospital in Nairobi, Kenya.

**Participants:**

In-depth interviews were conducted with five healthcare administrators, 12 healthcare providers and 10 caregivers. Direct observations were made of healthcare providers using the technologies on 12 neonates overall.

**Results:**

Design factors like non-invasiveness, portability, ease-of-use and ability to measure multiple vital signs concurrently emerged as key themes supporting the usability and acceptability of the investigational technologies. However, respondents also reported feasibility challenges to implementation, including overcrowding in the neonatal unit, lack of reliable access to electricity and computers, and concerns about cost and maintenance needs. To improve acceptability, respondents highlighted the need for adequate staffing to appropriately engage caregivers and dispel misconceptions about the technologies.

**Conclusion:**

Study participants were positive about the usefulness of the investigational technologies to strengthen clinical care quality and identification of at-risk neonates for better access to timely interventions. These technologies have the potential to improve equity of access to appropriate healthcare services and neonatal outcomes in sub-Saharan African healthcare facilities. However, health system strengthening is also critical to support sustainable uptake of technologies into routine care.

**Trial registration number:**

NCT03920761.

**Strengths and limitations of this study**

- We interviewed healthcare administrators, providers and caregivers to understand the feasibility, usability and acceptability of investigational technologies from multiple perspectives.
- The purposeful sampling design elicited a wide range of perspectives although these cannot be used to determine representative frequency of themes.
- The triangulation of direct observations with in-depth interviews helped to strengthen reliability of findings.
- The current study is compared with findings from a previous study conducted at a private healthcare facility in Nairobi, Kenya with the same technologies and methodology to illuminate different implementation factors between private and public tertiary hospitals.

## Background

Leading causes of neonatal deaths, including 35% due to preterm birth complications, 24% due to birth asphyxia and trauma, and 15% due to neonatal sepsis and infections, are preventable with quality facility-based care.^1 2^ However, effective implementation of evidence-based neonatal interventions may require monitoring of vital signs and time-sensitive clinical follow-up, which may be compromised in resource-constrained healthcare settings.^3 4^ Locally appropriate technologies to support early detection of physiologically unstable neonates requiring timely intervention have the potential to improve quality of care and neonatal health outcomes.^5^

The Evaluation of Technologies for Neonates in Africa (ETNA) platform aims to boost development and optimisation of promising neonatal medical technologies to be used in resource-constrained healthcare facilities. Understanding user perspectives in the intended setting is critical to medical technology design, development, deployment and eventual uptake and acceptance. However, the feasibility, appropriateness and acceptability of novel technologies for improving maternal and neonatal health are not often adequately investigated, thereby compromising implementation efforts.^6^ The ETNA platform previously conducted a qualitative evaluation of two novel, non-invasive, multiparameter, continuous physiological monitoring (MCPM) technologies developed by EarlySense and Sibel at Aga Khan University Hospital (AKUH), a private, tertiary hospital in Nairobi, Kenya where MCPM technologies were already used in neonatal intensive care (Ginsburg *et al* 2021). By contrast, Pumwani Maternity Hospital (PMH) is a public, high-volume maternity hospital in Nairobi where MCPM technologies are not routinely used. In the current study, we assessed the feasibility, usability and acceptability of the same MCPM technologies at PMH to better understand the technologies’ use for neonates within a resource-constrained healthcare setting in sub-Saharan Africa.

## Methods

### Study design and setting

**Figure 1:**
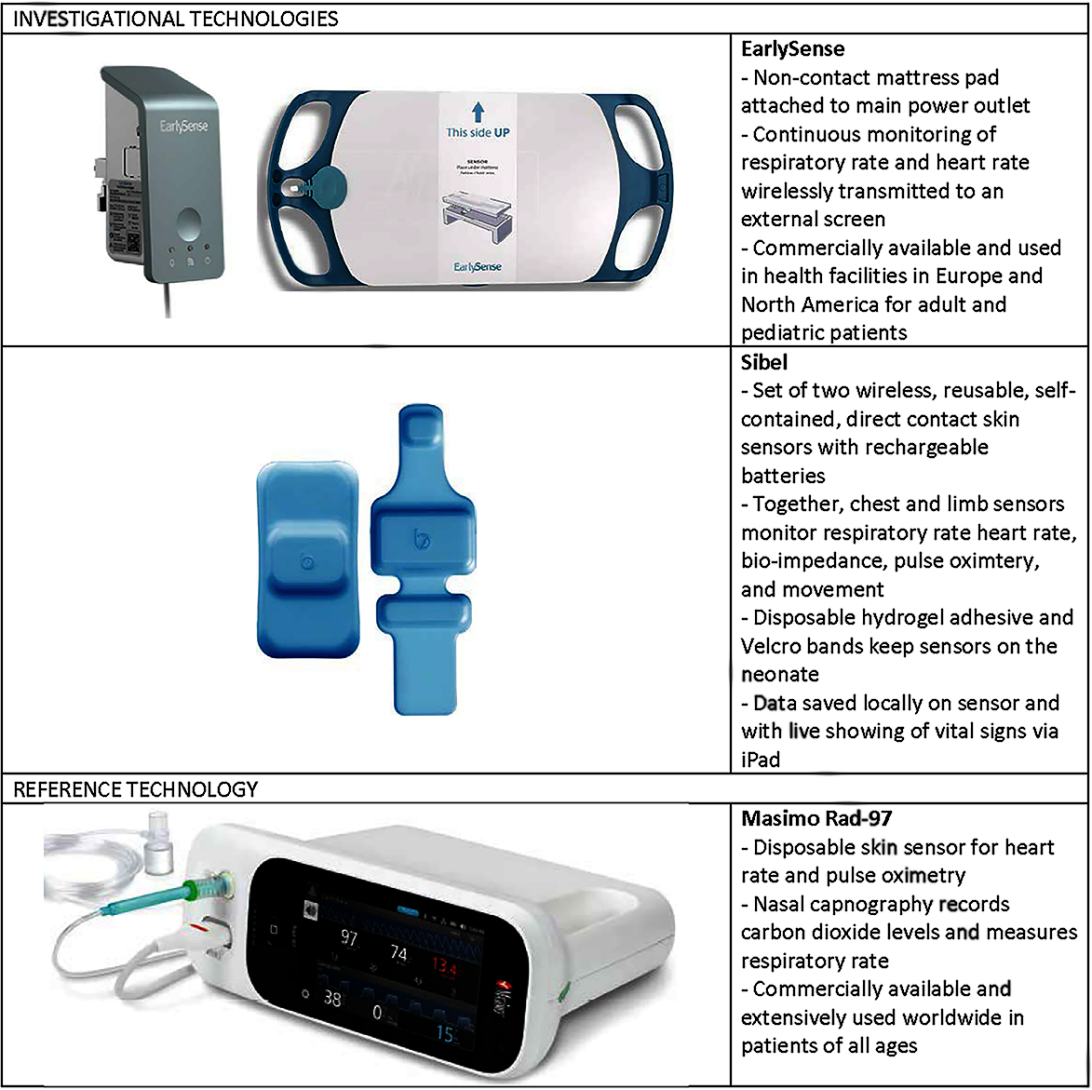
Overview of the three multiparameter continuous physiological monitoring technologies.

Comprised of in-depth interviews (IDIs) and direct observations, this descriptive qualitative study elicited perspectives and experiences of healthcare professionals and caregivers around MCPM technology feasibility, usability and acceptability. We evaluated the accuracy, reliability and performance of novel MCPM technologies in comparison with verified reference technologies (figure 1). Frequently used in hospitals worldwide, the Masimo Rad-97 reference technology was selected based on its capability for high-resolution data collection and neonatal capnometry and pulse oximetry. We present the findings based on the ‘Consolidated criteria for Reporting Qualitative research’.^7 8^ The current study used the following definitions^9 10^:

- Feasibility involved systemic factors required for implementation of MCPM technologies, such as hospital infrastructure, operational capacities and functional capacities of available healthcare providers (HCP).
- Usability involved design factors that influenced HCP user experience, such as ease and efficiency of use, frequency of errors, memorability to a casual user and user satisfaction.
- Acceptability involved factors that influenced the willingness of healthcare administrators (HCA), HCP and caregivers to use the technology.

PMH is a public, tertiary referral hospital serving Nairobi, Kenya and is the largest referral maternity hospital in sub-Saharan Africa with an average of 50–100 deliveries a day. Neonates in good health accompany their mothers to the postnatal ward while neonates with health complications are admitted to the neonatal unit, a large hall separated into 11 cubicles representing different diagnoses and care requirements. Neonates in more critical health conditions are placed in cubicles closest to the nursing station, while stable neonates awaiting discharge are moved to cubicles on the other side of the hall. Neonates commonly share cots and incubators with up to four neonates in each. The neonatal unit is typically staffed by three nurses and three clinical officers or physicians during the morning shift, and then two nurses and one clinical officer or physician during the afternoon and night shifts. The study moved between the different cubicles within the neonatal unit and employed two dedicated study nurses to support the study. Caregiver visitation times are restricted to every 3 hours for the mothers to breastfeed and care for the neonates.

### Recruitment and data collection

A purposefully drawn study sample included HCA, direct and indirect HCP and caregivers of neonates enrolled in ETNA. Direct HCP consisted of ETNA study nurses who were direct users of the MCPM technologies (HCP-D) and indirect HCP included hospital physicians, nurses and clinical staff involved in neonatal care but who did not actively use the investigational or reference MCPM technologies (HCP-I). Multiple MCPM technologies were used with each neonate enrolled in ETNA during their hospital stay. A sample size of five HCA, 12 HCP and 10 caregivers was estimated to reach data saturation covering a wide range of perspectives from the healthcare staffing positions and caregivers available.

Study recruitment was publicised using flyers and potential participants were approached in person by a member of the qualitative study team, who introduced themselves and the ETNA study. To minimise bias, a Kenyan research consultant (VN, PhD in sociology, woman) and two trained female research assistants (diplomas in health sciences) who did not know participants prior to the study activities were hired to conduct the IDIs with the enrolled qualitative study participants and the direct observations of the ETNA study nurses.

IDIs with HCA, HCP and caregivers and direct observations of HCP-D were conducted between 23 November and 1 December 2020 following semistructured IDI and structured observation guides. To investigate the accuracy, reliability and performance of the technologies, IDIs included questions regarding reactions to technology use, consideration of result trustworthiness, advantages and concerns about each technology, local health system constraints and suitability within their facility (online supplemental file 1). While the focus of the study is to understand the feasibility, usability and acceptability of the investigational technologies, the same questions were asked about all three technologies to allow for contextualisation and comparison. Additionally, direct observations of HCP-D using the technologies covered three different phases of usage for each of the MCPM technologies: (1) technology preparation and initial application; (2) ongoing technology monitoring and troubleshooting; and (3) technology disconnection, removal and cleaning (online supplemental file 2). Data collection guides were developed for the ETNA qualitative study and piloted by the Kenyan data collection team during training to refine questions. After obtaining written informed consent, IDIs were conducted in person in a quiet, private place within PMH in English or Kiswahili, the major local languages in Kenya, depending on study participant preference. IDIs took between 18 and 78 min to conduct with an average length of 46.6 min. Written informed consent was obtained from HCP-D for observations IDIs were audio-recorded with permission, field notes recorded during data collection, and no repeat IDIs were conducted.

10.1136/bmjopen-2021-053486.supp1 Supplementary data

10.1136/bmjopen-2021-053486.supp2 Supplementary data

### Data analysis

IDIs were transcribed verbatim and translated into English. Transcripts were uploaded into NVivo V.12 software (QSR International, Melbourne, Australia) for qualitative analysis following a thematic approach. Thematic analysis involved becoming familiar with the data, generating initial codes collating identified codes into themes, and describing themes using illustrative quotes.^11^ A coding framework (online supplemental file 3) was developed deductively from study objectives to cover feasibility, usability and acceptability as well as inductively from emergent themes by the ETNA study team (M-LWK, VN, DC, JR, JC, WM, ASG). VN conducted the primary coding with review by M-LWK.

10.1136/bmjopen-2021-053486.supp3 Supplementary data

Data confidentiality was ensured through limiting access of study materials to authorised personnel, deidentifying participants using codes, and aggregating demographic features.

### Patient and public involvement

Neither patients nor public were involved in the design or conduct of the study.

## Results

Direct observations of HCP-D using the technologies on 12 neonates were made and IDIs conducted with 27 participants, including 5 HCA, 10 HCP-I (6 nurses, 2 clinical officers and 2 physicians), 2 HCP-D (two study nurses,) and 10 caregivers. No potential participants declined to participate. Interviewed healthcare professionals were woman except for one male clinician, and ranged in age from 24 to 58 (average 36.2) years. With a median of 5 (range <1–35) years of work experience in the medical field, approximately half (8 of 17) of the healthcare professionals held diplomas or certificates as their highest level of formal education. Four healthcare professionals were pursuing a first degree or completed an undergraduate degree, while three held master degrees and two had medical degrees. Interviewed caregivers were woman ranging in age from 19 to 28 (average 22.3) years. A majority reported that this was their first child (6 of 10 caregivers, range 1–3 children). Eight caregivers had secondary-level education while two had primary-level education. Most (8 of 10) caregivers reported they were unemployed or a housewife, and two caregivers shared that they were involved in informal, small-scale business. Reported occupations of husbands and partners included mason, mechanic, electrician, watchman, businessman, marketing and driver.

Key themes reported regarding technology feasibility included the number of neonates needing monitoring, reliable access to electricity and computers, and cost and maintenance implications of the MCPM technologies. Ease and efficiency of use, non-invasiveness and portability were critical features highlighted for usability. Supporting improved monitoring capacities, concerns about radiation and electrical currents, and a need for caregiver engagement were central themes noted for the acceptability of the MCPM technologies.

### Feasibility

#### Numbers of neonates to monitor

A major challenge at PMH was overcrowding, resulting in the common practice of multiple neonates within a single cot. As a HCA shared, ‘*…*we are admitting so many babies but our capacity is low…the capacity of the unit is small as compared to the neonates we receive and that is why you find there are two-three-four babies in one-unit bed.’ (*HCA, 1*).

HCA and HCP posited that overcrowding impacted the feasibility of scaling up individual MCPM technologies for neonates, particularly the EarlySense technology which is placed under the mattress. A study nurse said, ‘We've not used [the EarlySense technology] where babies are sharing the baby cot. …we don't know of its efficiency when there’s more than one [baby]*…*’ *(HCP-D, 1*). A HCA said that because the EarlySense technology ‘can only take one [neonate], so it means for us we would have to prioritize really who we have to monitor so that we give them their space’ *(HCA, 3*). The EarlySense technology was designed for each neonate to be in an individual cot but healthcare professionals at PMH shared that this may reduce the number of neonates that could be admitted given the current practice of sharing cots.

The Sibel technology may better accommodate sharing cots as one HCA highlighted, ‘sharing incubators, [the Sibel technology] is comfortable to use. I like that it is compact*…*’ *(HCA, 4*). However, overcrowding still had implications for service delivery as different neonates would need to be carefully identified and their readings easily distinguishable from one another. As a clinical officer said, A ‘challenge would be telling specifically this is for this baby while you have 20 babies on this [Sibel technology]. They will need to be sure that this belongs to this baby in this room. They will need to have codes for the specific baby*…*’ *(HCP-I, 9*).

#### Reliable access to electricity and computers

While a back-up generator was available at PMH, HCA and HCP reported that the generators were not always functional and frequently required repairs. Electrical outages could lead to delays in using technologies that required uninterrupted electricity supply, ‘If there is power failure and a generator is faulty, we end up not doing what we need until electricity is back’ *(HCP-D, 2*).

Unreliable electricity had direct implications for the EarlySense technology, which was connected to wall power. As one nurse said, ‘I saw [the EarlySense technology] is using power. So, if possible, can we have the one without the power? So that if there is no electricity we can still use it’ *(HCP-I, 1*). The Sibel technology used a rechargeable battery, but HCP said that ensuring the technology was fully charged when needed and charging between electrical outages would be a challenge in a busy neonatal unit. For example, a nurse said, ‘*…* unlike other devices which you just connect to the (wall) socket and they are ready to use, [the Sibel technology has] to be prepared… So, charging them and making sure they are ready for use is a challenge for a big hospital like Pumwani’ *(HCP-D, 2*).

Additionally, both investigational technologies relied on the use of external screens and computers, which would require investments in equipment, spacing and electrical infrastructure, and training for staff to use along with the current manual documentation systems. As a nurse said, ‘There’s no regular access to computer. There’s only one, in in-charge office and… everything else is manual’ *(HCP-D, 1*).

#### Cost and maintenance

Cost and maintenance implications of the MCPM technologies were also highlighted by HCA and HCP as critical factors influencing the feasibility of potential scale-up. As a public hospital, HCA shared that PMH followed the government procurement process, and while there were a procurement and budget committee and a health management board at PMH that took into account what HCP needed in their department, the medical superintendent had to approve the purchase and the Kenya Medical Supplies Authority did most of the purchasing. Consequently, HCA said that a lack of funds at PMH to purchase equipment is a challenge. HCA shared that PMH was often reliant on donors and partners to fill in the gaps, ‘not having funds for the equipment is a big issue because money from the county or NMS (Nairobi Metropolitan Services) is not available to us, and we have to look for donors and partners who are able to procure the equipment for us’ *(HCA, 4*). In addition to the initial costs of purchasing the technology, there would be additional costs around maintenance. A HCA said, ‘…we have to think through how we are going to maintain this servicing. So there is a cost to it beyond the buying the purchase’ *(HCA, 3*). Some wondered if replacement parts and the training of local biomedical engineers to service and repair the EarlySense and Sibel technologies were available in the country. Taken together with funding challenges for their initial purchase, ongoing maintenance could limit sustainable scale-up into routine care as an ETNA study nurse observed, ‘I have seen sometimes maybe… because of poor maintenance…it’s not effective for as long as it should have been’ *(HCP-D, 1*).

### Usability and acceptability

Direct observations of HCP-D using the MCPM technologies within the PMH neonatal unit supported usability with appropriate availability of training and support. Similar to the Masimo reference technology, application of the EarlySense and Sibel technologies to a neonate each took on average 5 min and the HCP-D were observed to not face any difficulties with preparation, initial application, monitoring, disconnection or cleaning. No use errors where mistakes could potentially happen were observed with either investigational technology. There was one observation with each of the investigational technologies where a HCP-D required assistance from another study nurse to help calm an irritable neonate, which interfered with technology readings (EarlySense) or application (Sibel).

#### Ease and efficiency in use

Design factors shared by HCA and HCP that impacted user experience included that the MCPM technologies appeared easy to use and clean. Speaking of the EarlySense technology, a HCA said, ‘Looks easy to clean. That is a big issue for us because we need to observe high hygiene standards’ *(HCA, 4*). An ETNA study nurse who used the technologies noted, ‘What I liked about [the EarlySense technology] is that it’s easy to place. It’s quite straightforward…’ *(HCP-D, 1*). A HCA said, ‘[The Sibel technology] looks easy to use because you are just attaching to the extremity and the trunk’ *(HCA, 3*). The investigational technologies were described as easy to use for someone without extensive training.

Additionally, the MCPM technologies were described as being able to efficiently collect multiple vital signs within a single device. A clinical officer said of the EarlySense technology, ‘you will be able to collect most crucial data… So you get a lot of data using a short time period’ *(HCP-I, 4*). Of the Sibel technology, a nurse observed, ‘…It is taking four vitals at the same time, whereas if it is manual, I would have four gadgets…[such as] stethoscope, thermometers… Now that small gadget I just place it on the chest…it is giving me all that and it is fast and continuous…’ *(HCP-I, 3*). An ETNA study nurse said, ‘it (Sibel) covers a lot of vital signs measurements, and yeah, and almost as equivalent in functionality as the cardiac monitor’ *(HCP-D, 1*).

The potential for the investigational technologies to increase efficiency in monitoring was highlighted to potentially extend clinical care capacity and reduce HCP workload, which supported acceptability among healthcare professionals and caregivers. HCA and HCP emphasised the challenges of maintaining regular monitoring in busy neonatal units where the number of HCP was few in comparison to the number of neonates under their care. Speaking about the EarlySense technology, a nurse said, ‘This machine…is helping to ease the workload. Instead of placing one person to check on this baby and the other baby—one person can assess and monitor very many babies at a time because [the EarlySense technology] is doing all that work for him….[HCP] will be positive about it’ *(HCP-I, 3*). A HCA noted that the Sibel technology will be acceptable within their healthcare facility because ‘I can leave the baby on something that monitors them and have a central display screen about the patients’ vitals in real time. Then the nurses will not be as stretched taking the vitals on every single baby when they are very few*‘(HCA, 4*). Caregivers also shared that the investigational technologies would be acceptable to them because the technologies improved monitoring and clinical follow-up of their neonates.

#### Non-invasive but concerns about radiation and electrical currents

Additionally, the non-invasive design of the two investigational technologies was described by HCA, HCP and caregivers to support user satisfaction because the MCPM technologies did not appear to cause neonate discomfort. For example, a caregiver said of the EarlySense technology, ‘He will just sleep normally; it won’t affect him, but all these [vital signs] shall be recorded so I think it will be comfortable for him’ *(CG, 4*). An HCA noted, ‘when I put [the EarlySense technology under] the mattress, it won’t be inconvenient to the baby’ *(HCA, 3*). Similarly, another caregiver said of the Sibel technology, ‘I like it because the baby is comfortable when being placed on, he is not crying, I just feel he is fine’ *(CG, 3*). An HCA observed, ‘…[the Sibel technology are] such light gadgets …they are not causing any undue pressure to the baby, so they should be acceptable [to caregivers]’ *(HCA, 3*). In particular, respondents highlighted that the investigational technologies had no (EarlySense) or fewer (Sibel) attachments. For example, an HCA said of the Sibel technology, ‘What I like about it is … it doesn’t have wires. Wires bring complications’ *(HCA, 2*).

However, respondents shared that concerns about radiation and electrical currents with wireless and Bluetooth technologies may reduce acceptability, particularly among caregivers. A caregiver asked of the EarlySense technology, ‘What I want to know is maybe, does it have side effects because if it doesn’t touch him, how does it monitor? Maybe [the EarlySense technology] can cause radiation, cancer or something?’ *(CG, 4*). In reference to the Sibel technology, a caregiver also spoke of ‘the fear of transfer of dangerous waves to the body of the baby*”* (CG, 7). An ETNA study nurse shared, ‘[The caregivers] are concerned about the transfer of data from the Sibel device, both limb and chest units, to the iPad… The main concern is [that] Bluetooth uses radioactive material, so how sure are we that these devices will not harm the baby?’ *(HCP-D, 2*). An HCA described that counselling may be required to fully explain the MCPM technology and dispel misconceptions, ‘…our population may wonder is there some electrical current going through my baby’s body… but if we take our time and explain, they wouldn’t have a problem’ *(HCA, 3*).

HCA, HCP and caregivers emphasised the need for caregiver counselling and engagement to support acceptability. Different caregivers may also react differently to the use of MCPM technologies, so understanding caregiver perceptions was essential for appropriate engagement. For example, a physician said, ‘There are those who worry extremely because when they see the gadgets on the baby, they get worried. The other groups of patients think that, the more gadgets there are, the better. What is important is to explain to the mother and understand their perception of what they are seeing’ *(HCP-I, 7*). A nurse said, ‘I think they will like [the Sibel technology] but still, it depends on how we communicate about it…I believe with good communication, they will definitely embrace it’ *(HCP-I, 8*).

#### Movement and portability

HCA and HCP shared that movement and portability features could both support and/or hinder operating the technology for its intended purpose. Both of the investigational technologies were portable and could be moved throughout the neonatal unit to where they were needed. An HCA said, ‘I like the fact that [the EarlySense technology] is a portable sized tool’ *(HCA, 3*). However, while the EarlySense technology was portable, continuous monitoring was interrupted if the neonate was not calm or taken off the mattress for breastfeeding or other care needs such as diaper changing or kangaroo mother care. A nurse said of the EarlySense technology, ‘…it might present a challenge when it is feeding time…. [Mothers] will just come and take the baby off…’ *(HCP-I, 9*). An ETNA study nurse said, ‘[The EarlySense technology] should also not be used during resuscitation whereby there is a lot of movement during chest compressions. This device should be used only for calm babies…’ *(HCP-D, 2*).

The portable Sibel technology allowed for neonate movement, as one nurse said, ‘It is light, easily portable, and even with the movement of the baby, it won’t fall off. [The Sibel technology] won’t give us inaccurate results even with the movement of the baby’ *(HCP-I, 8*). However, because of its small size and highly portable design characteristic, some worried that the Sibel technology may be misplaced or stolen. An ETNA study nurse said, ‘They are very small devices which can get lost easily’ *(HCP-D, 2*). Additionally, a HCA said, ‘[the Sibel technology is] so portable and can be stolen.’*(HCA, 2*).

### Comparison of the investigational and reference technologies

Like with the investigational technologies, a major challenge of feasibility for the Masimo Rad-97 reference technology was overcrowding in the PMH neonatal unit. HCA and HCP highlighted that the stand-alone Masimo Rad-97 unit required even more space than the investigational technologies, which compromised feasibility at their facility. A nurse said, ‘We really get packed here … I feel [the Masimo Rad-97 technology] will give us more headaches because it needs more space… it will mean that every room, maybe we may have two to three tables to put it on …that will be a bit hectic’ *(HCP-I, 8*).

In contrast to the non-invasive design of the investigational technologies, HCA, HCP and caregivers highlighted that the Masimo Rad-97 technology had many wires and tubes. More attachments to the neonate was perceived to compromise neonate comfort and reduce accuracy because neonate movement may dislodge a connection, ‘Those many tubes, for babies who are a little bit active, the jumpiness of the babies can alter one or two things [and the] readings can be bad’ *(HCA, 1*). The Masimo Rad-97 technology’s nasal cannula tubing and wires were perceived by study respondents as invasive, interfering with the neonate’s movement and potentially increasing the risk of infection. For example, a HCA said, ‘All foreign objects should be treated as infection routes and I am not comfortable with that’ *(HCA, 4*). The increased number of connections also intensified the anticipated training necessary to use the Masimo Rad-97 technology properly. For example, a nurse said, ‘It has a lot of connections and tubing. If somebody is not very careful in the training, and you miss in connecting that machine, you might miss the results…’ *(HCP-I, 3*).

In addition to usability concerns, there were also acceptability concerns with caregivers. The Masimo Rad-97 technology capnography feature was especially concerning for mothers and their families as the capnography feature was associated with oxygen therapy and worsening neonate health conditions. An ETNA study nurse said, ‘It gives the picture of oxygen. Everyone knows when my baby is on oxygen, s/he is very sick…The capnography doesn't seem necessary especially for babies who are not on oxygen because everyone’s speculations at first would think you're administering oxygen’ *(HCP-D, 1*). Echoing the ETNA study nurse’s statement, a caregiver said, *‘*I thought it was oxygen. He [the father] would panic…’ *(CG, 2*). Another caregiver said, ‘Especially the pipe that goes to the nose. I would not want my child to be using it… It makes you think that the child is in a very bad state’ *(CG, 5*).

However, while the Masimo Rad-97 technology capnography feature reduced acceptability among caregivers, its familiarity in the neonatal unit may increase acceptability among some HCP. For example, a nurse said, ‘if it’s just something to insert on the nose, which is something we are familiar with, so that one can be easy…’ *(HCP-I, 5*). An ETNA study nurse said, ‘It’s familiar. It’s not a new device on the ground, so it’s familiar to me and to most HCP’ *(HCP-D, 1*). Of the three technologies, 7 of 10 caregivers rated EarlySense as the most preferable. There was more diversity of responses among health professionals but overall, the Sibel technology was most frequently favourably rated. Seven of 15 HCP who responded to the question rated the Sibel technology as their top choice among the three technologies.

## Discussion

Design factors like non-invasiveness, portability, ease of use and ability to measure multiple vital signs concurrently increased efficiency of care and supported the usability and acceptability of the investigational technologies in neonates in this resource-constrained setting. Our study of two investigational neonatal MCPM technologies within a resource-constrained, high-volume maternity hospital in sub-Saharan Africa highlighted how locally appropriate technologies can support improved neonatal care by expanding HCP capacity for monitoring and increased efficiency to quickly respond to emerging complications. Consequently, MCPM technologies can play a valuable role in improving quality of neonatal care as well as access, as more at-risk neonates are able to be identified and prioritised for intensive care. Yet, thoughtful user-friendly design factors cannot overcome basic infrastructural gaps, the need for adequate and trained HCP staffing to appropriately engage caregivers, or negate the need for regular technology service and support. Feasibility challenges of overcrowding and lack of reliable electricity, and caregiver acceptability challenges such as mistrust of wireless features (investigational technologies) or fear of capnography (reference Masimo Rad-97 technology), had implementation implications across all of the technologies within the study.

Currently, there are two reviews available of wearable continuous monitoring sensors for neonates, but these only compiled existing products and their key features.^12 13^ Acceptability and implementation factors were not explored.^12 13^ The non-adoption, abandonment, scale-up, spread and sustainability framework posits that increasingly, complexity across seven domains (health condition, technology, value, adopters, organisational capacity, wider system context and embedding/adaption over time) contributes to the non-adoption of novel health technologies.^14^ Addressing the first three domains, MCPM technologies are standard in the care of vulnerable neonates in high-resource health settings and study participants in our low-resource health setting valued their importance for improving quality of care and expressed appreciation for user-friendly design features. However, acceptability and systemic factors within their organisational and infrastructural context emerged as critical domains impacting capacity for scale-up, spread and sustainability. Our study helps to fill the current gap in understanding these domains for MCPM technologies for neonates in resource-limited settings where they are not yet routinely implemented.

In comparison to the qualitative evaluation of the investigational technologies at AKUH (Ginsburg 2021), a private, tertiary hospital in Nairobi, Kenya, there were a number of similar usability and acceptability themes. Potential harmful side effects from wireless connections and mistrust of novel technologies were voiced as concerns largely by caregivers at both hospitals. Similarly, the fears regarding the novel technologies appeared to be alleviated among some caregivers with adequate HCP explanation. The concerns around electrical fields appeared to cross socio-economic groups in Kenya as almost all of the caregivers interviewed at AKUH had university education and professional employment, compared with secondary education and lack of employment outside of the home for the majority of caregivers interviewed at PMH. Similar design features were highlighted by respondents from both PMH and AKUH to support usability of the investigational technologies, including their ease of use and ability to measure multiple vital signs as well as concerns about EarlySense technology monitoring disruptions when neonates were restless or off the mattress. Trained HCP at both hospitals were observed to effectively use the investigational technologies without difficulties.

Additionally, caregivers at both hospitals disliked the nasal capnography feature of the Masimo Rad-97 reference technology, which was associated with neonate discomfort and fears around oxygen therapy. Both AKUH and PMH groups mentioned that associations with oxygen therapy made the situation seem more dire, as if the neonate was critically ill. Caregiver anxiety around nasal oxygen and tubing also have been reported with other neonatal interventions such as bubble continuous positive airway pressure in Malawi where oxygen therapies were associated with severe illness.^15^ HCP counselling was helpful to alleviate caregiver concerns in both healthcare settings.

However, the context at AKUH was different than at PMH. AKUH had a ratio of three neonates to a nurse, reliable back-up electrical systems, a maintenance team on staff and were less reliant on donor and partner support to purchase new equipment. Consequently, equipment costs, electrical outages, technology malfunction and maintenance were not emphasised as feasibility concerns at AKUH. By contrast, all of these issues were voiced as serious concerns among PMH study respondents. Overcrowding, unreliable electricity, lack of access to computers and short staffing emerged as critical challenges to the feasibility of both the investigational and reference MCPM technologies at PMH. The identification of the general level of infrastructure and human resources are considered to be important in the development of technologies intended for use in low-income and middle-income countries (LMICs).^5^ The experience at PMH may be reflective of feasibility constraints in other large public hospitals in sub-Saharan Africa where adequate human, equipment and infrastructural resources have been identified as limiting factors in the implementation of newborn health innovations.^16 17^ The qualitative evaluations of the investigational MCPM technologies at two urban tertiary hospitals in Nairobi, Kenya also highlighted that differences between LMICs healthcare settings may be just as important as those between high-income countries and LMICs. In particular, findings from our ETNA qualitative study support existing literature on the dramatically different hospital infrastructure and human resources between private and public hospitals in Kenya,^18^ which has implications for the feasibility of effective scale-up of neonatal technologies.

A limitation of the study included that only two respondents had direct experience with the investigational and reference technologies; the HCP-I and HCA interviewed did not. Though we did not find major differences in themes reported between direct and indirect users, there is a possibility that the HCP-I interviewed may shift responses given some direct experience with the technologies. Additionally, the study was cross-sectional, which captures findings within a specific point in time. The qualitative study at PMH was conducted during the COVID-19 pandemic and a healthcare worker strike in Kenya, which may have impacted findings. Furthermore, the qualitative approach was exploratory to identify themes but the purposeful sampling design was limited in its ability to quantify their representative frequency. However, conducting IDIs with caregivers, HCP and HCA allowed an expanded understanding of feasibility, usability and acceptability from a wide range of perspectives. The triangulation of direct observations with IDIs helped to strengthen reliability of findings, and the comparison with qualitative research recently conducted with a similar methodology and the same technologies in another healthcare setting in Nairobi, Kenya helped to deepen understanding of contextual factors.

## Conclusions

MCPM technologies are an essential part of strengthening access to and quality of hospital-based neonatal care. In moving from the need to assess multiple vital signs individually and manually, MCPM technologies have the potential to enable ongoing multiparameter clinical monitoring and improve efficiency in care centrally monitored by HCP to ultimately improve health outcomes and save lives. This has implications for overburdened clinical staff attempting to provide high-quality neonatal care in resource-constrained healthcare settings. Identification of more at-risk neonates through the use of MCPM technologies also helps to improve access to the care they may require. Overall, study participants were positive about the usability of the investigational MCPM technologies but highlighted implementation challenges that require further consideration. New, innovative technologies need to be implemented within enabling environments. While thoughtful, user-friendly design factors can support usability, technology on its own cannot overcome feasibility challenges of basic infrastructural gaps and the continued need for adequate and trained staffing to effectively engage caregivers and support quality neonatal care. Innovative MCPM technologies have the potential to significantly improve neonatal care in sub-Saharan African healthcare facilities, but health system strengthening is also critical to support their sustainable uptake into routine care.

## Supplementary Material

Reviewer comments

Author's manuscript
