# Supplementary material for: Qualitative study exploring the feasibility, usability and acceptability of neonatal continuous monitoring technologies at a public tertiary hospital in Nairobi, Kenya
Source: BMJ Open. 2022 Jan 11;12(1):e053486. doi: 10.1136/bmjopen-2021-053486 (PMC8753390; doi:10.1136/bmjopen-2021-053486)
Supplement: Supplementary data [file bmjopen-2021-053486supp003.pdf]

S4 File Coding tree

Nodes	Sub-nodes	Description
A. Social-demographics information	1. Age	Age of participant
	2. Job title	Job title and current role at the facility
	3. Employment duration at facility	Duration of employment at the healthcare facility
	4. Work experience	Duration worked as a physician, nurse, technician, etc.
	5. Education	Years of education and training completed, highest level of education completed, medical background/designation (e.g., physician, nurse, technician, etc.)
	6. Healthcare provider role	Responsibilities, patient care responsibilities
B. Health system factors	1. Current constraints	Description of the current constraints to providing care to newborns at the healthcare facility. Factors that make care more difficult or easy
	2. Monitoring of newborns at the facility	Methods of newborn monitoring at the facility. How it is different (if at all) for sick newborns
	3. Access to electricity	Description of whether the facility have reliable access to electricity. The last electricity outage and how long do they typically last. What happens during power outages at the facility. How do power outages affect patient care. A back-up power supply. The process of using the backup power supply and any issues around its use (e.g., does it cover all of the equipment needed, any issues in getting permission for its use, fuel prices. Any voltage issues.
	4. Access to computers	Description of whether they have regular access to computers at this facility Whether they work well. Computers breakdown. Ways in which the computer breakdowns affect ones work as a healthcare provider
	5. Technologies used in delivery and newborn unit	Description of the technologies that are being used in the delivery and newborn care wards at this facility. Concerns or gaps in the technologies available, for maternal and newborn care at the facility. Type of healthcare providers who use the technologies. Technologies/ brands used. Whether the healthcare providers use

C. Monitoring devices	1. Familiarity with role and responsibilities with ETNA	Role with the ETNA research study and any ETNA-related responsibilities
	2. Use of continuous monitoring devices	Use of continuous monitoring devices or seen them used. Experience with continuous monitoring devices. List of devices used, how frequently one has used the types of devices. usefulness. Training received for the use of the devices.
	3. Experience with continuous monitoring devices	Description of whether continuous monitoring devices apart from the ETNA devices are used at the healthcare facility. If so, where in the facility, their purpose and frequency of use.
	4. Benefits	Benefits of using continuous monitoring devices and impacts on routine care at the facility
	5. Concerns	Any concerns about using continuous monitoring devices. Challenges to using such devices at this facility. Any situations in which the use of monitoring devices would not be useful.
	6. Need for scale up	What would be needed to scale up the use of continuous monitoring devices at the facility. Enablers that could support the process.
	7. Reaction on use of monitoring devices	Reaction of the nurses and physicians if use of continuous monitoring devices were scaled up at the facility. Reaction of caregivers (mothers, parents, guardians, etc.)
	8. Training	Any mention around training and training needs for monitoring devices in general
D. EarlySense investigational device	A. Familiarity with device	Previous experience with the device
	B. Usability	Discussions around device usability, likes and dislikes about the device, situations where the device should not be used
	C. Acceptability	Feelings of healthcare providers, administrators and caregivers about the device, whether they trusted results and if device should be incorporated
	D. Feasibility	Discussions whether the device would be suitable within their health setting
	A. Familiarity with device	Previous experience with the device

E. Sibel investigational device	B. Usability	Discussions around device usability, likes and dislikes about the device, situations where the device should not be used
	C. Acceptability	Feelings of healthcare providers, administrators and caregivers about the device, whether they trusted results and if device should be incorporated
	D. Feasibility	Discussions whether the device would be suitable within their health setting
F. Masimo RAD-97 reference device	A. Familiarity with device	Previous experience with the device
	B. Usability	Discussions around device usability, likes and dislikes about the device, situations where the device should not be used
	C. Acceptability	Feelings of healthcare providers, administrators and caregivers about the device, whether they trusted results and if device should be incorporated
	D. Feasibility	Discussions whether the device would be suitable within their health setting
G. Closing	A. Rank device	Rank of the device as the best, second best and third choice
	B. Feasibility – most appropriate device	In terms of feasibility, device (if any) that would be the most appropriate device for the healthcare facility
	C. Acceptability – most preferred device	In terms of acceptability, device (if any) that the healthcare providers and caregiver would like the best.
	D. Other comments about the devices	Any other comments about three ETNA study devices in general
	E. Any other comments about newborn monitoring devices or any other comments or concerns	Any other comments about newborn monitoring devices or any other comments or concerns
