# Supplementary material for: Qualitative study exploring the feasibility, usability and acceptability of neonatal continuous monitoring technologies at a public tertiary hospital in Nairobi, Kenya
Source: BMJ Open. 2022 Jan 11;12(1):e053486. doi: 10.1136/bmjopen-2021-053486 (PMC8753390; doi:10.1136/bmjopen-2021-053486)
Supplement: Author's manuscript [file bmjopen-2021-053486.draft_revisions.pdf]

BMJ Open

BMJ Open is committed to open peer review. As part of this commitment we make the peer review history of every article we publish publicly available.

When an article is published we post the peer reviewers' comments and the authors' responses online. We also post the versions of the paper that were used during peer review. These are the versions that the peer review comments apply to.

The versions of the paper that follow are the versions that were submitted during the peer review process. They are not the versions of record or the final published versions. They should not be cited or distributed as the published version of this manuscript.

BMJ Open is an open access journal and the full, final, typeset and author-corrected version of record of the manuscript is available on our site with no access controls, subscription charges or pay-per-view fees (http://bmjopen.bmj.com).

If you have any questions on BMJ Open's open peer review process please email info.bmjopen@bmj.com

BMJ Open

A qualitative study exploring the feasibility, usability, and acceptability of neonatal continuous monitoring technologies at a public tertiary hospital in Nairobi, Kenya

Journal:	*BMJ Open*
Manuscript ID	bmjopen-2021-053486
Article Type:	Original research
Date Submitted by the Author:	14-May-2021

Complete List of Authors:	Kinshella, Mai-Lei Woo; The University of British Columbia, Department of Obstetrics and Gynecology; BC Children's Hospital, Centre for International Child Health<br>Naanyu, Violet; Moi University, School of Arts and Sciences<br>Chomba, Dorothy; The Aga Khan University - Kenya, Department of Pediatrics<br>Waiyego, Mary ; Pumwani Maternity Hospital<br>Rigg, Jessica; BC Children's Hospital, Centre for International Child Health; The University of British Columbia, Department of Anesthesiology<br>Coleman, Jesse; Evaluation of Technologies for Neonates in Africa<br>Hwang, Bella; BC Children's Hospital, Centre for International Child Health<br>Ansermino, J. Mark; BC Children's Hospital, Centre for International Child Health; The University of British Columbia, Department of Anesthesiology<br>Macharia, William; The Aga Khan University - Kenya, Department of Pediatrics<br>ginsburg, amy sarah; University of Washington, Clinical Trial Center

Keywords:	QUALITATIVE RESEARCH, Neonatal intensive & critical care < INTENSIVE & CRITICAL CARE, International health services < HEALTH SERVICES ADMINISTRATION & MANAGEMENT

SCHOLARONE™
Manuscripts

I, the Submitting Author has the right to grant and does grant on behalf of all authors of the Work (as defined in the below author licence), an exclusive licence and/or a non-exclusive licence for contributions from authors who are: i) UK Crown employees; ii) where BMJ has agreed a CC-BY licence shall apply, and/or iii) in accordance with the terms applicable for US Federal Government officers or employees acting as part of their official duties; on a worldwide, perpetual, irrevocable, royalty-free basis to BMJ Publishing Group Ltd ("BMJ") its licensees and where the relevant Journal is co-owned by BMJ to the co-owners of the Journal, to publish the Work in this journal and any other BMJ products and to exploit all rights, as set out in our licence.

The Submitting Author accepts and understands that any supply made under these terms is made by BMJ to the Submitting Author unless you are acting as an employee on behalf of your employer or a postgraduate student of an affiliated institution which is paying any applicable article publishing charge ("APC") for Open Access articles. Where the Submitting Author wishes to make the Work available on an Open Access basis (and intends to pay the relevant APC), the terms of reuse of such Open Access shall be governed by a Creative Commons licence – details of these licences and which Creative Commons licence will apply to this Work are set out in our licence referred to above.

Other than as permitted in any relevant BMJ Author's Self Archiving Policies, I confirm this Work has not been accepted for publication elsewhere, is not being considered for publication elsewhere and does not duplicate material already published. I confirm all authors consent to publication of this Work and authorise the granting of this licence.

For peer review only

A qualitative study exploring the feasibility, usability, and acceptability of neonatal continuous monitoring technologies at a public tertiary hospital in Nairobi, Kenya

Mai-Lei Woo Kinshella[1,2*], Violet Naanyu[3], Dorothy Chomba[4], Mary Waiyego[5], Jessica Rigg[2,6], Jesse Coleman[7], Bella Hwang[2], J. Mark Ansermino[2,6], William M. Macharia[4], Amy Sarah Ginsburg[8]

[1] Department of Obstetrics and Gynecology, British Columbia Children's and Women's Hospital and The University of British Columbia, Vancouver, British Columbia, Canada
[2] Centre for International Child Health, BC Children's Hospital, Vancouver, British Columbia, Canada
[3] School of Arts and Sciences, Moi University, Eldoret, Kenya
[4] Department of Pediatrics, Aga Khan University, Nairobi, Kenya
[5] Pumwani Maternity Hospital, Nairobi, Kenya
[6] Department of Anesthesiology, The University of British Columbia, Vancouver, British Columbia, Canada
[7] Evaluation of Technologies for Neonates in Africa, United States
[8] Clinical Trial Center, University of Washington, Seattle, Washington, United States

*Corresponding Author:
Mai-Lei Woo Kinshella
Department of Obstetrics & Gynaecology
University of British Columbia
950 West 28th Avenue
Vancouver, BC, Canada, V5Z 4H4
Email: maggie.kinshella@cw.bc.ca

Word count: 5054

Keywords: continuous monitoring technologies; neonate; Africa; medical technology design; user perspectives; qualitative research

For peer review only - http://bmjopen.bmj.com/site/about/guidelines.xhtml

ABSTRACT

Objective: To assess the feasibility, usability, and acceptability of two non-invasive, multiparameter, continuous physiological monitoring (MCPM) technologies for use in neonates within a resource-constrained healthcare setting in sub-Saharan Africa.

Design: A qualitative study using in-depth interviews and direct observations to describe healthcare professional and caregiver perspectives and experiences with investigational MCPM technologies from EarlySense and Sibel compared to selected reference technologies.

Setting: Pumwani Maternity Hospital is a public, high-volume, tertiary hospital in Nairobi, Kenya.

Participants: In-depth interviews were conducted with five healthcare administrators, 12 healthcare providers, and 10 caregivers. Direct observations were made of healthcare providers using the technologies on 12 neonates overall.

Results: Design factors like non-invasiveness, portability, ease-of-use, and ability to measure multiple vital signs concurrently emerged as key themes supporting the usability and acceptability of the investigational technologies. However, respondents also reported feasibility challenges to implementation, including overcrowding in the neonatal unit, lack of reliable access to electricity and computers, and concerns about cost and maintenance needs. To improve acceptability, respondents highlighted the need for adequate staffing to appropriately engage caregivers and dispel misconceptions about the technologies.

Conclusion: Study participants were positive about the usefulness of the investigational technologies to strengthen clinical care quality and identification of at-risk neonates for better access to timely interventions. These technologies have the potential to improve equity of access to appropriate healthcare services and neonatal outcomes in sub-Saharan African healthcare facilities. However, health system strengthening is also critical to support sustainable uptake of technologies into routine care.

Trial registration: NCT03920761

For peer review only - http://bmjopen.bmj.com/site/about/guidelines.xhtml

STRENGTHS AND LIMITATIONS OF THIS STUDY

- We interviewed healthcare administrators, providers, and caregivers to understand the feasibility, usability, and acceptability of investigational technologies from multiple perspectives.
- The purposeful sampling design elicited a wide range of perspectives although these cannot be used to determine representative frequency of themes.
- The triangulation of direct observations with in-depth interviews helped to strengthen reliability of findings.
- The current study is compared with findings from a previous study conducted at a private healthcare facility in Nairobi, Kenya with the same technologies and methodology to illuminate different implementation factors between private and public tertiary hospitals.

For peer review only

BACKGROUND

Leading causes of neonatal deaths, including 35% due to preterm birth complications, 24% due to birth asphyxia and trauma, and 15% due to neonatal sepsis and infections, are preventable with quality facility-based care (1,2). However, effective implementation of evidence-based neonatal interventions may require monitoring of vital signs and time-sensitive clinical follow-up, which may be compromised in resource-constrained healthcare settings (3,4). Locally appropriate technologies to support early detection of physiologically unstable neonates requiring timely intervention have the potential to improve quality of care and neonatal health outcomes (5).

The Evaluation of Technologies for Neonates in Africa (ETNA) platform aims to boost development and optimization of promising neonatal medical technologies to be used in resource-constrained healthcare facilities. Understanding user perspectives in the intended setting is critical to medical technology design, development, deployment, and eventual uptake and acceptance. However, the feasibility, appropriateness, and acceptability of novel technologies for improving maternal and neonatal health are not often adequately investigated, thereby compromising implementation efforts (6). The ETNA platform previously conducted a qualitative evaluation of two novel, non-invasive, multiparameter, continuous physiological monitoring (MCPM) technologies developed by EarlySense and Sibel at Aga Khan University Hospital (AKUH), a private, tertiary hospital in Nairobi, Kenya where MCPM technologies were already used in neonatal intensive care (Ginsburg 2021). By contrast, Pumwani Maternity Hospital (PMH) is a public, high-volume maternity hospital in Nairobi where MCPM technologies are not routinely used. In the current study, we assessed the feasibility, usability, and acceptability of the same MCPM technologies at PMH to better understand the technologies' use for neonates within a resource-constrained healthcare setting in sub-Saharan Africa.

METHODS

Study design and setting

Comprised of in-depth interviews (IDIs) and direct observations, this descriptive qualitative study elicited perspectives and experiences of healthcare professionals and caregivers around

For peer review only - http://bmjopen.bmj.com/site/about/guidelines.xhtml

MCPM technology feasibility, usability, and acceptability. We evaluated the accuracy, reliability, and performance of novel MCPM technologies in comparison with verified reference technologies (Figure 1) and present the findings based on the "Consolidated criteria for reporting qualitative research" (COREQ) (7,8). The current study utilized the following definitions (9,10):

- Feasibility involved systemic factors required for implementation of MCPM technologies, such as hospital infrastructure, operational capacities, and functional capacities of available healthcare providers (HCP);
- Usability involved design factors that influenced HCP user experience, such as ease and efficiency of use, frequency of errors, memorability to a casual user, and user satisfaction; and
- Acceptability involved factors that influenced the willingness of healthcare administrators (HCA), HCP, and caregivers to use the technology.

PMH is a public, tertiary referral hospital serving Nairobi, Kenya and is the largest referral maternity hospital in sub-Saharan Africa with an average of 50-100 deliveries a day. Neonates in good health accompany their mothers to the postnatal ward while neonates with health complications are admitted to the neonatal unit, a large hall separated into 11 cubicles representing different diagnoses and care requirements. Neonates in more critical health conditions are placed in cubicles closest to the nursing station, while stable neonates awaiting discharge are moved to cubicles on the other side of the hall. Neonates commonly share cots and incubators with up to four neonates in each. The neonatal unit is typically staffed by three nurses and three clinical officers or physicians during the morning shift, and then two nurses and one clinical officer or physician during the afternoon and night shifts. The study moved between the different cubicles within the neonatal unit and employed two dedicated study nurses to support the study. Caregiver visitation times are restricted to every three hours for the mothers to breastfeed and care for the neonates.

Recruitment and data collection

A purposefully drawn study sample included HCA, direct and indirect HCP, and caregivers of neonates enrolled in ETNA. Direct HCP consisted of ETNA study nurses who were direct users of the MCPM technologies (HCP-D) and indirect HCP included hospital physicians, nurses, and clinical staff involved in neonatal care but who did not actively use the investigational or

For peer review only - http://bmjopen.bmj.com/site/about/guidelines.xhtml

reference MCPM technologies (HCP-I). Multiple MCPM technologies were used with each neonate enrolled in ETNA during their hospital stay. A sample size of five HCA, 12 HCP, and 10 caregivers was estimated to reach data saturation covering a wide range of perspectives from the healthcare staffing positions and caregivers available.

Study recruitment was publicized using flyers and potential participants were approached in person by a member of the qualitative study team, who introduced themselves and the ETNA study. To minimize bias, a Kenyan research consultant (VN, PhD in sociology, female) and two trained female research assistants (diplomas in health sciences) who did not know participants prior to the study activities were hired to conduct the IDIs with the enrolled qualitative study participants and the direct observations of the ETNA study nurses.

IDIs with HCA, HCP, and caregivers and direct observations of HCP-D were conducted between November 23 and December 1, 2020 following semi-structured IDI and structured observation guides (Supplementary files 1 and 2). Data collection guides were developed for the ETNA qualitative study and piloted by the Kenyan data collection team during training to refine questions. After obtaining written informed consent, 30 to 45 minute IDIs were conducted in person in a quiet, private place within PMH in English or Kiswahili, the major local languages in Kenya, depending on study participant preference. Written informed consent was obtained from HCP-D for observations, which covered three different phases of usage for each of the MCPM technologies: 1) technology preparation and initial application; 2) ongoing technology monitoring and troubleshooting; and 3) technology disconnection, removal and cleaning. IDIs were audio-recorded with permission, field notes recorded during data collection, and no repeat IDIs were conducted.

Data analysis

IDIs were transcribed verbatim and translated into English. Transcripts were uploaded into NVivo 12 software (QSR International, Melbourne, Australia) for qualitative analysis following a thematic approach. Thematic analysis involved becoming familiar with the data, generating initial codes collating identified codes into themes, and describing themes using illustrative quotes (11). A coding framework (supplementary file 3) was developed deductively from study objectives to cover feasibility, usability, and acceptability as well as inductively from emergent

For peer review only - http://bmjopen.bmj.com/site/about/guidelines.xhtml

themes by the ETNA study team (MWK, VN, DC, JR, JC, WMM, ASG). VN conducted the primary coding with review by MWK.

Data confidentiality was ensured through limiting access of study materials to authorized personnel, de-identifying participants using codes, and aggregating demographic features. Ethics approvals were obtained from Western Institutional Review Board 20 191 102 (Puyallup, Washington, USA), and the Aga Khan University Nairobi Research Ethics Committee 2019/REC-02 (v2) (Nairobi, Kenya).

Patient and public involvement

Neither patients nor public were involved in the design or conduct of the study.

RESULTS

Direct observations of HCP-D using the technologies on 12 neonates were made and IDIs conducted with 27 participants, including five HCA, 10 HCP-I (six nurses, two clinical officers, and two physicians), two HCP-D (two study nurses,), and 10 caregivers. No potential participants declined to participate. Interviewed healthcare professionals were female except for one male clinician, and ranged in age from 24 to 58 (average 36.2) years. With a median of 5 (range <1 to 35) years of work experience in the medical field, approximately half (8 of 17) of the healthcare professionals held diplomas or certificates as their highest level of formal education. Four healthcare professionals were pursuing a first degree or completed an undergraduate degree, while three held master degrees and two had medical degrees. Interviewed caregivers were female ranging in age from 19 to 28 (average 22.3) years. A majority reported that this was their first child (6 of 10 caregivers, range 1 to 3 children). Eight caregivers had secondary-level education while two had primary-level education. Most (8 of 10) caregivers reported they were unemployed or a housewife, and two caregivers shared that they were involved in informal, small-scale business. Reported occupations of husbands and partners included mason, mechanic, electrician, watchman, businessman, marketing, and driver.

Key themes reported regarding technology feasibility included the number of neonates needing monitoring, reliable access to electricity and computers, and cost and maintenance implications of the MCPM technologies. Ease and efficiency of use, non-invasiveness, and portability were

For peer review only - http://bmjopen.bmj.com/site/about/guidelines.xhtml

critical features highlighted for usability. Supporting improved monitoring capacities, concerns about radiation and electrical currents, and a need for caregiver engagement were central themes noted for the acceptability of the MCPM technologies.

Feasibility

Numbers of neonates to monitor

A major challenge at PMH was overcrowding, resulting in the common practice of multiple neonates within a single cot. As a HCA shared, *"…we are admitting so many babies but our capacity is low…the capacity of the unit is small as compared to the neonates we receive and that is why you find there are two-three-four babies in one-unit bed."* *(HCA, 1).*

HCA and HCP posited that overcrowding impacted the feasibility of scaling up individual MCPM technologies for neonates, particularly the EarlySense technology which is placed under the mattress. A study nurse said, *"We've not used [the EarlySense technology] where babies are sharing the baby cot. …we don't know of its efficiency when there's more than one [baby]…" (HCP-D, 1).* A HCA said that because the EarlySense technology *"can only take one [neonate], so it means for us we would have to prioritize really who we have to monitor so that we give them their space" (HCA, 3).* The EarlySense technology was designed for each neonate to be in an individual cot but healthcare professionals at PMH shared that this may reduce the number of neonates that could be admitted given the current practice of sharing cots.

The Sibel technology may better accommodate sharing cots as one HCA highlighted, "*sharing incubators, [the Sibel technology] is comfortable to use. I like that it is compact..." (HCA, 4).* However, overcrowding still had implications for service delivery as different neonates would need to be carefully identified and their readings easily distinguishable from one another. As a clinical officer said, A "*challenge would be telling specifically this is for this baby while you have 20 babies on this [Sibel technology]. They will need to be sure that this belongs to this baby in this room. They will need to have codes for the specific baby…" (HCP-I, 9).*

Reliable access to electricity and computers

While a back-up generator was available at PMH, HCA and HCP reported that the generators were not always functional and frequently required repairs. Electrical outages could lead to

For peer review only - http://bmjopen.bmj.com/site/about/guidelines.xhtml

delays in using technologies that required uninterrupted electricity supply, "*If there is power failure and a generator is faulty, we end up not doing what we need until electricity is back" (HCP-D, 2).*

Unreliable electricity had direct implications for the EarlySense technology, which was connected to wall power. As one nurse said, "*I saw [the EarlySense technology] is using power. So, if possible, can we have the one without the power? So that if there is no electricity we can still use it" (HCP-I, 1).* The Sibel technology used a rechargeable battery, but HCP said that ensuring the technology was fully charged when needed and charging between electrical outages would be a challenge in a busy neonatal unit. For example, a nurse said, "*... unlike other devices which you just connect to the (wall) socket and they are ready to use, [the Sibel technology has] to be prepared... So, charging them and making sure they are ready for use is a challenge for a big hospital like Pumwani" (HCP-D, 2).*

Additionally, both investigational technologies relied on the use of external screens and computers, which would require investments in equipment, spacing, and electrical infrastructure, and training for staff to use along with the current manual documentation systems. As a nurse said, "*There's no regular access to computer. There's only one, in in-charge office and… everything else is manual" (HCP-D, 1).*

Cost and maintenance

Cost and maintenance implications of the MCPM technologies were also highlighted by HCA and HCP as critical factors influencing the feasibility of potential scale-up. HCA said that a lack of funds to purchase equipment is a challenge at PMH, which is often reliant on donors and partners to fill in the gaps, "*not having funds for the equipment is a big issue because money from the county or NMS (Nairobi Metropolitan Services) is not available to us, and we have to look for donors and partners who are able to procure the equipment for us" (HCA, 4).* In addition to the initial costs of purchasing the technology, there would be additional costs around maintenance. A HCA said, "…*we have to think through how we are going to maintain this servicing. So there is a cost to it beyond the buying the purchase (HCA, 3).* Some wondered if replacement parts and the training of local biomedical engineers to service and repair the EarlySense and Sibel technologies were available in the country. Taken together with funding challenges for their initial purchase, ongoing maintenance could limit sustainable scale-up into

For peer review only - http://bmjopen.bmj.com/site/about/guidelines.xhtml

routine care as an ETNA study nurse observed, "*I have seen sometimes maybe… because of poor maintenance…it's not effective for as long as it should have been" (HCP-D, 1).*

Usability and acceptability

Direct observations of HCP-D using the MCPM technologies within the PMH neonatal unit supported usability with appropriate availability of training and support. Similar to the Masimo reference technology, application of the EarlySense and Sibel technologies to a neonate each took on average five minutes and the HCP-D were observed to not face any difficulties with preparation, initial application, monitoring, disconnection, or cleaning. No use errors where mistakes could potentially happen were observed with either investigational technology. There was one observation with each of the investigational technologies where a HCP-D required assistance from another study nurse to help calm an irritable neonate, which interfered with technology readings (EarlySense) or application (Sibel).

Ease and efficiency in use

Design factors shared by HCA and HCP that impacted user experience included that the MCPM technologies appeared easy to use and clean. Speaking of the EarlySense technology, a HCA said, "*Looks easy to clean. That is a big issue for us because we need to observe high hygiene standards" (HCA, 4).* An ETNA study nurse who used the technologies noted, "*What I liked about [the EarlySense technology] is that it's easy to place. It's quite straightforward…" (HCP-D, 1)*. A HCA said, *"[The Sibel technology] looks easy to use because you are just attaching to the extremity and the trunk" (HCA, 3).* The investigational technologies were described as easy to use for someone without extensive training.

Additionally, the MCPM technologies were described as being able to efficiently collect multiple vital signs within a single device. A clinical officer said of the EarlySense technology, "*you will be able to collect most crucial data… So you get a lot of data using a short time period" (HCP-I, 4)*. Of the Sibel technology, a nurse observed, "*…It is taking four vitals at the same time, whereas if it is manual, I would have four gadgets…[such as] stethoscope, thermometers… Now that small gadget I just place it on the chest…it is giving me all that and it is fast and continuous…"(HCP-I, 3).* An ETNA study nurse said, *"it (Sibel) covers a lot of vital signs*

For peer review only - http://bmjopen.bmj.com/site/about/guidelines.xhtml

measurements, and yeah, and almost as equivalent in functionality as the cardiac monitor" (HCP-D, 1).

The potential for the investigational technologies to increase efficiency in monitoring was highlighted to potentially extend clinical care capacity and reduce HCP workload, which supported acceptability among healthcare professionals and caregivers. HCA and HCP emphasized the challenges of maintaining regular monitoring in busy neonatal units where the number of HCP were few in comparison to the number of neonates under their care. Speaking about the EarlySense technology, a nurse said, "*This machine…is helping to ease the workload. Instead of placing one person to check on this baby and the other baby—one person can assess and monitor very many babies at a time because [the EarlySense technology] is doing all that work for him….[HCP] will be positive about it" (HCP-I, 3).* A HCA noted that the Sibel technology will be acceptable within their healthcare facility because "*I can leave the baby on something that monitors them and have a central display screen about the patients' vitals in real time. Then the nurses will not be as stretched taking the vitals on every single baby when they are very few" (HCA, 4).* Caregivers also shared that the investigational technologies would be acceptable to them because the technologies improved monitoring and clinical follow-up of their neonates.

<u>Non-invasive but concerns about radiation and electrical currents</u>

Additionally, the non-invasive design of the two investigational technologies was described by HCA, HCP, and caregivers to support user satisfaction because the MCPM technologies did not appear to cause neonate discomfort. For example, a caregiver said of the EarlySense technology, "*He will just sleep normally; it won't affect him, but all these [vital signs] shall be recorded so I think it will be comfortable for him" (CG, 4).* An HCA noted, *"when I put [the EarlySense technology under] the mattress, it won't be inconvenient to the baby" (HCA, 3).* Similarly, another caregiver said of the Sibel technology, "*I like it because the baby is comfortable when being placed on, he is not crying, I just feel he is fine" (CG, 3).* An HCA observed, *"…[the Sibel technology are] such light gadgets …they are not causing any undue pressure to the baby, so they should be acceptable [to caregivers]" (HCA, 3).* In particular, respondents highlighted that the investigational technologies had no (EarlySense) or fewer (Sibel) attachments. For example,

For peer review only - http://bmjopen.bmj.com/site/about/guidelines.xhtml

an HCA said of the Sibel technology, "*What I like about it is … it doesn't have wires. Wires bring complications" (HCA, 2).*

However, respondents shared that concerns about radiation and electrical currents with wireless and Bluetooth technologies may reduce acceptability, particularly among caregivers. A caregiver asked of the EarlySense technology, *"What I want to know is maybe, does it have side effects because if it doesn't touch him, how does it monitor? Maybe [the EarlySense technology] can cause radiation, cancer or something?" (CG, 4).* In reference to the Sibel technology, a caregiver also spoke of "*the fear of transfer of dangerous waves to the body of the baby"* (CG, 7). An ETNA study nurse shared, "*[The caregivers] are concerned about the transfer of data from the Sibel device, both limb and chest units, to the iPad… The main concern is [that] Bluetooth uses radioactive material, so how sure are we that these devices will not harm the baby?" (HCP-D, 2).* An HCA described that counselling may be required to fully explain the MCPM technology and dispel misconceptions, *"…our population may wonder is there some electrical current going through my baby's body… but if we take our time and explain, they wouldn't have a problem" (HCA, 3).*

HCA, HCP, and caregivers emphasized the need for caregiver counselling and engagement to support acceptability. Different caregivers may also react differently to the use of MCPM technologies, so understanding caregiver perceptions was essential for appropriate engagement. For example, a physician said, "*There are those who worry extremely because when they see the gadgets on the baby, they get worried. The other groups of patients think that, the more gadgets there are, the better. What is important is to explain to the mother and understand their perception of what they are seeing" (HCP-I, 7).* A nurse said, "*I think they will like [the Sibel technology] but still, it depends on how we communicate about it…I believe with good communication, they will definitely embrace it" (HCP-I, 8).*

<u>Movement and portability</u>

HCA and HCP shared that movement and portability features could both support and/or hinder operating the technology for its intended purpose. Both of the investigational technologies were portable and could be moved throughout the neonatal unit to where they were needed. An HCA said, *"I like the fact that [the EarlySense technology] is a portable sized tool" (HCA, 3).* However, while the EarlySense technology was portable, continuous monitoring was interrupted

For peer review only - http://bmjopen.bmj.com/site/about/guidelines.xhtml

if the neonate was not calm or taken off the mattress for breastfeeding or other care needs such as diaper changing or kangaroo mother care. A nurse said of the EarlySense technology, "*…it might present a challenge when it is feeding time…. [Mothers] will just come and take the baby off…" (HCP-I, 9).* An ETNA study nurse said, "*[The EarlySense technology] should also not be used during resuscitation whereby there is a lot of movement during chest compressions. This device should be used only for calm babies…" (HCP-D, 2).*

The portable Sibel technology allowed for neonate movement, as one nurse said, "*It is light, easily portable, and even with the movement of the baby, it won't fall off. [The Sibel technology] won't give us inaccurate results even with the movement of the baby" (HCP-I, 8).* However, because of its small size and highly portable design characteristic, some worried that the Sibel technology may be misplaced or stolen. An ETNA study nurse said, "*They are very small devices which can get lost easily" (HCP-D, 2).* Additionally, a HCA said, "[the Sibel technology is] *so portable and can be stolen."(HCA, 2).*

Comparison of the investigational and reference technologies

Like with the investigational technologies, a major challenge of feasibility for the Masimo Rad-97 reference technology was overcrowding in the PMH neonatal unit. HCA and HCP highlighted that the stand-alone Masimo Rad-97 unit required even more space than the investigational technologies, which compromised feasibility at their facility. A nurse said, *"We really get packed here … I feel [the Masimo Rad-97 technology] will give us more headaches because it needs more space… it will mean that every room, maybe we may have two to three tables to put it on …that will be a bit hectic" (HCP-I, 8).*

In contrast to the non-invasive design of the investigational technologies, HCA, HCP, and caregivers highlighted that the Masimo Rad-97 technology had many wires and tubes. More attachments to the neonate was perceived to compromise neonate comfort and reduce accuracy because neonate movement may dislodge a connection, "*Those many tubes, for babies who are a little bit active, the jumpiness of the babies can alter one or two things [and the] readings can be bad" (HCA, 1).* The Masimo Rad-97 technology's nasal cannula tubing and wires were perceived by study respondents as invasive, interfering with the neonate's movement and potentially increasing the risk of infection. For example, a HCA said, *"All foreign objects should be treated as infection routes and I am not comfortable with that" (HCA, 4).* The increased

For peer review only - http://bmjopen.bmj.com/site/about/guidelines.xhtml

number of connections also intensified the anticipated training necessary to use the Masimo Rad-97 technology properly. For example, a nurse said, "*It has a lot of connections and tubing. If somebody is not very careful in the training, and you miss in connecting that machine, you might miss the results…" (HCP-I, 3).*

In addition to usability concerns, there were also acceptability concerns with caregivers. The Masimo Rad-97 technology capnography feature was especially concerning for mothers and their families as the capnography feature was associated with oxygen therapy and worsening neonate health conditions. An ETNA study nurse said, "*It gives the picture of oxygen. Everyone knows when my baby is on oxygen, s/he is very sick…The capnography doesn't seem necessary especially for babies who are not on oxygen because everyone's speculations at first would think you're administering oxygen" (HCP-D, 1).* Echoing the ETNA study nurse's statement, a caregiver said*, "I thought it was oxygen. He [the father] would panic…" (CG, 2).* Another caregiver said, "*Especially the pipe that goes to the nose. I would not want my child to be using it… It makes you think that the child is in a very bad state" (CG, 5).*

However, while the Masimo Rad-97 technology capnography feature reduced acceptability among caregivers, its familiarity in the neonatal unit may increase acceptability among some HCP. For example, a nurse said, "*if it's just something to insert on the nose, which is something we are familiar with, so that one can be easy..." (HCP-I, 5).* An ETNA study nurse said, "*It's familiar. It's not a new device on the ground, so it's familiar to me and to most HCP" (HCP-D, 1).*

DISCUSSION

Design factors like non-invasiveness, portability, ease of use, and ability to measure multiple vital signs concurrently increased efficiency of care and supported the usability and acceptability of the investigational technologies in neonates in this resource-constrained setting. Our study of two investigational neonatal MCPM technologies within a resource-constrained, high-volume maternity hospital in sub-Saharan Africa highlighted how locally appropriate technologies can support improved neonatal care by expanding HCP capacity for monitoring and increased efficiency to quickly respond to emerging complications. Consequently, MCPM technologies can play a valuable role in improving quality of neonatal care as well as access, as more at-risk

For peer review only - http://bmjopen.bmj.com/site/about/guidelines.xhtml

neonates are able to be identified and prioritized for intensive care. Yet, thoughtful user-friendly design factors cannot overcome basic infrastructural gaps, the need for adequate and trained HCP staffing to appropriately engage caregivers, or negate the need for regular technology service and support. Feasibility challenges of overcrowding and lack of reliable electricity, and caregiver acceptability challenges such as mistrust of wireless features (investigational technologies) or fear of capnography (reference Masimo Rad-97 technology), had implementation implications across all of the technologies within the study.

In comparison to the qualitative evaluation of the investigational technologies at AKUH (Ginsburg 2021), a private, tertiary hospital in Nairobi, Kenya, there were a number of similar usability and acceptability themes. Potential harmful side effects from wireless connections and mistrust of novel technologies were voiced as concerns largely by caregivers at both hospitals. Similarly, the fears regarding the novel technologies appeared to be alleviated among some caregivers with adequate HCP explanation. The concerns around electrical fields appeared to cross socio-economic groups in Kenya as almost all of the caregivers interviewed at AKUH had university education and professional employment, compared to secondary education and lack of employment outside of the home for the majority of caregivers interviewed at PMH. Similar design features were highlighted by respondents from both PMH and AKUH to support usability of the investigational technologies, including their ease of use and ability to measure multiple vital signs as well as concerns about EarlySense technology monitoring disruptions when neonates were restless or off the mattress. Trained HCP at both hospitals were observed to effectively use the investigational technologies without difficulties.

Additionally, caregivers at both hospitals disliked the nasal capnography feature of the Masimo Rad-97 reference technology, which was associated with neonate discomfort and fears around oxygen therapy. Both AKUH and PMH groups mentioned that associations with oxygen therapy made the situation seem more dire, as if the neonate was critically ill. Caregiver anxiety around nasal oxygen and tubing also have been reported with other neonatal interventions such as bubble continuous positive airway pressure in Malawi where oxygen therapies were associated with severe illness (12). HCP counselling was helpful to alleviate caregiver concerns in both healthcare settings.

For peer review only - http://bmjopen.bmj.com/site/about/guidelines.xhtml

However, the context at AKUH was different than at PMH. AKUH had a ratio of three neonates to a nurse, reliable back-up electrical systems, a maintenance team on staff, and were less reliant on donor and partner support to purchase new equipment. Consequently, equipment costs, electrical outages, technology malfunction, and maintenance were not emphasized as feasibility concerns at AKUH. By contrast, all of these issues were voiced as serious concerns among PMH study respondents. Overcrowding, unreliable electricity, lack of access to computers, and short staffing emerged as critical challenges to the feasibility of both the investigational and reference MCPM technologies at PMH. The identification of the general level of infrastructure and human resources are considered to be important in the development of technologies intended for use in low- and middle-income countries (LMICs) (5). The qualitative evaluations of the investigational MCPM technologies at two urban tertiary hospitals in Nairobi, Kenya highlighted that differences between LMICs healthcare settings may be just as important as those between high-income countries and LMICs. In particular, findings from our ETNA qualitative study support existing literature on the dramatically different hospital infrastructure and human resources between private and public hospitals in Kenya (13), which has implications for the feasibility of effective scale-up of neonatal technologies.

A limitation of the study included that only two respondents had direct experience with the investigational and reference technologies; the HCP-I and HCA interviewed did not. Though we did not find major differences in themes reported between direct and indirect users, there is a possibility that the HCP-I interviewed may shift responses given some direct experience with the technologies. Additionally, the study was cross-sectional, which captures findings within a specific point in time. The qualitative study at PMH was conducted during the COVID-19 pandemic and a healthcare worker strike in Kenya, which may have impacted findings. Furthermore, the qualitative approach was exploratory to identify themes but the purposeful sampling design was limited in its ability to quantify their representative frequency. However, conducting IDIs with caregivers, HCP, and HCA allowed an expanded understanding of feasibility, usability, and acceptability from a wide range of perspectives. The triangulation of direct observations with IDIs helped to strengthen reliability of findings, and the comparison with qualitative research recently conducted with a similar methodology and the same technologies in another healthcare setting in Nairobi, Kenya helped to deepen understanding of contextual factors.

For peer review only - http://bmjopen.bmj.com/site/about/guidelines.xhtml

CONCLUSIONS

MCPM technologies are an essential part of strengthening access to and quality of hospital-based neonatal care. In moving from the need to assess multiple vital signs individually and manually, MCPM technologies have the potential to enable ongoing multiparameter clinical monitoring and improve efficiency in care centrally monitored by HCP to ultimately improve health outcomes and save lives. This has implications for overburdened clinical staff attempting to provide high-quality neonatal care in resource-constrained healthcare settings. Identification of more at-risk neonates through the use of MCPM technologies also helps to improve access to the care they may require. Overall, study participants were positive about the usability of the investigational MCPM technologies but highlighted implementation challenges that require further consideration. New, innovative technologies need to be implemented within enabling environments. While thoughtful, user-friendly design factors can support usability, technology on its own cannot overcome feasibility challenges of basic infrastructural gaps and the continued need for adequate and trained staffing to effectively engage caregivers and support quality neonatal care. Innovative MCPM technologies have the potential to significantly improve neonatal care in sub-Saharan African healthcare facilities, but health system strengthening is also critical to support their sustainable uptake into routine care.

FOOTNOTES

Acknowledgements: The authors are grateful to the health professionals and caregivers for sharing their thoughts and experiences with us and the local hospital and community for their support. We would also like to thank Marianne Vidler for her insights on study design and the data collection tools as well as the research team who collected and managed the data without whom this study would not have been possible.

Contributors: ASG, JMA, and WMM conceptualized the ETNA platform. ASG supervised this qualitative study and designed the data collection instruments with critical input and support from VN, JR, JC, BH, and WMM. Among the authors, VN, DC, and WMM were responsible for data collection and local project administration. MWK and VN performed the analyses. MWK

For peer review only - http://bmjopen.bmj.com/site/about/guidelines.xhtml

and ASG wrote the first draft of the manuscript. All authors provided feedback and review of the manuscript.

Funding: This work is supported by grants from the Bill & Melinda Gates Foundation (BMGF) (OPP1203136) and the Save the Children Innovation Fund. Following BMGF input into trial design, funders had no role in data collection, analysis, or interpretation, or writing of the report.

Conflicts of interest: None declared.

Ethics approval: Ethics approvals were obtained from Western Institutional Review Board 20 191 102 (Puyallup, Washington, USA), and the Aga Khan University Nairobi Research Ethics Committee 2019/REC-02 (v2) (Nairobi, Kenya).

Data sharing statement: De-identified data are available on request.

For peer review only

REFERENCES

1. Strong KL, Pedersen J, White Johansson E, Cao B, Diaz T, Guthold R, et al. Patterns and trends in causes of child and adolescent mortality 2000–2016: setting the scene for child health redesign. BMJ Glob Heal. 2021 Mar 17;6(3):e004760.

2. Lawn JE, Kinney M V, Belizan JM, Mason EM, McDougall L, Larson J, et al. Born too soon: accelerating actions for prevention and care of 15 million newborns born too soon. Reprod Health. 2013;10 Suppl 1(Suppl 1):S6.

3. Kinshella M-LW, Salimu S, Chiwaya B, Chikoti F, Chirambo L, Mwaungulu E, et al. "So sometimes, it looks like it's a neglected ward": Health worker perspectives on implementing kangaroo mother care in southern Malawi. Gurgel RQ, editor. PLoS One. 2020 Dec 17;15(12):e0243770.

4. Nyondo-Mipando AL, Kinshella MLW, Bohne C, Suwedi-Kapesa LC, Salimu S, Banda M, et al. Barriers and enablers of implementing bubble Continuous Positive Airway Pressure (CPAP): Perspectives of health professionals in Malawi. Ameh CA, editor. PLoS One. 2020 Feb 13;15(2):e0228915.

5. Maynard KR, Causey L, Kawaza K, Dube Q, Lufesi N, Maria Oden Z, et al. New technologies for essential newborn care in under-resourced areas: what is needed and how to deliver it. Paediatr Int Child Health. 2015 Aug 8;35(3):192–205.

6. Lunze K, Higgins-Steele A, Simen-Kapeu A, Vesel L, Kim J, Dickson K. Innovative approaches for improving maternal and newborn health - A landscape analysis. BMC Pregnancy Childbirth. 2015 Dec 17;15(1):1–19.

7. Tong A, Sainsbury P, Craig J. Consolidated criteria for reporting qualitative research (COREQ): a 32-item checklist for interviews and focus groups. Int J Qual Heal Care. 2007 Sep 16;19(6):349–57.

8. Ginsburg AS, Nkwopara E, MacHaria W, Ochieng R, Waiyego M, Zhou G, et al. Evaluation of non-invasive continuous physiological monitoring devices for neonates in Nairobi, Kenya: A research protocol. BMJ Open. 2020 Apr 12;10(4):35184.

9. Ginsburg AS, Tawiah Agyemang C, Ambler G, Delarosa J, Brunette W, Levari S, et al. mPneumonia, an Innovation for Diagnosing and Treating Childhood Pneumonia in Low-Resource Settings: A Feasibility, Usability and Acceptability Study in Ghana. Simeoni U, editor. PLoS One. 2016 Oct 27;11(10):e0165201.

10. Nielson J. Usability engineering. San Diego, CA: Academic Press Inc.; 1993.

11. Braun V, Clarke V. Using thematic analysis in psychology. Qual Res Psychol. 2006;3(2):77–101.

12. Salimu S, Kinshella MLW, Vidler M, Banda M, Newberry L, Dube Q, et al. Health workers' views on factors affecting caregiver engagement with bubble CPAP. BMC Pediatr. 2020 Apr 23;20(1):180.

13. Gathara D, Serem G, Murphy GAV, Obengo A, Tallam E, Jackson D, et al. Missed nursing care in newborn units: a cross-sectional direct observational study. BMJ Qual Saf.

For peer review only - http://bmjopen.bmj.com/site/about/guidelines.xhtml

2020 Jan 1;29(1):19–30.

For peer review only

For peer review only - http://bmjopen.bmj.com/site/about/guidelines.xhtml

Figure 1. Overview of the three multiparameter continuous physiological monitoring technologies

INVESTIGATIONAL TECHNOLOGIES	
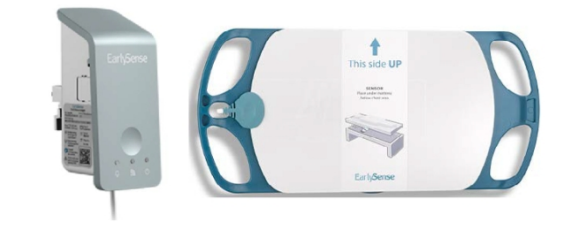	**EarlySense**<br>- Non-contact mattress pad attached to main power outlet<br>- Continuous monitoring of respiratory rate and heart rate wirelessly transmitted to an external screen<br>- Commercially available and used in health facilities in Europe and North America for adult and pediatric patients
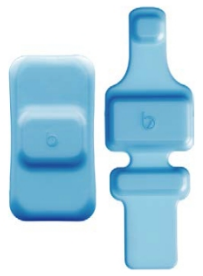	**Sibel**<br>- Set of two wireless, reusable, self-contained, direct contact skin sensors with rechargeable batteries<br>- Together, chest and limb sensors monitor respiratory rate heart rate, bio-impedance, pulse oximtery, and movement<br>- Disposable hydrogel adhesive and Velcro bands keep sensors on the neonate<br>- Data saved locally on sensor and with live showing of vital signs via iPad
REFERENCE TECHNOLOGY	
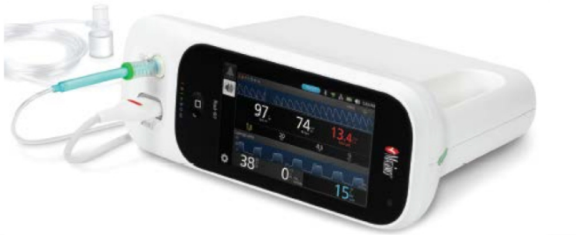	**Masimo Rad-97**<br>- Disposable skin sensor for heart rate and pulse oximetry<br>- Nasal capnography records carbon dioxide levels and measures respiratory rate<br>- Commercially available and extensively used worldwide in patients of all ages

Overview of three multiparameter continuous physiological monitoring technologies

216x224mm (150 x 150 DPI)

S2 File ETNA Qualitative Study Interview Guides

2.1 In-Depth Interview Guide – Caregiver

**Administrative information**	
**Caregiver ID number:**	**Sex:** ☐ Female ☐ Male
**Date caregiver informed consent form (ICF) signed:** |D|D| - |M|M|M| - |Y|Y|Y|Y|	
**Caregiver ICF signed prior to any study questions?** ☐ Yes ☐ No	
**Name of research staff who explained the ICF:**	
**Does the caregiver agree to be audio recorded?** ☐ Yes ☐ No<br>If Yes, was the interview audio recorded? ☐ Yes ☐ No<br>If No, why was the interview not recorded? ______________________________	
**Name of interviewer:**	
**Date of interview:** |D|D| - |M|M|M| - |Y|Y|Y|Y|	
**Location of interview:**<br>☐ **Aga Khan University – Nairobi Hospital**<br>☐ **Pumwani Maternity Hospital**	
**Interview start time:** |H|H| : |M|M| *military time*	

Instructions for qualitative research staff:

Use this document as a guide to conduct the interviews with the **caregivers**.
Conduct the interview in the language with which the caregiver feels most comfortable.
The interview should take place in a quiet place that allows privacy.
Please introduce each question separately. The interview must flow as a conversation. If you notice that the caregiver is hesitant in answering, does not give an in-depth response, or the response is not satisfactory, please probe or ask follow-up questions, but do NOT prompt any specific answer. Several probes are suggested, and you may also ask follow-up questions that are not listed in this guide but are necessary for the complete expression of the caregiver's views.
Please **record the interview** using the audio recorder (if caregiver consent is provided) and state the ETNA Caregiver ID number.
All comments from the caregiver should be recorded/written verbatim.
Please cross-check the narratives written with the recorded version as a reference and correct as necessary.
All responses must be kept confidential. Do not discuss or share responses with anyone outside of the ETNA study team.

Script to initiate the interview

"Hello, my name is __________ and I am a researcher with the ETNA project and we are evaluating monitoring devices for newborns. We want to hear about your experiences and learn from your thoughts and feelings. We will keep what you tell us today confidential, which means that nothing you say will be directly linked to you so please feel free to share. If you feel uncomfortable with any questions, let me know and we will skip it. Before we start, do you have any questions for me? Is it ok to begin? Thank you, I will start the audio-recording now."

A. Demographic information

For peer review only

1.	First, we will start with some questions about yourself, what is your age?
2.	Did you attend any schooling? If so, what class (level) did you complete?
3.	Would you be able to tell us a little about yourself and what you/your husband do for a living?
4.	Where do you and your family live? How far away is it from this hospital?
5.	How many people live together in your house and what is their relationship to you?
6.	How many children do you have? What is your role in caring for your newborn?

B. Birth history, pregnancy and healthcare facility experience

If this some of this information could be abstracted from patient hospital records, could consider using these records as a resource BEFORE the interview. Otherwise, please ask these questions.

1. How many pregnancies have you (or your wife, daughter-in-law, daughter) had? How many live births?
2. We would like to learn more about your experience with your most recent pregnancy. Could you tell us if you had any issues or complications during the pregnancy, labor or delivery?

Probes: What were your symptoms during pregnancy, length of labor, mode of delivery, how long admitted to the hospital?

3. Was your baby born early? If yes, do you know how early? (Another way to phrase this is "When were you expecting the baby and was the baby born earlier?")
4. Did your newborn have any health issues when he/she was born? If yes, what were they?

Probes: Examples include low birth weight (kangaroo mother care), infection at time of birth, birth defect, respiratory distress (trouble breathing), neonatal jaundice (put under the blue light), inability to breastfeed, etc.

5. What healthcare services did you and your newborn receive here at the hospital?
6. Are you happy with the quality of care you and your newborn received at this hospital? Could you explain with an example? What do you think could make the quality of care at this hospital better?

Probes: How did the staff treat you and your family? Did they seem trained/knowledgeable? Did they have enough equipment/supplies to care for you and your newborn?

7. How did you get to this hospital and how long did it take you to get here from your home? Why did you and your family decide to come to this hospital for delivery (or newborn care depending on their narrative)?
8. What other health facilities do you usually go to when you or your family needs medical care? When do you go to those other health facilities instead of this hospital?

C. Monitoring devices

1. What are your experiences with how healthcare providers monitor newborns receiving care at this hospital? How often do they come by to check your newborn and what do they usually check?
2. Do healthcare providers use any devices or technologies when they are doing a checkup on your newborn?
3. Are there any devices or machines that you are aware of that are used to monitor the newborn between checks by the healthcare provider?
4. Do you have any concerns about these devices? If so, could you explain with an example?

D. EarlySense InSight investigational device

Research staff shows EarlySense InSight device to the caregiver and explains how it works.

"The EarlySense InSight device is a contact-free newborn monitoring system. The system includes a sensor pad that is placed under the newborn's mattress to measure heart rate, breathing rate, motion, and sleep status. There is no physical contact between the newborn and the sensor pad. Information from the sensor pad is continuously transmitted to a monitor or tablet that can be read by hospital staff."

For peer review only

Allow them to touch the InSight device, the mattress pad and the cable that goes between them.

1. Have you ever seen this device before? Was this device used in the care of your newborn?
   If yes,
   a. What was the first thought that came to your mind when the healthcare provider told you about this device? How did you feel about this device being used for your newborn? How do you feel about it now? How did your husband (or family) react when they learned that your newborn was on this device?
   b. What did you like (if anything) about this device and how it was used? What did you dislike (if anything) about this device and how it was used? Please explain.
   c. Did the healthcare provider using the device run into any difficulties? What did they do?

   If no,
   a. Imagine if a doctor recommended using this device for your newborn, how would you feel? What do you think your husband (or family) would think if your newborn was put on this device?
2. Are there any problems you can think of with this device? Any concerns or parts/features you think might be harmful to newborns? In what situations? Please explain.
3. Do you think this device should be used in the care of newborns in this hospital? Why or why not?
4. Do you see any problems with using this device at this hospital? If so, could you explain with an example?
5. If there was a healthcare facility that used this device regularly to help care for newborns, would that make you want to go to that facility more or less? Why?

E. Sibel ANNE investigational device

Research staff shows Sibel ANNE device to caregiver and explains how it works.
"The Sibel ANNE device uses non-invasive sensors to continuously measure and record a newborn's heart rate, breathing rate, level of oxygen in the blood, and skin temperature. One sensor is attached to the newborn's chest and contains a battery. The second sensor is battery-free, ultra-thin, and is applied to the newborn's hand or foot. Information from the sensors is wirelessly transmitted to a monitor or tablet that can be read by hospital staff."
Allow them to touch the ANNE chest and limb sensors, the hydrogel, and iPad display fully.

1. Have you ever seen this device before? Was this device used in the care of your newborn?
   If yes,
   a. What was the first thought that came to your mind when the healthcare provider told you about this device? How did you feel about this device being used for your newborn? How do you feel about it now? How did your husband (or family) react when they learned that your newborn was on this device?
   b. What did you like (if anything) about this device and how it was used? What did you dislike (if anything) about this device and how it was used? Please explain.
   c. Did the healthcare provider using the device run into any difficulties? What did they do?

   If no,
   a. Imagine if a doctor recommended using this device for your newborn, how would you feel? What do you think your husband (or family) would think if your newborn was put on this device?
2. Are there any problems you can think of with this device? Any concerns or parts/features you think might be harmful to newborns? In what situations? Please explain.
3. Do you think this device should be used in the care of newborns in this hospital? Why or why not?
4. Do you see any problems with using this device at this hospital? If so, could you explain with an example?
5. If there was a healthcare facility that used this device regularly to help care for newborns, would that make you want to go to that facility more or less? Why

F. Masimo RAD-97 reference device
**Research staff shows Masimo Rad-97 device to caregiver and explains how it works.**<br>*"The Masimo Rad-97 is a non-invasive device that measures a newborn's heart rate, breathing rate, and level of oxygen in the blood. Information is collected through a skin sensor that is applied to the newborn's hand or foot and a tube that is inserted into the newborn's nostrils. The information is then continuously transmitted to a monitor that can be read by hospital staff."*<br>**Allow them to touch the Rad-97, skin sensor and capnography tube fully.**<br>1. Have you ever seen this device before? Was this device used in the care of your newborn?<br>**If yes,**<br>a. What was the first thought that came to your mind when the healthcare provider told you about this device? How did you feel about this device being used for your newborn? How do you feel about it now? How did your husband (or family) react when they learned that your newborn was on this device?<br>b. What did you like (if anything) about this device and how it was used? What did you dislike (if anything) about this device and how it was used? Please explain.<br>c. Did the healthcare provider using the device run into any difficulties? What did they do?<br>**If no,**<br>a. Imagine if a doctor recommended using this device for your newborn, how would you feel? What do you think your husband (or family) would think if your newborn was put on this device?<br>2. Are there any problems you can think of with this device? Any concerns or parts/features you think might be harmful to newborns? In what situations? Please explain.<br>3. Do you think this device should be used in the care of newborns in this hospital? Why or why not?<br>4. Do you see any problems with using this device at this hospital? If so, could you explain with an example?<br>5. If there was a healthcare facility that used this device regularly to help care for newborns, would that make you want to go to that facility more or less? Why

H. Closing
1. Taking into consideration the monitoring devices we have talked about today, could you rank the device (if any) you think is the best, second best and third choice in your opinion? Please explain why.<br>2. Do you have any other comments about any of the three devices that we did not talk about?<br>3. Do you have any other comments about newborn monitoring devices or any other comments or concerns overall that we did not get to talk about?
"Thank you for your time and the helpful information you have provided. Your feedback, along with feedback from other people we talk to will be used to recommend solutions for better care."
**Interview end time:** |H|H| : |M|M| *military time*

2.2 In-Depth Interview Guide – Healthcare Administrator (HCA)

Administrative information	
**HCA ID number:**	**Sex:** ☐ Female ☐ Male
**Date HCA informed consent form (ICF) signed:** |D|D| - |M|M|M| - |Y|Y|Y|Y|	
**HCA ICF signed prior to any study questions?** ☐ Yes ☐ No	

For peer review only - http://bmjopen.bmj.com/site/about/guidelines.xhtml

**Name of research staff who explained the ICF:**
**Does the HCA agree to be audio recorded?** ☐ Yes ☐ No<br>If Yes, was the interview audio recorded? ☐ Yes ☐ No<br>If No, why was the interview not recorded? ______________________________
**Name of interviewer:**
**Date of interview:** |D|D| - |M|M|M| - |Y|Y|Y|Y|
**Location of interview:**<br>☐ **Aga Khan University – Nairobi Hospital**<br>☐ **Pumwani Maternity Hospital**<br>☐ **Other: ____________________________**
**Interview start time:** |H|H| : |M|M| *military time*

<u>Instructions for qualitative research staff</u>:

- Use this document as a guide to conduct the interviews with the **healthcare administrators (HCA)**.
- Conduct the interview in the language with which the HCA feels most comfortable.
- The interview should take place in a quiet place that allows privacy.
- Please introduce each question separately. The interview should flow as a conversation. If you notice that the HCA is hesitant in answering, does not give an in-depth response, or the response is not satisfactory, please probe or ask follow-up questions, but do NOT prompt any specific answer. Several probes are suggested, and you may also ask follow-up questions that are not listed in this guide but are necessary for the complete expression of the HCA's views.
- Please **record the interview** using the audio recorder (if HCA consent is provided) and state the ETNA HCA ID number.
- All comments from the HCA should be recorded/written verbatim.
- Please cross-check the narratives written with the recorded version as a reference and correct as necessary.
- All responses must be kept confidential. Do not discuss or share responses with anyone outside of the ETNA study team.

Script to initiate the interview

"Hello, my name is ___________ and I am a researcher with the ETNA project and we are evaluating monitoring devices for newborns. We want to hear about your experiences and learn from your thoughts and feelings. We will keep what you tell us today confidential, which means that nothing you say will be directly linked to you so please feel free to share. If you feel uncomfortable with any questions, let me know and we will skip it. Before we start, do you have any questions for me? Is it ok to begin? Thank you, I will start the audio-recording now."

G. Demographic information

1. First, we will start with some questions about yourself, what is your age?
2. How many years of education and training have you received and what is your highest level of education completed? What is your medical background (e.g., doctor, nurse, technician, etc.)? Were you ever involved in patient care? In the care of newborns?
3. How long did you work in the medical field before working as a healthcare administrator? How long have you been working as a healthcare administrator?

H. Healthcare administrator role

For peer review only

1. What is your job title and current role here at this healthcare facility? How long (years, months if less than one year) have you been in the current position at this facility?
2. What are your responsibilities as a healthcare administrator at this facility?
Probes: What is your involvement (if any) in policy development for newborn care such as creating new protocols and/or adapting national guidelines? Please share what a typical day as a hospital administrator would be like for you.

I. Facility

1. What is the process of purchasing medical equipment at this healthcare facility?
Probes: Who makes the decision to identify what medical equipment will be used in the hospital? Who makes the decisions on what to purchase? Are these decisions made on an individual hospital basis or decided at a local or national level by Ministry of Health?
2. What are the current constraints (if any) to providing care to newborns at this facility?
Probes: What makes care more difficult? What would make it easier?
3. Does this facility have reliable access to electricity? When was the last electricity outage and how long do they typically last? What happens during power outages at your facility?
Probes: How do power outages affect patient care? Is there a back-up power supply? If so, what is the process of using the backup power supply and are there any issues around its use (e.g., does it cover all of the equipment needed, any issues in getting permission for its use, fuel prices? Any voltage issues?)
4. Are you aware of any technologies that are being used in the delivery and newborn care wards at this facility, and if so, can you describe them? What are some concerns you have, or gaps in the technologies available, for maternal and newborn care at this facility?
Probes: Which healthcare providers use the technologies? What technologies/brands are used? Do healthcare providers use any handheld or portable devices for maternal or newborn care (e.g., tablets or Smartphones)? If yes, please describe the technologies and their use.

J. Monitoring devices

We would especially like to learn about your perspectives on continuous monitoring devices.

1. Before this study, had you used continuous monitoring devices or seen them in use? Are you aware of any continuous monitoring devices being used at this healthcare facility outside of the ETNA study?
Probes: If yes, where in the facility? For what purpose? How frequently are they used?
2. What do you think are some of the benefits of using continuous monitoring devices at your facility? What impacts do you think they have (if in current use) or would have (if not in current use) on routine care at this facility?
3. Do you have any concerns about using continuous monitoring devices? What are the challenges to using such devices at this facility? Are there any situations you think the use of monitoring devices would not be useful? If so, can you explain with an example?
Probes: Tell me about how newborns are monitored in your facility? How is this different (if at all) for sick newborns?
4. What do you think would be needed to scale up the use of continuous monitoring devices at this facility? What enablers do you think could support this process?
5. Imagine if monitoring devices were scaled up at this facility, how do you think the nurses and doctors that work here would react? How do you think caregivers (mothers, parents, guardians, etc.) would react? What about outside stakeholders and decision-makers at local, county, and national levels?

K. EarlySense InSight investigational device

Research staff shows EarlySense InSight device to HCA and explains how it works.

For peer review only

"The EarlySense InSight device is a contact-free newborn monitoring system. The system includes a sensor pad that is placed under the newborn's mattress to measure heart rate, breathing rate, motion, and sleep status. There is no physical contact between the newborn and the sensor pad. Information from the sensor pad is continuously transmitted to a monitor or tablet that can be read by hospital staff. The system has been previously tested for safety in neonates."
Allow them to touch the InSight device, the mattress pad and the cable that goes between them.

6. Could you share what you like (if anything) about this device? What do you think would be useful in the care of newborns at your healthcare facility?
7. Could you share what you dislike (if anything) about this device? What about the device do you think could create difficulties in caring for newborns at your facility?
8. Do you think this device is suitable for use in your facility? What do you think would need to happen in order to successfully use this device in your facility?
   Probes: For example, staffing availability and skill to use the device, training, complexity of the device, availability of equipment and infrastructure needed for its use, durability and maintenance of device and components, access to spare parts, protocols and guidelines for use, counselling caregivers and informational materials? What could be the benefits/drawbacks?
9. How do you think healthcare providers, like doctors and nurses, would feel about this device?
10. How do you think caregivers, such as mothers, fathers, mothers-in-law and other family members, would feel about this device?
11. How do you think other healthcare administrators and decision-makers at local, county and national levels would react to a recommendation to implement this device at this facility
12. In your opinion, how much would your facility pay for a device like this? (Circle Response)

<$5000 KSh	$5000 – $10000 KSh	$10000 – $15000 KSh
$15000 - $20000 KSh	$20000 - $25000 KSh	>$25000 KSh

Please explain

L. Sibel ANNE investigational device

Research staff shows Sibel ANNE device to HCA and explains how it works.
"The Sibel ANNE device uses non-invasive sensors to continuously measure and record a newborn's heart rate, breathing rate, level of oxygen in the blood, and skin temperature. One sensor is attached to the newborn's chest and contains a battery. The second sensor is battery-free, ultra-thin, and is applied to the newborn's hand or foot. Information from the sensors is wirelessly transmitted to a monitor or tablet that can be read by hospital staff. The system has previously been tested for safety in neonates."
Allow them to touch the ANNE chest and limb sensors, the hydrogel, and iPad display fully.

1. Could you share what you like (if anything) about this device? What do you think would be useful in the care of newborns at your healthcare facility?
2. Could you share what you dislike (if anything) about this device? What about the device do you think could create difficulties in caring for newborns at your facility?
3. Do you think this device is suitable for use in your facility? What do you think would need to happen in order to successfully use this device in your facility?
   Probes: For example, staffing availability and skill to use the device, training, complexity of the device, availability of equipment and infrastructure needed for its use, durability and maintenance of device and components, access to spare parts, protocols and guidelines for use, counselling caregivers and informational materials? What could be the benefits/drawbacks?
4. How do you think healthcare providers, like doctors and nurses, would feel about this device?
5. How do you think caregivers, such as mothers, fathers, mothers-in-law and other family members, would feel about this device?

For peer review only

6. How do you think other healthcare administrators and decision-makers at local, county and national levels would react to a recommendation to implement this device at this facility?
7. In your opinion, how much would your facility pay for a device like this? (Circle Response)

<$5000 KSh	$5000 – $10000 KSh	$10000 – $15000 KSh	
$15000 - $20000 KSh	$20000 - $25000 KSh	>$25000 KSh	

Please explain

M. Masimo RAD-97 reference device

Research staff shows Masimo Rad-97 device to HCA and explains how it works.

"The Masimo Rad-97 is a non-invasive device that measures a newborn's heart rate, breathing rate, and level of oxygen in the blood. Information is collected through a skin sensor that is applied to the newborn's hand or foot and a tube that is inserted into the newborn's nostrils. The information is then continuously transmitted to a monitor that can be read by hospital staff. The system has previously been tested for safety in neonates."

Allow them to touch the Rad-97, skin sensor and capnography tube fully.

1. Could you share what you like (if anything) about this device? What do you think would be useful in the care of newborns at your healthcare facility?
2. Could you share what you dislike (if anything) about this device? What about the device do you think could create difficulties in caring for newborns at your facility?
3. Do you think this device is suitable for use in your facility? What do you think would need to happen in order to successfully use this device in your facility?
   Probes: For example, staffing availability and skill to use the device, training, complexity of the device, availability of equipment and infrastructure needed for its use, durability and maintenance of device and components, access to spare parts, protocols and guidelines for use, counselling caregivers and informational materials? What could be benefits/drawbacks?
4. How do you think healthcare providers, like doctors and nurses, would feel about this device?
5. How do you think caregivers, such as mothers, fathers, mothers-in-law and other family members, would feel about this device?
6. How do you think other healthcare administrators and decision-makers at local, county and national levels would react to a recommendation to implement this device at this facility?
7. In your opinion, how much would your facility pay for a device like this? (Circle Response)

<$5000 KSh	$5000 – $10000 KSh	$10000 – $15000 KSh
$15000 - $20000 KSh	$20000 - $25000 KSh	>$25000 KSh

Please explain.

H. Closing

1. Taking into consideration the three monitoring devices we have talked about today, can you rank the device (if any) you think is the best, second best and third choice in your opinion? Please explain why.
2. In terms of feasibility, which device (if any) do you think would be the most appropriate device for your healthcare facility and why?
3. In terms of acceptability, which device (if any) do you think healthcare providers would like the best and why? Which device (if any) do you think caregivers would prefer and why?
4. Do you have any other comments about any of the three devices that we did not talk about?
5. Do you have any other comments about newborn monitoring devices or any other comments or concerns overall that we did not get to talk about?

"Thank you for your time and the helpful information you have provided. Your feedback, along with feedback from other people we talk to will be used to recommend solutions for better care."

For peer review only

**Interview end time:** |H|H| : |M|M| *military time*

2.3 In-Depth Interview Guide – Healthcare Provider (HCP) Direct Use

**Administrative information**	
**HCP ID number:**	**Sex:** ☐ Female ☐ Male
**Date HCP informed consent form (ICF) signed:** |D|D| - |M|M|M| - |Y|Y|Y|Y|	
**HCP ICF signed prior to any study questions?** ☐ Yes ☐ No	
**Name of research staff who explained the ICF:**	
**Does the HCP agree to be audio recorded?** ☐ Yes ☐ No<br>If Yes, was the interview audio recorded? ☐ Yes ☐ No<br>If No, why was the interview not recorded? ____	
**Name of interviewer:**	
**Date of interview:** |D|D| - |M|M|M| - |Y|Y|Y|Y|	
**Location of interview:**<br>☐ **Aga Khan University – Nairobi Hospital**<br>☐ **Pumwani Maternity Hospital**	
**Interview start time:** |H|H| : |M|M| *military time*	

<u>Instructions for qualitative research staff</u>:

- Use this document as a guide to conduct the interviews with **healthcare providers (HCP) directly using the devices**.
- Conduct the interview in the language with which the HCP feels most comfortable.
- The interview should take place in a quiet place that allows privacy.
- Please introduce each question separately. The interview must flow as a conversation. If you notice that the HCP is hesitant in answering, does not give an in-depth response, or the response is not satisfactory, please probe or ask follow-up questions, but do NOT prompt any specific answer. Several probes are suggested, and you may also ask follow-up questions that are not listed in this guide but are necessary for the complete expression of the HCP's views.
- Please **record the interview** using the audio recorder (if HCP consent is provided) and state the ETNA HCP ID number.
- All comments from the HCP should be recorded/written verbatim.
- Please cross-check the narratives written with the recorded version as a reference, and correct as necessary.
- All responses must be kept confidential. Do not discuss or share responses with anyone outside of the ETNA study team.

Script to initiate the interview

"Hello, my name is ___________ and I am a researcher with the ETNA project and we are evaluating monitoring devices for newborns. We want to hear about your experiences and learn from your thoughts and feelings. We will keep what you tell us today confidential, which means that nothing you say will be directly linked to you so please feel free to share. If you feel uncomfortable with any questions, let me know and we will skip it. Before we start, do you have any questions for me? Is it ok to begin? Thank you, I will start the audio-recording now."

For peer review only - http://bmjopen.bmj.com/site/about/guidelines.xhtml

N.	Demographic information
7.	First, we will start with some questions about yourself, what is your age?
8.	How many years of education and training have you completed and what is your highest level of education completed? What is your medical background/designation? (e.g., doctor, nurse, technician, etc.)
9.	How long have you worked as 'doctor/nurse/technician/etc.?

O.	Healthcare provider role
	4. How long have you been employed at this healthcare facility?<br>5. What is your job title and current role here at this facility? How long have you been in this role at this facility?<br>6. What are your responsibilities in this role?<br>*Probes: Please share what a typical day as a healthcare provider would be like for you.*<br>*7.* Are you involved in patient care? If yes, please explain your patient care responsibilities.

P.	Facility
5.	What are the current constraints to providing care to newborns at this healthcare facility?<br>*Probes: What makes care more difficult? What would make it easier?*
6.	How are newborns monitored at this facility? How is this different (if at all) for sick newborns?
7.	Does this facility have reliable access to electricity? When was the last electricity outage and how long do they typically last? What happens during power outages at your facility?<br>*Probes: How do power outages affect patient care? Is there a back-up power supply? If so, what is the process of using the backup power supply and are there any issues around its use (e.g., does it cover all of the equipment needed, any issues in getting permission for its use, fuel prices? Any voltage issues?*
8.	Do you have regular access to computers at this facility? If yes, do they work well?<br>*Probes: Do computers breakdown often? If yes, please describe how the computer breakdowns affect your work as a healthcare provider?*
9.	Could you describe the technologies that are being used in the delivery and newborn care wards at this facility? What are some concerns you have, or gaps in the technologies available, for maternal and newborn care at this facility?<br>*Probes: Which healthcare providers use the technologies? What technologies/brands are used? Do healthcare providers use any handheld or portable devices for maternal or newborn care (e.g., tablets or Smartphones)? If yes, please describe the technologies and their use.*

Q.	Monitoring devices
	1. What is your role with the Evaluation of Technologies for Neonates in Africa (ETNA) research study? What are your ETNA-related responsibilities?<br>**We would especially like to learn about your perspectives on continuous monitoring devices.**<br>2. Before this study, had you used continuous monitoring devices or seen them used? Tell me about your experience with continuous monitoring devices.<br>*Probes: List devices used, then discuss each device sequentially (where used, for what purpose?). How frequently have you used these types of devices? Did you find them to be useful? If yes, how so? If no, why not? What sort of training did you receive for the use of these devices?*<br>3. Apart from the devices used in the ETNA study, are continuous monitoring devices used at this healthcare facility?<br>*Probes: If yes, where in the facility? For what purpose? How frequently do you use these devices?*<br>4. What do you think are some of the benefits of using continuous monitoring devices? What impacts do you think they could have on routine care at this facility?

For peer review only

5. Do you have any concerns about using continuous monitoring devices? What are the challenges to using such devices at this facility? Are there any situations you think the use of monitoring devices would not be useful? If so, could you explain with an example?
6. What do you think would be needed to scale up the use of continuous monitoring devices at this facility? What enablers do you think could support this process?
7. Imagine if continuous monitoring devices were scaled up at this facility, how do you think the nurses and doctors that work here would react? How do you think caregivers (mothers, parents, guardians, etc.) would react?

E. EarlySense InSight investigational device

The next set of question will focus on your experiences with the EarlySense InSight device.

Usability

13. Do you think that healthcare providers in this facility could develop the skills necessary to use this continuous monitoring device? Why or why not?
14. What sort of training did you receive on this device before you began using it?
    Probes: Please describe length and method of training. Who provided training? Was training adequate? What additional training do you wish you had received? What sort of training do you think would be required for healthcare providers in this facility to use this device?
15. Which aspects of using this device were easy to learn? Which aspects were difficult?
    Probes: Did using the device become easier or more difficult over time?
16. If you now feel comfortable using the device, how long did it take you to become comfortable? If not comfortable, why not?
17. What kind of support did you receive during this period? Please explain.
    Probes: From device manufacturers, supervisors, coworkers, etc.?
18. What did you like (if anything) about this device overall? What did you dislike (if anything)? Are there any changes you would make to this device? If so, what are they?
    Probes: For example, overall device setup/interface, ease of use, etc.? What about the different features: InSIght device, mattress pad, cable, mobile application, monitor screen/display, etc.?
19. Did this device make providing care to newborns at this facility easier or more difficult? How so?
    Probes: For example, enable more care, interrupt care, etc.
20. Do you think the device would make care easier or more difficult if you could use the information collected and displayed by this device? How so?
21. Were there any questions you had about this device while you were using it? Please explain.
22. Did caregivers or other hospital staff ask you any questions about this device while you were using it? If so, what did they ask?
23. Are there situations where you think this device should not be used? If so, what are they?

Acceptability

24. How do you think other healthcare providers, like doctors and nurses, would feel about this device?
25. Based on your encounters with caregivers, such as mothers, fathers, mothers-in-law and other family members, how do you think they would feel about this device?
26. How do you think healthcare administrators and decision-makers at local, district and national levels would react to a recommendation to implement this device at this facility?
    Probes: Discuss at each level (local, district, national) sequentially. What stakeholders would influence the uptake of this technology?
27. Do you think healthcare providers would consider information collected and displayed by this device trustworthy? Why or why not?
28. Would you like to see your facility incorporate this device into newborn care? Why or why not?

Feasibility

29. Do you think this device is suitable for your facility? What would need to happen in order to integrate this device successfully at this facility? Please explain.

For peer review only

Probes: For example, staffing availability and skill to use the device, training, complexity of the device, availability of equipment and infrastructure needed for its use, durability and maintenance of device and components, access to spare parts, protocols and guidelines for use, counselling caregivers and informational materials? What could be benefits/drawbacks?

Probes: For example, ease of use during a patient visit, integration into current flow of hospital operations, acceptance by administrators, etc.?

30. Do you have any other comments about this device that we did not talk about?

F. Sibel ANNE investigational device

The next set of question will focus on your experiences with the Sibel ANNE device.

Usability

1. Do you think that healthcare providers in this facility could develop the skills necessary to use this continuous monitoring device? Why or why not?
2. What sort of training did you receive on this device before you began using it?
   Probes: Please describe length and method of training. Who provided training? Was training adequate? What additional training do you wish you had received? What sort of training do you think would be required for healthcare providers in this facility to use this device?
3. Which aspects of using this device were easy to learn? Which aspects were difficult?
   Probes: Did using the device become easier or more difficult over time?
4. If you now feel comfortable using the device, how long did it take you to become comfortable? If not comfortable, why not?
5. What kind of support did you receive during this period? Please explain.
   Probes: From device manufacturers, supervisors, coworkers, etc.?
6. What did you like (if anything) about this device overall? What did you dislike (if anything)? Are there any changes you would make to this device? If so, what are they?
   Probes: For example, overall device setup/interface, ease of use, etc.? What about the different features: chest and limb sensors, hydrogel, mobile application, iPad screen/display, etc.?
7. Did this device make providing care to newborns at this facility easier or more difficult? How so?
   Probes: For example, enable more care, interrupt care, etc.
8. Do you think the device would make care easier or more difficult if you could use the information collected and displayed by this device? How so?
9. Were there any questions you had about this device while you were using it? Please explain.
10. Did caregivers or other hospital staff ask you any questions about this device while you were using it? If so, what did they ask?
11. Are there situations where you think this device should not be used? If so, what are they?

Acceptability

12. How do you think other healthcare providers, like doctors and nurses, would feel about this device?
13. Based on your encounters with caregivers, such as mothers, fathers, mothers-in-law and other family members, how do you think they would feel about this device?
14. How do you think healthcare administrators and decision-makers at local, district and national levels would react to a recommendation to implement this device at this facility?
    Probes: Discuss at each level (local, district, national) sequentially. What stakeholders would influence the uptake of this technology?
15. Do you think healthcare providers would consider information collected and displayed by this device trustworthy? Why or why not?
16. Would you like to see your facility incorporate this device into newborn care? Why or why not?

Feasibility

17. Do you think this device is suitable within your facility? What would need to happen in order to integrate this device successfully at this facility? Please explain.

For peer review only

Probes: For example, staffing availability and skill to use the device, training, complexity of the device, availability of equipment and infrastructure needed for its use, durability and maintenance of device and components, access to spare parts, protocols and guidelines for use, counselling caregivers and informational materials? What could be benefits/drawbacks?
Probes: For example, ease of use during a patient visit, integration into current flow of hospital operations, acceptance by administrators, etc.?

18. Do you have any other comments about this device that we did not talk about?

G. Masimo RAD-97 reference device

The next set of question will focus on your experiences with the Masimo Rad-97 device.

Usability

1. Do you think that healthcare providers in this facility could develop the skills necessary to use this continuous monitoring device? Why or why not?
2. What sort of training did you receive on this device before you began using it?
   Probes: Please describe length and method of training. Who provided training? Was training adequate? What additional training do you wish you had received? What sort of training do you think would be required for healthcare providers in this facility to use this device?
3. Which aspects of using this device were easy to learn? Which aspects were difficult?
   Probes: Did using the device become easier or more difficult over time?
4. If you now feel comfortable using the device, how long did it take you to become comfortable? If not comfortable, why not?
5. What kind of support did you receive during this period? Please explain.
   Probes: From device manufacturers, supervisors, coworkers, etc.?
6. What did you like (if anything) about this device overall? What did you dislike (if anything)? Are there any changes you would make to this device? If so, what are they?
   Probes: For example, overall device setup/interface, ease of use, etc.? What about the different features: Rad-97 device, skin sensor, capnography tube, mobile application, monitor screen/display, etc.?
7. Did this device make providing care to newborns at this facility easier or more difficult? How so?
   Probes: For example, enable more care, interrupt care, etc.
8. Do you think the device would make care easier or more difficult if you could use the information collected and displayed by this device? How so?
9. Were there any questions you had about this device while you were using it? Please explain.
10. Did caregivers or other hospital staff ask you any questions about this device while you were using it? If so, what did they ask?
11. Are there situations where you think this device should not be used? If so, what are they?

Acceptability

12. How do you think other healthcare providers, like doctors and nurses, would feel about this device?
13. Based on your encounters with caregivers, such as mothers, fathers, mothers-in-law and other family members, how do you think they would feel about this device?
14. How do you think healthcare administrators and decision-makers at local, district and national levels would react to a recommendation to implement this device at this facility?
    Probes: Discuss at each level (local, district, national) sequentially. What stakeholders would influence the uptake of this technology?
15. Do you think healthcare providers would consider information collected and displayed by this device trustworthy? Why or why not?
16. Would you like to see your facility incorporate this device into newborn care? Why or why not?

Feasibility

For peer review only

17. Do you think this device is suitable within your facility? What would need to happen in order to integrate this device successfully at this facility? Please explain.
    Probes: For example, staffing availability and skill to use the device, training, complexity of the device, availability of equipment and infrastructure needed for its use, durability and maintenance of device and components, access to spare parts, protocols and guidelines for use, counselling caregivers and informational materials? What could be benefits/drawbacks?
    Probes: For example, ease of use during a patient visit, integration into current flow of hospital operations, acceptance by administrators, etc.?
18. Do you have any other comments about this device that we did not talk about?

**H. Closing**
6. Taking into consideration the three monitoring devices we have talked about today, could you rank the device (if any) you think is the best, second best and third choice in your opinion? Please explain why.<br>7. In terms of feasibility, which device (if any) do you think would be the most appropriate device for your healthcare facility and why?<br>8. In terms of acceptability, which device (if any) do you think healthcare providers would like the best and why? Which device (if any) do you think caregivers would prefer and why?<br>9. Do you have any other comments about any of the three devices that we did not talk about?<br>10. Do you have any other comments about newborn monitoring devices or any other comments or concerns overall that we did not get to talk about?
"Thank you for your time and the helpful information you have provided. Your feedback, along with feedback from other people we talk to will be used to recommend solutions for better care."
**Interview end time:** |H|H| : |M|M| *military time*

2.4 In-Depth Interview Guide – Healthcare Provider (HCP) In-Direct Use

**Administrative information**	
**HCP ID number:**	**Sex:** ☐ Female ☐ Male
**Date HCP informed consent form (ICF) signed:** |D|D| - |M|M|M| - |Y|Y|Y|Y|	
**HCP ICF signed prior to any study questions?** ☐ Yes ☐ No	
**Name of research staff who explained the ICF:**	
**Does the HCP agree to be audio recorded?** ☐ Yes ☐ No<br>If Yes, was the interview audio recorded? ☐ Yes ☐ No<br>If No, why was the interview not recorded? ______________________________	
**Name of interviewer:**	
**Date of interview:** |D|D| - |M|M|M| - |Y|Y|Y|Y|	
**Location of interview:**<br>☐ **Aga Khan University – Nairobi Hospital**	

For peer review only

☐ **Pumwani Maternity Hospital**

Interview start time: |H|H| : |M|M| *military time*

<u>Instructions for qualitative research staff</u>:

Use this document as a guide to conduct the interviews with the **healthcare providers (HCP) not directly using the devices**.

Conduct the interview in the language with which the HCP feels most comfortable.

The interview should take place in a quiet place that allows privacy.

Please introduce each question separately. The interview must flow as a conversation. If you notice that the HCP is hesitant in answering, does not give an in-depth response, or the response is not satisfactory, please probe or ask follow-up questions, but do NOT prompt any specific answer. Several probes are suggested, and you may also ask follow-up questions that are not listed in this guide but are necessary for the complete expression of the HCP's views.

Please **record the interview** using the audio recorder (if HCP consent is provided) and state the ETNA HCP ID number.

All comments from the HCP should be recorded/written verbatim.

Please cross-check the narratives written with the recorded version as a reference, and correct as necessary.

All responses must be kept confidential. Do not discuss or share responses with anyone outside of the ETNA study team.

Script to initiate the interview

"Hello, my name is ___________ and I am a researcher with the ETNA project and we are evaluating monitoring devices for newborns. We want to hear from your experiences and learn from your thoughts and feelings. We will keep what you tell us today confidential, which means that nothing you say will be directly linked to you so please feel free to share. If you feel uncomfortable with any questions, let me know and we will skip it. Before we start, do you have any questions for me? Is it ok to begin? Thank you, I will start the audio-recording now."

R. Demographic information

10. First, we will start with some questions about yourself, what is your age?
11. How many years of education and training have you completed and what is your highest level of education completed? What is your medical background/designation (e.g., doctor, nurse, technician, etc.)?
12. How long have you worked as 'doctor/nurse/technician/etc.?

S. Healthcare provider role

8. How long have you been employed at this healthcare facility?
9. What is your job title and current role here at this facility? How long have you been in this role at this facility?
10. What are your responsibilities in this role?
    Probes: Please share what a typical day as a healthcare provider would be like for you.
11. Are you involved in patient care? If yes, please explain your patient care responsibilities.

T. Facility

For peer review only

10. What are the current constraints to providing care to newborns at this healthcare facility?
    Probes: What makes care more difficult? What would make it easier?
11. How are newborns monitored at this facility? How is this different (if at all) for sick newborns?
12. Does this facility have reliable access to electricity? When was the last electricity outage and how long do they typically last? What happens during power outages at your facility?
    Probes: How do power outages affect patient care? Is there a back-up power supply? If so, what is the process of using the backup power supply and are there any issues around its use (e.g., does it cover all of the equipment needed, any issues in getting permission for its use, fuel prices? Any voltage issues?)
13. Do you have regular access to computers at this facility? If yes, do they work well?
    Probes: Do computers breakdown often? If yes, please describe how the computer breakdowns affect your work as a healthcare provider?
14. Could you describe the technologies that are being used in the delivery and newborn care wards at this facility? What are some concerns you have, or gaps in the technologies available, for maternal and newborn care at this facility?
    Probes: Which healthcare providers use the technologies? What technologies/brands are used? Do healthcare providers use any handheld or portable devices for maternal or newborn care (e.g.,, tablets or Smartphones)? If yes, please describe the technologies and their use.

U. Monitoring devices

1. Are you familiar with the Evaluation of Technologies for Neonates in Africa (ETNA) research study? Are you involved with the study?
   Probes: Are you familiar with the purpose of the study and/or study procedures? Have you previously spoken with any study staff?

We would especially like to learn about your perspectives on continuous monitoring devices.

2. Are continuous monitoring devices used in any capacity at this healthcare facility?
   Probes: If yes, where in the facility? For what purpose? How frequently do you use these devices?
3. Tell me about your experience with continuous monitoring devices. Have you used devices yourself or seen them used?
   Probes: List devices used, then discuss each device sequentially (where, for what purpose?). How frequently have you used these types of devices? Did you find them to be useful? If yes, how so? If no, why not? What sort of training did you receive for the use of these devices?
4. What do you think are some of the benefits (if any) of using continuous monitoring devices? What impacts do you think they could have on routine care at this facility?
5. Do you have any concerns about using continuous monitoring devices? What are the challenges (if any) to using such devices at this facility? Are there any situations you think the use of monitoring devices would not be useful? If so, could you explain with an example?
6. What do you think would be needed to scale up the use of continuous monitoring devices at this facility? What enablers do you think could support this process?
7. Imagine if continuous monitoring devices were scaled up at this facility, how do you think the nurses and doctors that work here would react? How do you think caregivers (mothers, parents, guardians, etc.) would react?

V. EarlySense InSight investigational device

31. Are you familiar with the EarlySense InSight device?
32. Have you ever seen this device before? Have you used it or seen it being used?

Research staff shows EarlySense InSight to HCP and explains how it works.

"The EarlySense InSight is a contact-free physiological monitoring system. The system includes a sensor pad that is placed under the neonate's mattress and can measure pulse, respiratory rate, motion, and sleep status. There is no direct physical contact between the neonate and the sensor pad.

For peer review only - http://bmjopen.bmj.com/site/about/guidelines.xhtml

Information from the sensor pad is continuously transmitted to a central display that can be read by hospital staff."

Allow them to touch the InSight device, the mattress pad and the cable that goes between them.

Usability

33. Do you think that healthcare providers in this facility have the skills necessary to use this device? Why or why not?
34. What sort of training do you think would be required for providers in this facility to be able to use this device?
    Probes: What do think the appropriate length of time and method of delivery of training?
35. Which aspects of using this device do you think would be easy to learn? Which aspects would be difficult?
    Probes: What barriers do you anticipate?
36. How do you think using this device would affect providing care to newborns at this facility?
    Probes: Would using this device make provision of care easier or more difficult?
37. What kind of questions do you think caregivers or other hospital staff would have about this device?
38. Are there any changes you would make to this device? If so, what are they?
39. Are there situations where you think this device should not be used? If so, what are they?

Acceptability

40. What do you like (if anything) about this device overall? What do you dislike (if anything)?
    Probes: For example, overall device setup/interface, InSIght device, mattress pad, cable, mobile application, monitor screen/display, etc.?
41. How do you think other healthcare providers, like doctors and nurses, would feel about this device?
42. How do you think caregivers, such as mothers, fathers, mothers-in-law and other family members, would feel about this device?
43. How do you think healthcare administrators and decision-makers at local, district and national levels would react to a recommendation to implement this device at this facility?
    Probes: Discuss at each level (local, district, national) sequentially. What stakeholders would influence the uptake of this technology?
44. Would you consider information collected and displayed by this device trsutworthy? Why or why not?
45. Do you think this device could be useful for monitoring newborns at this facility? Why or why not?
46. Would you like to see your facility incorporate this device into newborn care? Why or why not?

Feasibility

47. Do you think this device is suitable for your facility? What would need to happen in order to integrate this device successfully at this facility? Please explain.
    Probes: For example, staffing availability and skill to use the device, training, complexity of the device, availability of equipment and infrastructure needed for its use, durability and maintenance of device and components, access to spare parts, protocols and guidelines for use, counselling caregivers and informational materials? What could be benefits/drawbacks?
    Probes: For example, ease of use during a patient visit, integration into current flow of hospital operations, acceptance by administrators, etc.?
48. Do you have any other comments about this device that we did not talk about?

W. Sibel ANNE investigational device

1. Are you familiar with the Sibel ANNE device?
2. Have you ever seen this device before? Have you used it/seen it being used?

Research staff show Sibel Advanced Neonatal Epidermal (ANNE) system to HCP and explains how it works:

For peer review only

"The Sibel Advanced Neonatal Epidermal System, referred to as the ANNE system, uses non-invasive sensors to continuously measure and record a neonate's pulse, respiratory rate, level of oxygen in the blood or SpO2, and skin temperature. One sensor is attached to the neonate's chest and the second sensor is applied to the neonate's foot. Information from the sensors is wirelessly transmitted to a monitor or tablet that can be read by hospital staff."

Allow them to touch the ANNE chest and limb sensors, hydrogel, and tablet display fully.

Usability

3. Do you think that healthcare providers in this facility have the skills necessary to use this device? Why or why not?
4. What sort of training do you think would be required for providers in this facility to be able to use this device?
   Probes: What do think the appropriate length of time and method of delivery of training?
5. Which aspects of using this device do you think would be easy to learn? Which aspects would be difficult?
   Probes: What barriers do you anticipate?
6. How do you think using this device would affect providing care to newborns at this facility?
   Probes: Would using this device make provision of care easier or more difficult?
7. What kind of questions do you think caregivers or other hospital staff would have about this device?
8. Are there any changes you would make to this device? If so, what are they?
9. Are there situations where you think this device should not be used? If so, what are they?

Acceptability

10. What do you like (if anything) about this device overall? What do you dislike (if anything)?
    Probes: For example, overall device setup/interface, ANNE chest and limb sensor, hydrogel, tablet, mobile application, monitor screen/display, etc.?
11. How do you think other healthcare providers, like doctors and nurses, would feel about this device?
12. How do you think caregivers, such as mothers, fathers, mothers-in-law and other family members, would feel about this device?
13. How do you think healthcare administrators and decision-makers at local, district and national levels would react to a recommendation to implement this device at this facility?
    Probes: Discuss at each level (local, district, national) sequentially. What stakeholders would influence the uptake of this technology?
14. Would you trust the information collected and displayed by this device? Why or why not?
15. Do you think this device could be useful for monitoring newborns at this facility? Why or why not?
16. Would you like to see your facility incorporate this device into newborn care? Why or why not?

Feasibility

17. Do you think this device is suitable for your facility? What would need to happen in order to integrate this device successfully at this facility? Please explain.
    Probes: For example, staffing availability and skill to use the device, training, complexity of the device, availability of equipment and infrastructure needed for its use, durability and maintenance of device and components, access to spare parts, protocols and guidelines for use, counselling caregivers and informational materials? What could be benefits/drawbacks?
    Probes: For example, ease of use during a patient visit, integration into current flow of hospital operations, acceptance by administrators, etc.?
18. Do you have any other comments about this device that we did not talk about?

**X. Masimo RAD-97 reference device**
1. Are you familiar with the Masimo RAD-97 device?

For peer review only

2. Have you ever seen this device before? Have you used it/seen it being used?

Research staff shows Masimo Rad-97 device to HCP and explains how it works.

"The Masimo Rad-97 is a non-invasive device that measures a neonate's pulse, respiratory rate, and level of oxygen in the blood or SpO2, in a hospital setting. Information is collected through a skin sensor that is applied to the newborn's hand or foot and a cannula tube that is inserted into the neonate's nostrils. The information is then continuously transmitted to a monitor that can be read by hospital staff."

Allow them to touch the Rad-97, skin sensor and capnography tube fully.

Usability

3. Do you think that healthcare providers in this facility have the skills necessary to use this device? Why or why not?
4. What sort of training do you think would be required for providers in this facility to be able to use this device?
   Probes: What do think the appropriate length of time and method of delivery of training?
5. Which aspects of using this device do you think would be easy to learn? Which aspects would be difficult?
   Probes: What barriers do you anticipate?
6. How do you think using this device would affect providing care to newborns at this facility?
   Probes: Would using this device make provision of care easier or more difficult?
7. What kind of questions do you think caregivers or other hospital staff would have about this device?
8. Are there any changes you would make to this device? If so, what are they?
9. Are there situations where you think this device should not be used? If so, what are they?

Acceptability

10. What do you like (if anything) about this device overall? What do you dislike (if anything)?
    Probes: For example, overall device setup/interface, Rad-97 device, skin sensor, capnography tube, mobile application, monitor screen/display, etc.?
11. How do you think other healthcare providers, like doctors and nurses, would feel about this device?
12. How do you think caregivers, such as mothers, fathers, mothers-in-law and other family members, would feel about this device?
13. How do you think healthcare administrators and decision-makers at local, district and national levels would react to a recommendation to implement this device at this facility?
    Probes: Discuss at each level (local, district, national) sequentially. What stakeholders would influence the uptake of this technology?
14. Would you trust the information collected and displayed by this device? Why or why not?
15. Do you think this device could be useful for monitoring newborns at this facility? Why or why not?
16. Would you like to see your facility incorporate this device into newborn care? Why or why not?

Feasibility

17. Do you think this device is suitable for your facility? What would need to happen in order to integrate this device successfully at this facility? Please explain.
    Probes: For example, staffing availability and skill to use the device, training, complexity of the device, availability of equipment and infrastructure needed for its use, durability and maintenance of device and components, access to spare parts, protocols and guidelines for use, counselling caregivers and informational materials? What could be benefits/drawbacks?
    Probes: For example, ease of use during a patient visit, integration into current flow of hospital operations, acceptance by administrators, etc.?
18. Do you have any other comments about this device that we did not talk about?

H. Closing

For peer review only

11. Taking into consideration the monitoring devices we have talked about today, could you rank the device (if any) you think is the best, second best and third choice in your opinion? Please explain why.<br>12. In terms of feasibility, which device (if any) do you think would be the most appropriate for your healthcare facility and why?<br>13. In terms of acceptability, which device (if any) do you think healthcare providers would like the best and why? Which device (if any) do you think caregivers would prefer and why?<br>14. Do you have any other comments about any of the three devices that we did not talk about?<br>15. Do you have any other comments about newborn monitoring devices or any other comments or concerns overall that we did not get to talk about?
"Thank you for your time and the helpful information you have provided. Your feedback, along with feedback from other people we talk to will be used to recommend solutions for better care."
**Interview end time:** |H|H| : |M|M| *military time*

For peer review only

S3 File Healthcare Provider (HCP) Direct Observation Guide

**A. Administrative information**
**HCP ID number:**
**Date HCP informed consent form (ICF) signed:** |D|D| - |M|M| - |Y|Y|Y|Y|
**HCP ICF signed prior to any observation?** ☐ Yes ☐ No<br>If no, please do not make any observations until the ICF has been completed.
**Name of observer:**
**Neonate ID number:**
**Date of observation:** |D|D| - |M|M| - |Y|Y|Y|Y|
**Location of observation:**<br>☐ **Aga Khan University Hospital – Nairobi**<br>☐ **Pumwani Maternity Hospital**
**Observation start time:** |H|H| : |M|M| *military time*<br>*(Time HCP began device preparation)*
**There are three different phases that can be observed and reported in the fields below:**<br>1. Device preparation and initial application: observing HCP prepare and place device on neonate.<br>2. Ongoing device monitoring and troubleshooting: observing HCP perform regular checks of device placement on neonate (and repositioning if necessary) and data quality, including troubleshooting.<br>3. Device disconnection, removal, and cleaning: observing HCP remove device from neonate, clean and store.<br>**Instructions for qualitative research staff:**<br>Use this document as a guide to conduct observations of one HCP during one or more of the phases described above. Indicate in checklist below which phase(s) were included in this observation session.<br>**Use a new form for each HCP**. Two different HCP should not be included on the same form. **Use a new form for each neonate and for each observation session day**. Two different neonates should not be included on the same form. Two different observation session days should not be included on the same form. Multiple observations of the same neonate by the same HCP on the same day can be included on the same form.

Record observations. All observations must be kept confidential. Do not discuss or share observations with anyone outside of the ETNA study team.

For peer review only

**B. Phase(s) observed during this session on the same neonate on the same day (check all that apply)**
☐ Device preparation and initial application<br>☐ Ongoing device monitoring and troubleshooting<br>☐ Device disconnection, removal and cleaning

**C. Which devices did the HCP use during today's observation?**		
EarlySense InSight investigational device	☐ Yes	☐ No
Sibel ANNE investigational device	☐ Yes	☐ No
Masimo Rad-97 reference device	☐ Yes	☐ No

**D. PHASE 1: Device initial application**
**EarlySense InSight investigational device** *(if device not used, skip to next section)*
Application start time: |H|H| : |M|M| military time<br>Application end time: |H|H| : |M|M| military time<br><br>☐ Did not complete device preparation and initial application
Please check those steps that you observed. Comments and observations can be made below.<br><br>***Preparation***<br>☐ Remove neonate from bed/bassinet<br>☐ Place pad under neonate's mattress<br>☐ Gently place neonate back on bed/bassinet with chest above middle of pad<br>☐ Attach pad cord to InSight device<br>☐ Confirm InSight device is seen on EarlySense laptop/CDS<br>***Admission***<br>☐ Correct name of admitting nurse selected in EarlySense laptop/CDS<br>☐ Enter PTID into EarlySense laptop/CDS admit patient screen in MRN (ID) box
Please provide comments if HCP <u>did not complete</u> device preparation and initial application.<br>Also, if HCP was not able to complete steps correctly, what did they do instead?

For peer review only

Did you observe HCP have any challenges or difficulties preparing and/or applying device? What were the problems and how were they resolved?
Did HCP require any assistance when preparing and/or applying device?<br>□ Yes □ No<br>If yes, who assisted HCP?<br>If yes, what kind of assistance was required?
Did you observe any risky situations where mistakes could potentially happen, such as times when HCP almost made a mistake? If yes, please explain.
Did HCP make any other comments to you or their colleagues related to preparing and/or applying device? If yes, record comments verbatim and provide context as necessary.
**Sibel ANNE investigational device** *(if device not used, skip to next section)*
Application start time: |H|H| : |M|M| military time<br>Application end time: |H|H| : |M|M| military time<br>□ Did not complete device preparation and initial application
Please check those steps that you observed. Comments and observations can be made below.<br>***Preparation for data collection***<br>□ ANNE Connect application opened immediately after Sibel iPad unlocked<br>□ Participant ID entered to start data collection session<br>□ Correct chest and limb sensors selected from within ANNE Connect app<br>***Application of ANNE chest sensor***<br>□ Open new hydrogel package and apply hydrogel adhesive to chest sensor or neonate's chest, with gentle but firm pressure<br>□ Place chest sensor on the torso of the neonate and apply gentle but firm pressure to secure sensor to hydrogel adhesive<br>***Application of ANNE limb sensor***<br>□ Insert limb sensor into Velcro strap holes Apply LED to bottom of neonate's foot<br>□ Apply limb sensor on neonate's foot with LED to bottom of neonate's foot Check that photodiode is aligned with LED<br>□ Confirm proper limb sensor placement by checking ANNE Connect application to verify that an error message is not displayed<br>***Confirmation of data collection***<br>□ Correctly close ANNE Connect application (without disconnecting within Connect app)<br>□ Open ANNE Stream application to check quality of vital signs signals
Please provide comments if HCP <u>did not complete</u> device preparation and initial application.<br>Also, if HCP was not able to complete steps correctly and **in order**, what did they do instead?
Did you observe HCP have any challenges or difficulties preparing and/or applying device? What were the problems and how were they resolved?
Did HCP require any assistance when preparing and/or applying device?<br>□ Yes □ No<br>If yes, who assisted HCP?<br>If yes, what kind of assistance was required?
Did you observe any risky situations where mistakes could potentially happen, such as times when HCP almost made a mistake? If yes, please explain.

For peer review only

Did HCP make any other comments to you or their colleagues related to preparing and/or applying device? If yes, record comments verbatim and provide context as necessary.

Masimo Rad-97 reference device *(if device not used, skip to next section)*

Application start time: |H|H| : |M|M| military time
Application end time: |H|H| : |M|M| military time

□ Did not complete device preparation and initial application

Please check those steps that you observed. Comments and observations can be made below.

- □ Power on Rad-97 device
- □ Plug in a RD Rainbow SET Series Patient Cable to Patient Cable Port on front of Rad-97 device
- □ Plug in new, unused NomoLine Infant Cannula to round NomoLine Capnography Input Connector on front of Rad-97 device
- □ Attach RD SET Series SpO2 Disposable Sensor to Patient Cable
- □ Apply skin sensor to hand or foot
- □ Ensure sensor wrapped securely but not too tightly and ensure correct alignment of light and detector
- □ Cover sensor to avoid interference from external light sources (as needed)
- □ Insert capnography tubing into nostrils, ensuring that the cannula is not obstructed from collecting CO_2
- □ Secure cannula in place using neonate-safe adhesive as needed
- □ Ensure good quality (square) capnography waveform and high signal quality (perfusion index or PI) on Rad-97 monitor

Please provide comments if HCP did not complete device preparation and initial application.
Also, if HCP was not able to complete steps correctly, what did they do instead?

Did you observe HCP have any challenges or difficulties preparing and/or applying device? What were the problems and how were they resolved?

Did HCP require any assistance when preparing and/or applying device?
□ Yes □ No
If yes, who assisted HCP?
If yes, what kind of assistance was required?

Did you observe any risky situations where mistakes could potentially happen, such as times when HCP almost made a mistake? If yes, please explain.

Did HCP make any other comments to you or their colleagues related to preparing and/or applying device? If yes, record comments verbatim and provide context as necessary.

E. PHASE 2: Ongoing device monitoring and troubleshooting

EarlySense InSight investigational device *(if device not used, skip to next section)*

Did you observe HCP have any challenges or difficulties with device monitoring and/or troubleshooting? What were the problems and how were they resolved?

Did HCP do any troubleshooting during ongoing monitoring? If so, please describe what the issues were, how the HCP addressed them and an estimate for how long it took.

Issue	Solution	Start Time	End Time

Did HCP require any assistance when monitoring the EarlySense InSIght investigational device?
□ Yes □ No
If yes, who assisted HCP?
If yes, what kind of assistance was required?

Did you observe any risky situations where mistakes could potentially happen, such as times when HCP almost made a mistake? If yes, please explain.

Did HCP make any other comments to you or their colleagues related to device monitoring and/or troubleshooting? If yes, record comments verbatim and provide context as necessary.

Sibel ANNE investigational device *(if device not used, skip to next section)*

Did HCP complete the following steps correctly? Please check those steps that you observed. Comments and observations can be made below.

- ☐ Open ANNE Stream application to check quality of vital signs waveforms (lines) and perfusion index (PI).
- ☐ Take corrective measures to address signal quality issues (if needed)?

If signal quality issues needed to be addressed, what corrective measures did they take?

Did you observe HCP have any challenges or difficulties with device monitoring and/or troubleshooting? What were the problems and how were they resolved?

Did HCP do any troubleshooting during ongoing monitoring? If so, please describe what the issues were, how the HCP addressed them and an estimate for how long it took.

Issue	Solution	Start Time	End Time

Did HCP require any assistance when monitoring the Sibel ANNE investigational device?
□ Yes □ No
If yes, who assisted HCP?
If yes, what kind of assistance was required?

Did you observe any risky situations where mistakes could potentially happen, such as times when HCP almost made a mistake? If yes, please explain.

Did HCP make any other comments to you or their colleagues related to device monitoring and/or troubleshooting? If yes, record comments verbatim and provide context as necessary.

Masimo Rad-97 reference device *(if device not used, skip to next section)*

Did HCP complete the following steps correctly? Please check those steps that you observed. Comments and observations can be made below.

- ☐ Confirm adequate signal quality (PI) for skin sensor
- ☐ Confirm adequate signal quality (waveform) for capnography tube

If signal quality issues needed to be addressed, what corrective measures did they take?

- ☐ Confirm placement of skin sensor
- ☐ Confirm placement of cannula
- ☐ Confirm connection of Patient Cable to Patient Cable port
- ☐ Confirm connection of Capnography Input Connector
- ☐ Other ____

Did you observe HCP have any challenges or difficulties with device monitoring and/or troubleshooting? What were the problems and how were they resolved?

Did HCP do any troubleshooting during ongoing monitoring? If so, please describe what the issues were, how the HCP addressed them and an estimate for how long it took.

Issue	Solution	Start Time	End Time

Did HCP require any assistance when monitoring the Masimo Rad-97 reference device?
□ Yes □ No
If yes, who assisted HCP?
If yes, what kind of assistance was required?

Did you observe any risky situations where mistakes could potentially happen, such as times when HCP almost made a mistake? If yes, please explain.

Did HCP make any other comments to you or their colleagues related to device monitoring and/or troubleshooting? If yes, record comments verbatim and provide context as necessary.

F. PHASE 3: Device disconnection, removal, and cleaning

EarlySense InSight investigational device *(if device not used, skip to next section)*

Did HCP discharge neonate from EarlySense laptop/CDS correctly? If not, what did HCP do instead?

Did you observe HCP have any challenges or difficulties with device disconnection, removal, and/or cleaning? What were the problems and how were they resolved?

Did the HCP require any assistance with device disconnection, removal, and/or cleaning?
□ Yes □ No
If yes, who assisted the HCP?
If yes, what kind of assistance was required?

Did you observe any risky situations where mistakes could potentially happen, such as times when HCP almost made a mistake? If yes, please explain.

Did HCP make any other comments to you or their colleagues related to device disconnection, removal, and/or cleaning? If yes, record comments verbatim and provide context as necessary.

Sibel ANNE investigational device *(if device not used, skip to next section)*

Please check those steps that you observed. Comments and observations can be made below.

- ☐ Disconnect chest and limb sensors from Devices tab of ANNE Stream application
- ☐ Close ANNE Stream application
- ☐ Close ANNE Sync application by swiping up on application
- ☐ Re-open ANNE Connect application
- ☐ Disconnect limb sensor first
- ☐ Disconnect chest sensor
- ☐ End session by selecting "End Session" button from ANNE Connect application
- ☐ Sanitize hands according to study site infection control policy
- ☐ Remove chest sensor by gently pulling off, away from the neonate, on one corner
- ☐ Gently remove any residual adhesive using a saline cleaning wipe
- ☐ Unfasten Velcro button from strap and remove limb sensor

☐ Dispose of used Velcro strap<br>☐ Clean chest and limb sensors, wipe both sides<br>☐ Dispose used cleaning wipe
If HCP was not able to complete steps correctly and <u>in order</u>, what did they do instead?
Did you observe HCP have any challenges or difficulties with device disconnection, removal, and/or cleaning? What were the problems and how were they resolved?
Did the HCP require any assistance with device disconnection, removal, and/or cleaning?<br>□ Yes □ No<br>If yes, who assisted the HCP?<br>If yes, what kind of assistance was required?
Did you observe any risky situations where mistakes could potentially happen, such as times when HCP almost made a mistake? If yes, please explain.
Did HCP make any other comments to you or their colleagues related to device disconnection, removal, and/or cleaning? If yes, record comments verbatim and provide context as necessary.
**Masimo Rad-97 reference device** *(if device not used, skip to next section)*
Did HCP complete the following steps correctly? Please check those steps that you observed. Comments and observations can be made below.<br>☐ Remove adhesive (if present) and capnography tube gently from neonate<br>☐ Carefully remove skin sensor from neonate<br>☐ Dispose of single use capnography tube and disposable skin sensor<br>☐ Unplug capnography tube and patient cable from Rad-97<br>☐ Unplug skin sensor from patient cable<br>☐ Turn off Rad-97
If HCP was not able to complete steps correctly, what did they do instead?
Did you observe HCP have any challenges or difficulties with device disconnection, removal, and/or cleaning? What were the problems and how were they resolved?
Did the HCP require any assistance with device disconnection, removal, and/or cleaning?<br>□ Yes □ No<br>If yes, who assisted the HCP?<br>If yes, what kind of assistance was required?
Did you observe any risky situations where mistakes could potentially happen, such as times when HCP almost made a mistake? If yes, please explain.
Did HCP make any other comments to you or their colleagues related to device disconnection, removal, and/or cleaning? If yes, record comments verbatim and provide context as necessary.

Please note below any further comments that may have not already been covered in above sections. In particular, if you have any observations comparing the HCP's use of the different devices, if applicable.

S4 File Coding tree

Nodes	Sub-nodes	Description
A. Social-demographics information	1. Age	Age of participant
	2. Job title	Job title and current role at the facility
	3. Employment duration at facility	Duration of employment at the healthcare facility
	4. Work experience	Duration worked as a physician, nurse, technician, etc.
	5. Education	Years of education and training completed, highest level of education completed, medical background/designation (e.g., physician, nurse, technician, etc.)
	6. Healthcare provider role	Responsibilities, patient care responsibilities
B. Health system factors	1. Current constraints	Description of the current constraints to providing care to newborns at the healthcare facility. Factors that make care more difficult or easy
	2. Monitoring of newborns at the facility	Methods of newborn monitoring at the facility. How it is different (if at all) for sick newborns
	3. Access to electricity	Description of whether the facility have reliable access to electricity. The last electricity outage and how long do they typically last. What happens during power outages at the facility. How do power outages affect patient care. A back-up power supply. The process of using the backup power supply and any issues around its use (e.g., does it cover all of the equipment needed, any issues in getting permission for its use, fuel prices. Any voltage issues.
	4. Access to computers	Description of whether they have regular access to computers at this facility Whether they work well. Computers breakdown. Ways in which the computer breakdowns affect ones work as a healthcare provider
	5. Technologies used in delivery and newborn unit	Description of the technologies that are being used in the delivery and newborn care wards at this facility. Concerns or gaps in the technologies available, for maternal and newborn care at the facility. Type of healthcare providers who use the technologies. Technologies/ brands used. Whether the healthcare providers use

For peer review only

C. Monitoring devices	1. Familiarity with role and responsibilities with ETNA	Role with the ETNA research study and any ETNA-related responsibilities
	2. Use of continuous monitoring devices	Use of continuous monitoring devices or seen them used. Experience with continuous monitoring devices. List of devices used, how frequently one has used the types of devices. usefulness. Training received for the use of the devices.
	3. Experience with continuous monitoring devices	Description of whether continuous monitoring devices apart from the ETNA devices are used at the healthcare facility. If so, where in the facility, their purpose and frequency of use.
	4. Benefits	Benefits of using continuous monitoring devices and impacts on routine care at the facility
	5. Concerns	Any concerns about using continuous monitoring devices. Challenges to using such devices at this facility. Any situations in which the use of monitoring devices would not be useful.
	6. Need for scale up	What would be needed to scale up the use of continuous monitoring devices at the facility. Enablers that could support the process.
	7. Reaction on use of monitoring devices	Reaction of the nurses and physicians if use of continuous monitoring devices were scaled up at the facility. Reaction of caregivers (mothers, parents, guardians, etc.)
	8. Training	Any mention around training and training needs for monitoring devices in general
D. EarlySense investigational device	A. Familiarity with device	Previous experience with the device
	B. Usability	Discussions around device usability, likes and dislikes about the device, situations where the device should not be used
	C. Acceptability	Feelings of healthcare providers, administrators and caregivers about the device, whether they trusted results and if device should be incorporated
	D. Feasibility	Discussions whether the device would be suitable within their health setting
E. Sibel investigational	A. Familiarity with device	Previous experience with the device

For peer review only

device	B. Usability	Discussions around device usability, likes and dislikes about the device, situations where the device should not be used
	C. Acceptability	Feelings of healthcare providers, administrators and caregivers about the device, whether they trusted results and if device should be incorporated
	D. Feasibility	Discussions whether the device would be suitable within their health setting
F. Masimo RAD-97 reference device	A. Familiarity with device	Previous experience with the device
	B. Usability	Discussions around device usability, likes and dislikes about the device, situations where the device should not be used
	C. Acceptability	Feelings of healthcare providers, administrators and caregivers about the device, whether they trusted results and if device should be incorporated
	D. Feasibility	Discussions whether the device would be suitable within their health setting
G. Closing	A. Rank device	Rank of the device as the best, second best and third choice
	B. Feasibility – most appropriate device	In terms of feasibility, device (if any) that would be the most appropriate device for the healthcare facility
	C. Acceptability – most preferred device	In terms of acceptability, device (if any) that the healthcare providers and caregiver would like the best.
	D. Other comments about the devices	Any other comments about three ETNA study devices in general
	E. Any other comments about newborn monitoring devices or any other comments or concerns	Any other comments about newborn monitoring devices or any other comments or concerns

For peer review only

COREQ Checklist

Consolidated criteria for reporting qualitative studies (COREQ): 32-item checklist

No. Item	Guide questions/description	Reported on Page #
Domain 1: Research team and reflexivity		
Personal Characteristics		
1. Inter viewer/facilitator	Which author/s conducted the interview or focus group?	Pg 6
2. Credentials	What were the researcher's credentials? E.g. PhD, MD	Pg 6
3. Occupation	What was their occupation at the time of the study?	Pg 6
4. Gender	Was the researcher male or female?	Pg 6
5. Experience and training	What experience or training did the researcher have?	Pg 6
Relationship with participants		
6. Relationship established	Was a relationship established prior to study commencement?	Pg 6
7. Participant knowledge of the interviewer	What did the participants know about the researcher? e.g. personal goals, reasons for doing the research	Pg 6
8. Interviewer characteristics	What characteristics were reported about the inter viewer/facilitator? e.g. Bias, assumptions, reasons and interests in the research topic	Pg 6
Domain 2: study design		
Theoretical framework		
9. Methodological orientation and Theory	What methodological orientation was stated to underpin the study? e.g.	Pg 5

For peer review only

	grounded theory, discourse analysis, ethnography, phenomenology, content analysis	
Participant selection		
10. Sampling	How were participants selected? e.g. purposive, convenience, consecutive, snowball	Pg 5
11. Method of approach	How were participants approached? e.g. face-to-face, telephone, mail, email	Pg 6
12. Sample size	How many participants were in the study?	Pg 7
13. Non-participation	How many people refused to participate or dropped out? Reasons?	Pg 7
Setting		
14. Setting of data collection	Where was the data collected? e.g. home, clinic, workplace	Pg 6
15. Presence of non-participants	Was anyone else present besides the participants and researchers?	Pg 6
16. Description of sample	What are the important characteristics of the sample? e.g. demographic data, date	Pg 7
Data collection		
17. Interview guide	Were questions, prompts, guides provided by the authors? Was it pilot tested?	Pg 6; supplementary file 1 and 2
18. Repeat interviews	Were repeat interviews carried out? If yes, how many?	Pg 6
19. Audio/visual recording	Did the research use audio or visual recording to collect the data?	Pg 6
20. Field notes	Were field notes made during and/or after the interview or focus group?	Pg 6
21. Duration	What was the duration of the interviews or focus group?	Pg 6
22. Data saturation	Was data saturation discussed?	Pg 6

For peer review only

23. Transcripts returned	Were transcripts returned to participants for comment and/or correction?	Pg 6
Domain 3: analysis and findings		
Data analysis		
24. Number of data coders	How many data coders coded the data?	Pg 6-7
25. Description of the coding tree	Did authors provide a description of the coding tree?	Pg 7, supplementary file 3
26. Derivation of themes	Were themes identified in advance or derived from the data?	Pg 6-7
27. Software	What software, if applicable, was used to manage the data?	Pg 6-7
28. Participant checking	Did participants provide feedback on the findings?	Pg 6-7
Reporting		
29. Quotations presented	Were participant quotations presented to illustrate the themes/findings? Was each quotation identified? e.g. participant number	Pg 7-14
30. Data and findings consistent	Was there consistency between the data presented and the findings?	Pg 7-14
31. Clarity of major themes	Were major themes clearly presented in the findings?	Pg 7-14
32. Clarity of minor themes	Is there a description of diverse cases or discussion of minor themes?	Pg 7-14

For peer review only

BMJ Open

A qualitative study exploring the feasibility, usability, and acceptability of neonatal continuous monitoring technologies at a public tertiary hospital in Nairobi, Kenya

Journal:	*BMJ Open*
Manuscript ID	bmjopen-2021-053486.R1
Article Type:	Original research
Date Submitted by the Author:	21-Oct-2021
Complete List of Authors:	Kinshella, Mai-Lei Woo; The University of British Columbia, Department of Obstetrics and Gynecology; BC Children's Hospital, Centre for International Child Health<br>Naanyu, Violet; Moi University, School of Arts and Sciences<br>Chomba, Dorothy; The Aga Khan University - Kenya, Department of Pediatrics<br>Waiyego, Mary ; Pumwani Maternity Hospital<br>Rigg, Jessica; BC Children's Hospital, Centre for International Child Health; The University of British Columbia, Department of Anesthesiology<br>Coleman, Jesse; Evaluation of Technologies for Neonates in Africa<br>Hwang, Bella; BC Children's Hospital, Centre for International Child Health<br>Ansermino, J. Mark; BC Children's Hospital, Centre for International Child Health; The University of British Columbia, Department of Anesthesiology<br>Macharia, William; The Aga Khan University - Kenya, Department of Pediatrics<br>Ginsburg, Amy Sarah; University of Washington, Clinical Trial Center
**Primary Subject Heading**:	Global health
Secondary Subject Heading:	Paediatrics, Qualitative research
Keywords:	QUALITATIVE RESEARCH, Neonatal intensive & critical care < INTENSIVE & CRITICAL CARE, International health services < HEALTH SERVICES ADMINISTRATION & MANAGEMENT

SCHOLARONE™
Manuscripts

I, the Submitting Author has the right to grant and does grant on behalf of all authors of the Work (as defined in the below author licence), an exclusive licence and/or a non-exclusive licence for contributions from authors who are: i) UK Crown employees; ii) where BMJ has agreed a CC-BY licence shall apply, and/or iii) in accordance with the terms applicable for US Federal Government officers or employees acting as part of their official duties; on a worldwide, perpetual, irrevocable, royalty-free basis to BMJ Publishing Group Ltd ("BMJ") its licensees and where the relevant Journal is co-owned by BMJ to the co-owners of the Journal, to publish the Work in this journal and any other BMJ products and to exploit all rights, as set out in our licence.

The Submitting Author accepts and understands that any supply made under these terms is made by BMJ to the Submitting Author unless you are acting as an employee on behalf of your employer or a postgraduate student of an affiliated institution which is paying any applicable article publishing charge ("APC") for Open Access articles. Where the Submitting Author wishes to make the Work available on an Open Access basis (and intends to pay the relevant APC), the terms of reuse of such Open Access shall be governed by a Creative Commons licence – details of these licences and which Creative Commons licence will apply to this Work are set out in our licence referred to above.

Other than as permitted in any relevant BMJ Author's Self Archiving Policies, I confirm this Work has not been accepted for publication elsewhere, is not being considered for publication elsewhere and does not duplicate material already published. I confirm all authors consent to publication of this Work and authorise the granting of this licence.

For peer review only

A qualitative study exploring the feasibility, usability, and acceptability of neonatal continuous monitoring technologies at a public tertiary hospital in Nairobi, Kenya

Mai-Lei Woo Kinshella[1,2*], Violet Naanyu[3], Dorothy Chomba[4], Mary Waiyego[5], Jessica Rigg[2,6], Jesse Coleman[7], Bella Hwang[2], J. Mark Ansermino[2,6], William M. Macharia[4], Amy Sarah Ginsburg[8]

[1] Department of Obstetrics and Gynecology, British Columbia Children's and Women's Hospital and The University of British Columbia, Vancouver, British Columbia, Canada

[2] Centre for International Child Health, BC Children's Hospital, Vancouver, British Columbia, Canada

[3] School of Arts and Sciences, Moi University, Eldoret, Kenya

[4] Department of Pediatrics, Aga Khan University, Nairobi, Kenya

[5] Pumwani Maternity Hospital, Nairobi, Kenya

[6] Department of Anesthesiology, The University of British Columbia, Vancouver, British Columbia, Canada

[7] Evaluation of Technologies for Neonates in Africa, United States

[8] Clinical Trial Center, University of Washington, Seattle, Washington, United States

*Corresponding Author:
Mai-Lei Woo Kinshella
Department of Obstetrics & Gynaecology
University of British Columbia
950 West 28th Avenue
Vancouver, BC, Canada, V5Z 4H4
Email: maggie.kinshella@cw.bc.ca

Word count: 5498

Keywords: continuous monitoring technologies; neonate; Africa; medical technology design; user perspectives; qualitative research

For peer review only - http://bmjopen.bmj.com/site/about/guidelines.xhtml

ABSTRACT

Objective: To assess the feasibility, usability, and acceptability of two non-invasive, multiparameter, continuous physiological monitoring (MCPM) technologies for use in neonates within a resource-constrained healthcare setting in sub-Saharan Africa.

Design: A qualitative study using in-depth interviews and direct observations to describe healthcare professional and caregiver perspectives and experiences with investigational MCPM technologies from EarlySense and Sibel compared to selected reference technologies.

Setting: Pumwani Maternity Hospital is a public, high-volume, tertiary hospital in Nairobi, Kenya.

Participants: In-depth interviews were conducted with five healthcare administrators, 12 healthcare providers, and 10 caregivers. Direct observations were made of healthcare providers using the technologies on 12 neonates overall.

Results: Design factors like non-invasiveness, portability, ease-of-use, and ability to measure multiple vital signs concurrently emerged as key themes supporting the usability and acceptability of the investigational technologies. However, respondents also reported feasibility challenges to implementation, including overcrowding in the neonatal unit, lack of reliable access to electricity and computers, and concerns about cost and maintenance needs. To improve acceptability, respondents highlighted the need for adequate staffing to appropriately engage caregivers and dispel misconceptions about the technologies.

Conclusion: Study participants were positive about the usefulness of the investigational technologies to strengthen clinical care quality and identification of at-risk neonates for better access to timely interventions. These technologies have the potential to improve equity of access to appropriate healthcare services and neonatal outcomes in sub-Saharan African healthcare facilities. However, health system strengthening is also critical to support sustainable uptake of technologies into routine care.

Trial registration: NCT03920761

For peer review only - http://bmjopen.bmj.com/site/about/guidelines.xhtml

STRENGTHS AND LIMITATIONS OF THIS STUDY

- We interviewed healthcare administrators, providers, and caregivers to understand the feasibility, usability, and acceptability of investigational technologies from multiple perspectives.
- The purposeful sampling design elicited a wide range of perspectives although these cannot be used to determine representative frequency of themes.
- The triangulation of direct observations with in-depth interviews helped to strengthen reliability of findings.
- The current study is compared with findings from a previous study conducted at a private healthcare facility in Nairobi, Kenya with the same technologies and methodology to illuminate different implementation factors between private and public tertiary hospitals.

For peer review only

For peer review only - http://bmjopen.bmj.com/site/about/guidelines.xhtml

BACKGROUND

Leading causes of neonatal deaths, including 35% due to preterm birth complications, 24% due to birth asphyxia and trauma, and 15% due to neonatal sepsis and infections, are preventable with quality facility-based care (1,2). However, effective implementation of evidence-based neonatal interventions may require monitoring of vital signs and time-sensitive clinical follow-up, which may be compromised in resource-constrained healthcare settings (3,4). Locally appropriate technologies to support early detection of physiologically unstable neonates requiring timely intervention have the potential to improve quality of care and neonatal health outcomes (5).

The Evaluation of Technologies for Neonates in Africa (ETNA) platform aims to boost development and optimization of promising neonatal medical technologies to be used in resource-constrained healthcare facilities. Understanding user perspectives in the intended setting is critical to medical technology design, development, deployment, and eventual uptake and acceptance. However, the feasibility, appropriateness, and acceptability of novel technologies for improving maternal and neonatal health are not often adequately investigated, thereby compromising implementation efforts (6). The ETNA platform previously conducted a qualitative evaluation of two novel, non-invasive, multiparameter, continuous physiological monitoring (MCPM) technologies developed by EarlySense and Sibel at Aga Khan University Hospital (AKUH), a private, tertiary hospital in Nairobi, Kenya where MCPM technologies were already used in neonatal intensive care (Ginsburg 2021). By contrast, Pumwani Maternity Hospital (PMH) is a public, high-volume maternity hospital in Nairobi where MCPM technologies are not routinely used. In the current study, we assessed the feasibility, usability, and acceptability of the same MCPM technologies at PMH to better understand the technologies' use for neonates within a resource-constrained healthcare setting in sub-Saharan Africa.

METHODS

Study design and setting

Comprised of in-depth interviews (IDIs) and direct observations, this descriptive qualitative study elicited perspectives and experiences of healthcare professionals and caregivers around

For peer review only - http://bmjopen.bmj.com/site/about/guidelines.xhtml

MCPM technology feasibility, usability, and acceptability. We evaluated the accuracy, reliability, and performance of novel MCPM technologies in comparison with verified reference technologies (Figure 1). Frequently used in hospitals worldwide, the Masimo Rad-97 reference technology was selected based on its capability for high resolution data collection and neonatal capnometry and pulse oximetry. We present the findings based on the "Consolidated criteria for reporting qualitative research" (COREQ) (7,8). The current study utilized the following definitions (9,10):

- Feasibility involved systemic factors required for implementation of MCPM technologies, such as hospital infrastructure, operational capacities, and functional capacities of available healthcare providers (HCP);
- Usability involved design factors that influenced HCP user experience, such as ease and efficiency of use, frequency of errors, memorability to a casual user, and user satisfaction; and
- Acceptability involved factors that influenced the willingness of healthcare administrators (HCA), HCP, and caregivers to use the technology.

PMH is a public, tertiary referral hospital serving Nairobi, Kenya and is the largest referral maternity hospital in sub-Saharan Africa with an average of 50-100 deliveries a day. Neonates in good health accompany their mothers to the postnatal ward while neonates with health complications are admitted to the neonatal unit, a large hall separated into 11 cubicles representing different diagnoses and care requirements. Neonates in more critical health conditions are placed in cubicles closest to the nursing station, while stable neonates awaiting discharge are moved to cubicles on the other side of the hall. Neonates commonly share cots and incubators with up to four neonates in each. The neonatal unit is typically staffed by three nurses and three clinical officers or physicians during the morning shift, and then two nurses and one clinical officer or physician during the afternoon and night shifts. The study moved between the different cubicles within the neonatal unit and employed two dedicated study nurses to support the study. Caregiver visitation times are restricted to every three hours for the mothers to breastfeed and care for the neonates.

Recruitment and data collection

A purposefully drawn study sample included HCA, direct and indirect HCP, and caregivers of

For peer review only - http://bmjopen.bmj.com/site/about/guidelines.xhtml

neonates enrolled in ETNA. Direct HCP consisted of ETNA study nurses who were direct users of the MCPM technologies (HCP-D) and indirect HCP included hospital physicians, nurses, and clinical staff involved in neonatal care but who did not actively use the investigational or reference MCPM technologies (HCP-I). Multiple MCPM technologies were used with each neonate enrolled in ETNA during their hospital stay. A sample size of five HCA, 12 HCP, and 10 caregivers was estimated to reach data saturation covering a wide range of perspectives from the healthcare staffing positions and caregivers available.

Study recruitment was publicized using flyers and potential participants were approached in person by a member of the qualitative study team, who introduced themselves and the ETNA study. To minimize bias, a Kenyan research consultant (VN, PhD in sociology, female) and two trained female research assistants (diplomas in health sciences) who did not know participants prior to the study activities were hired to conduct the IDIs with the enrolled qualitative study participants and the direct observations of the ETNA study nurses.

IDIs with HCA, HCP, and caregivers and direct observations of HCP-D were conducted between November 23 and December 1, 2020 following semi-structured IDI and structured observation guides. To investigate the accuracy, reliability, and performance of the technologies, IDIs included questions regarding reactions to technology use, consideration of result trustworthiness, advantages and concerns about each technology, local health system constraints, and suitability within their facility (Supplementary file 1). While the focus of the study is to understand the feasibility, usability, and acceptability of the investigational technologies, the same questions were asked about all three technologies to allow for contextualization and comparison. Additionally, direct observations of HCP-D using the technologies covered three different phases of usage for each of the MCPM technologies: 1) technology preparation and initial application; 2) ongoing technology monitoring and troubleshooting; and 3) technology disconnection, removal, and cleaning (Supplementary file 2). Data collection guides were developed for the ETNA qualitative study and piloted by the Kenyan data collection team during training to refine questions. After obtaining written informed consent, IDIs were conducted in person in a quiet, private place within PMH in English or Kiswahili, the major local languages in Kenya, depending on study participant preference. IDIs took between 18 to 78 minutes to conduct with an average length of 46.6 minutes. Written informed consent was obtained from HCP-D for

For peer review only - http://bmjopen.bmj.com/site/about/guidelines.xhtml

observations IDIs were audio-recorded with permission, field notes recorded during data collection, and no repeat IDIs were conducted.

Data analysis

IDIs were transcribed verbatim and translated into English. Transcripts were uploaded into NVivo 12 software (QSR International, Melbourne, Australia) for qualitative analysis following a thematic approach. Thematic analysis involved becoming familiar with the data, generating initial codes collating identified codes into themes, and describing themes using illustrative quotes (11). A coding framework (supplementary file 3) was developed deductively from study objectives to cover feasibility, usability, and acceptability as well as inductively from emergent themes by the ETNA study team (MWK, VN, DC, JR, JC, WMM, ASG). VN conducted the primary coding with review by MWK.

Data confidentiality was ensured through limiting access of study materials to authorized personnel, de-identifying participants using codes, and aggregating demographic features. Ethics approvals were obtained from Western Institutional Review Board 20 191 102 (Puyallup, Washington, USA), and the Aga Khan University Nairobi Research Ethics Committee 2019/REC-02 (v2) (Nairobi, Kenya).

Patient and public involvement

Neither patients nor public were involved in the design or conduct of the study.

RESULTS

Direct observations of HCP-D using the technologies on 12 neonates were made and IDIs conducted with 27 participants, including five HCA, 10 HCP-I (six nurses, two clinical officers, and two physicians), two HCP-D (two study nurses,), and 10 caregivers. No potential participants declined to participate. Interviewed healthcare professionals were female except for one male clinician, and ranged in age from 24 to 58 (average 36.2) years. With a median of 5 (range <1 to 35) years of work experience in the medical field, approximately half (8 of 17) of the healthcare professionals held diplomas or certificates as their highest level of formal education. Four healthcare professionals were pursuing a first degree or completed an undergraduate degree, while three held master degrees and two had medical degrees. Interviewed

For peer review only - http://bmjopen.bmj.com/site/about/guidelines.xhtml

caregivers were female ranging in age from 19 to 28 (average 22.3) years. A majority reported that this was their first child (6 of 10 caregivers, range 1 to 3 children). Eight caregivers had secondary-level education while two had primary-level education. Most (8 of 10) caregivers reported they were unemployed or a housewife, and two caregivers shared that they were involved in informal, small-scale business. Reported occupations of husbands and partners included mason, mechanic, electrician, watchman, businessman, marketing, and driver.

Key themes reported regarding technology feasibility included the number of neonates needing monitoring, reliable access to electricity and computers, and cost and maintenance implications of the MCPM technologies. Ease and efficiency of use, non-invasiveness, and portability were critical features highlighted for usability. Supporting improved monitoring capacities, concerns about radiation and electrical currents, and a need for caregiver engagement were central themes noted for the acceptability of the MCPM technologies.

Feasibility

<u>Numbers of neonates to monitor</u>

A major challenge at PMH was overcrowding, resulting in the common practice of multiple neonates within a single cot. As a HCA shared, *"…we are admitting so many babies but our capacity is low…the capacity of the unit is small as compared to the neonates we receive and that is why you find there are two-three-four babies in one-unit bed." (HCA, 1).*

HCA and HCP posited that overcrowding impacted the feasibility of scaling up individual MCPM technologies for neonates, particularly the EarlySense technology which is placed under the mattress. A study nurse said, *"We've not used [the EarlySense technology] where babies are sharing the baby cot. …we don't know of its efficiency when there's more than one [baby]…" (HCP-D, 1).* A HCA said that because the EarlySense technology *"can only take one [neonate], so it means for us we would have to prioritize really who we have to monitor so that we give them their space" (HCA, 3).* The EarlySense technology was designed for each neonate to be in an individual cot but healthcare professionals at PMH shared that this may reduce the number of neonates that could be admitted given the current practice of sharing cots.

The Sibel technology may better accommodate sharing cots as one HCA highlighted, "*sharing incubators, [the Sibel technology] is comfortable to use. I like that it is compact..." (HCA, 4).*

For peer review only - http://bmjopen.bmj.com/site/about/guidelines.xhtml

However, overcrowding still had implications for service delivery as different neonates would need to be carefully identified and their readings easily distinguishable from one another. As a clinical officer said, A "*challenge would be telling specifically this is for this baby while you have 20 babies on this [Sibel technology]. They will need to be sure that this belongs to this baby in this room. They will need to have codes for the specific baby…" (HCP-I, 9).*

Reliable access to electricity and computers

While a back-up generator was available at PMH, HCA and HCP reported that the generators were not always functional and frequently required repairs. Electrical outages could lead to delays in using technologies that required uninterrupted electricity supply, "*If there is power failure and a generator is faulty, we end up not doing what we need until electricity is back" (HCP-D, 2).*

Unreliable electricity had direct implications for the EarlySense technology, which was connected to wall power. As one nurse said, "*I saw [the EarlySense technology] is using power. So, if possible, can we have the one without the power? So that if there is no electricity we can still use it" (HCP-I, 1).* The Sibel technology used a rechargeable battery, but HCP said that ensuring the technology was fully charged when needed and charging between electrical outages would be a challenge in a busy neonatal unit. For example, a nurse said, "*… unlike other devices which you just connect to the (wall) socket and they are ready to use, [the Sibel technology has] to be prepared... So, charging them and making sure they are ready for use is a challenge for a big hospital like Pumwani" (HCP-D, 2).*

Additionally, both investigational technologies relied on the use of external screens and computers, which would require investments in equipment, spacing, and electrical infrastructure, and training for staff to use along with the current manual documentation systems. As a nurse said, "*There's no regular access to computer. There's only one, in in-charge office and… everything else is manual" (HCP-D, 1).*

Cost and maintenance

Cost and maintenance implications of the MCPM technologies were also highlighted by HCA and HCP as critical factors influencing the feasibility of potential scale-up. As a public hospital, HCA shared that PMH followed the government procurement process, and while there were a

For peer review only - http://bmjopen.bmj.com/site/about/guidelines.xhtml

procurement and budget committee and a health management board at PMH that took into account what HCP needed in their department, the medical superintendent had to approve the purchase and the Kenya Medical Supplies Authority (KEMSA) did most of the purchasing. Consequently, HCA said that a lack of funds at PMH to purchase equipment is a challenge. HCA shared that PMH was often reliant on donors and partners to fill in the gaps, "*not having funds for the equipment is a big issue because money from the county or NMS (Nairobi Metropolitan Services) is not available to us, and we have to look for donors and partners who are able to procure the equipment for us" (HCA, 4).* In addition to the initial costs of purchasing the technology, there would be additional costs around maintenance. A HCA said, "*…we have to think through how we are going to maintain this servicing. So there is a cost to it beyond the buying the purchase (HCA, 3).* Some wondered if replacement parts and the training of local biomedical engineers to service and repair the EarlySense and Sibel technologies were available in the country. Taken together with funding challenges for their initial purchase, ongoing maintenance could limit sustainable scale-up into routine care as an ETNA study nurse observed, "*I have seen sometimes maybe… because of poor maintenance…it's not effective for as long as it should have been" (HCP-D, 1).*

Usability and acceptability

Direct observations of HCP-D using the MCPM technologies within the PMH neonatal unit supported usability with appropriate availability of training and support. Similar to the Masimo reference technology, application of the EarlySense and Sibel technologies to a neonate each took on average five minutes and the HCP-D were observed to not face any difficulties with preparation, initial application, monitoring, disconnection, or cleaning. No use errors where mistakes could potentially happen were observed with either investigational technology. There was one observation with each of the investigational technologies where a HCP-D required assistance from another study nurse to help calm an irritable neonate, which interfered with technology readings (EarlySense) or application (Sibel).

Ease and efficiency in use

Design factors shared by HCA and HCP that impacted user experience included that the MCPM technologies appeared easy to use and clean. Speaking of the EarlySense technology, a HCA said, "*Looks easy to clean. That is a big issue for us because we need to observe high hygiene*

For peer review only - http://bmjopen.bmj.com/site/about/guidelines.xhtml

standards" (HCA, 4). An ETNA study nurse who used the technologies noted, "*What I liked about [the EarlySense technology] is that it's easy to place. It's quite straightforward…" (HCP-D, 1)*. A HCA said, *"[The Sibel technology] looks easy to use because you are just attaching to the extremity and the trunk" (HCA, 3).* The investigational technologies were described as easy to use for someone without extensive training.

Additionally, the MCPM technologies were described as being able to efficiently collect multiple vital signs within a single device. A clinical officer said of the EarlySense technology, "*you will be able to collect most crucial data… So you get a lot of data using a short time period" (HCP-I, 4).* Of the Sibel technology, a nurse observed, "…*It is taking four vitals at the same time, whereas if it is manual, I would have four gadgets…[such as] stethoscope, thermometers… Now that small gadget I just place it on the chest…it is giving me all that and it is fast and continuous…"(HCP-I, 3).* An ETNA study nurse said, *"it (Sibel) covers a lot of vital signs measurements, and yeah, and almost as equivalent in functionality as the cardiac monitor" (HCP-D, 1).*

The potential for the investigational technologies to increase efficiency in monitoring was highlighted to potentially extend clinical care capacity and reduce HCP workload, which supported acceptability among healthcare professionals and caregivers. HCA and HCP emphasized the challenges of maintaining regular monitoring in busy neonatal units where the number of HCP were few in comparison to the number of neonates under their care. Speaking about the EarlySense technology, a nurse said, "*This machine…is helping to ease the workload. Instead of placing one person to check on this baby and the other baby—one person can assess and monitor very many babies at a time because [the EarlySense technology] is doing all that work for him….[HCP] will be positive about it" (HCP-I, 3).* A HCA noted that the Sibel technology will be acceptable within their healthcare facility because "*I can leave the baby on something that monitors them and have a central display screen about the patients' vitals in real time. Then the nurses will not be as stretched taking the vitals on every single baby when they are very few" (HCA, 4).* Caregivers also shared that the investigational technologies would be acceptable to them because the technologies improved monitoring and clinical follow-up of their neonates.

<u>Non-invasive but concerns about radiation and electrical currents</u>

For peer review only - http://bmjopen.bmj.com/site/about/guidelines.xhtml

Additionally, the non-invasive design of the two investigational technologies was described by HCA, HCP, and caregivers to support user satisfaction because the MCPM technologies did not appear to cause neonate discomfort. For example, a caregiver said of the EarlySense technology, "*He will just sleep normally; it won't affect him, but all these [vital signs] shall be recorded so I think it will be comfortable for him" (CG, 4).* An HCA noted, *"when I put [the EarlySense technology under] the mattress, it won't be inconvenient to the baby" (HCA, 3).* Similarly, another caregiver said of the Sibel technology, "*I like it because the baby is comfortable when being placed on, he is not crying, I just feel he is fine" (CG, 3).* An HCA observed, *"…[the Sibel technology are] such light gadgets …they are not causing any undue pressure to the baby, so they should be acceptable [to caregivers]" (HCA, 3)*. In particular, respondents highlighted that the investigational technologies had no (EarlySense) or fewer (Sibel) attachments. For example, an HCA said of the Sibel technology, "*What I like about it is … it doesn't have wires. Wires bring complications" (HCA, 2).*

However, respondents shared that concerns about radiation and electrical currents with wireless and Bluetooth technologies may reduce acceptability, particularly among caregivers. A caregiver asked of the EarlySense technology, *"What I want to know is maybe, does it have side effects because if it doesn't touch him, how does it monitor? Maybe [the EarlySense technology] can cause radiation, cancer or something?" (CG, 4).* In reference to the Sibel technology, a caregiver also spoke of "*the fear of transfer of dangerous waves to the body of the baby"* (CG, 7). An ETNA study nurse shared, "*[The caregivers] are concerned about the transfer of data from the Sibel device, both limb and chest units, to the iPad… The main concern is [that] Bluetooth uses radioactive material, so how sure are we that these devices will not harm the baby?" (HCP-D, 2).* An HCA described that counselling may be required to fully explain the MCPM technology and dispel misconceptions, *"…our population may wonder is there some electrical current going through my baby's body… but if we take our time and explain, they wouldn't have a problem" (HCA, 3).*

HCA, HCP, and caregivers emphasized the need for caregiver counselling and engagement to support acceptability. Different caregivers may also react differently to the use of MCPM technologies, so understanding caregiver perceptions was essential for appropriate engagement. For example, a physician said, "*There are those who worry extremely because when they see the*

For peer review only - http://bmjopen.bmj.com/site/about/guidelines.xhtml

gadgets on the baby, they get worried. The other groups of patients think that, the more gadgets there are, the better. What is important is to explain to the mother and understand their perception of what they are seeing" (HCP-I, 7). A nurse said, "*I think they will like [the Sibel technology] but still, it depends on how we communicate about it…I believe with good communication, they will definitely embrace it" (HCP-I, 8).*

Movement and portability

HCA and HCP shared that movement and portability features could both support and/or hinder operating the technology for its intended purpose. Both of the investigational technologies were portable and could be moved throughout the neonatal unit to where they were needed. An HCA said, *"I like the fact that [the EarlySense technology] is a portable sized tool" (HCA, 3).* However, while the EarlySense technology was portable, continuous monitoring was interrupted if the neonate was not calm or taken off the mattress for breastfeeding or other care needs such as diaper changing or kangaroo mother care. A nurse said of the EarlySense technology, "*…it might present a challenge when it is feeding time…. [Mothers] will just come and take the baby off…" (HCP-I, 9).* An ETNA study nurse said, "*[The EarlySense technology] should also not be used during resuscitation whereby there is a lot of movement during chest compressions. This device should be used only for calm babies…" (HCP-D, 2).*

The portable Sibel technology allowed for neonate movement, as one nurse said, "*It is light, easily portable, and even with the movement of the baby, it won't fall off. [The Sibel technology] won't give us inaccurate results even with the movement of the baby" (HCP-I, 8).* However, because of its small size and highly portable design characteristic, some worried that the Sibel technology may be misplaced or stolen. An ETNA study nurse said, "*They are very small devices which can get lost easily" (HCP-D, 2).* Additionally, a HCA said, "[the Sibel technology is] *so portable and can be stolen."(HCA, 2).*

Comparison of the investigational and reference technologies

Like with the investigational technologies, a major challenge of feasibility for the Masimo Rad-97 reference technology was overcrowding in the PMH neonatal unit. HCA and HCP highlighted that the stand-alone Masimo Rad-97 unit required even more space than the investigational technologies, which compromised feasibility at their facility. A nurse said, *"We really get*

For peer review only - http://bmjopen.bmj.com/site/about/guidelines.xhtml

packed here … I feel [the Masimo Rad-97 technology] will give us more headaches because it needs more space… it will mean that every room, maybe we may have two to three tables to put it on …that will be a bit hectic" (HCP-I, 8).

In contrast to the non-invasive design of the investigational technologies, HCA, HCP, and caregivers highlighted that the Masimo Rad-97 technology had many wires and tubes. More attachments to the neonate was perceived to compromise neonate comfort and reduce accuracy because neonate movement may dislodge a connection, "*Those many tubes, for babies who are a little bit active, the jumpiness of the babies can alter one or two things [and the] readings can be bad" (HCA, 1).* The Masimo Rad-97 technology's nasal cannula tubing and wires were perceived by study respondents as invasive, interfering with the neonate's movement and potentially increasing the risk of infection. For example, a HCA said, *"All foreign objects should be treated as infection routes and I am not comfortable with that" (HCA, 4).* The increased number of connections also intensified the anticipated training necessary to use the Masimo Rad-97 technology properly. For example, a nurse said, "*It has a lot of connections and tubing. If somebody is not very careful in the training, and you miss in connecting that machine, you might miss the results…" (HCP-I, 3).*

In addition to usability concerns, there were also acceptability concerns with caregivers. The Masimo Rad-97 technology capnography feature was especially concerning for mothers and their families as the capnography feature was associated with oxygen therapy and worsening neonate health conditions. An ETNA study nurse said, "*It gives the picture of oxygen. Everyone knows when my baby is on oxygen, s/he is very sick…The capnography doesn't seem necessary especially for babies who are not on oxygen because everyone's speculations at first would think you're administering oxygen" (HCP-D, 1).* Echoing the ETNA study nurse's statement, a caregiver said*, "I thought it was oxygen. He [the father] would panic…" (CG, 2).* Another caregiver said, "*Especially the pipe that goes to the nose. I would not want my child to be using it… It makes you think that the child is in a very bad state" (CG, 5).*

However, while the Masimo Rad-97 technology capnography feature reduced acceptability among caregivers, its familiarity in the neonatal unit may increase acceptability among some HCP. For example, a nurse said, "*if it's just something to insert on the nose, which is something we are familiar with, so that one can be easy..." (HCP-I, 5).* An ETNA study nurse said, "*It's*

For peer review only - http://bmjopen.bmj.com/site/about/guidelines.xhtml

familiar. It's not a new device on the ground, so it's familiar to me and to most HCP" (HCP-D, 1). Of the three technologies, 7 of 10 caregivers rated EarlySense as the most preferable. There was more diversity of responses among health professionals but overall, the Sibel technology was most frequently favorably rated. Seven of 15 HCP who responded to the question rated the Sibel technology as their top choice among the three technologies.

DISCUSSION

Design factors like non-invasiveness, portability, ease of use, and ability to measure multiple vital signs concurrently increased efficiency of care and supported the usability and acceptability of the investigational technologies in neonates in this resource-constrained setting. Our study of two investigational neonatal MCPM technologies within a resource-constrained, high-volume maternity hospital in sub-Saharan Africa highlighted how locally appropriate technologies can support improved neonatal care by expanding HCP capacity for monitoring and increased efficiency to quickly respond to emerging complications. Consequently, MCPM technologies can play a valuable role in improving quality of neonatal care as well as access, as more at-risk neonates are able to be identified and prioritized for intensive care. Yet, thoughtful user-friendly design factors cannot overcome basic infrastructural gaps, the need for adequate and trained HCP staffing to appropriately engage caregivers, or negate the need for regular technology service and support. Feasibility challenges of overcrowding and lack of reliable electricity, and caregiver acceptability challenges such as mistrust of wireless features (investigational technologies) or fear of capnography (reference Masimo Rad-97 technology), had implementation implications across all of the technologies within the study.

Currently, there are two reviews available of wearable continuous monitoring sensors for neonates, but these only compiled existing products and their key features (12,13). Acceptability and implementation factors were not explored (12,13). The NASSS (non-adoption, abandonment, scale-up, spread, and sustainability) framework posits that increasingly, complexity across seven domains (health condition, technology, value, adopters, organizational capacity, wider system context, and embedding/adaption over time) contributes to the non-adoption of novel health technologies (14). Addressing the first three domains, MCPM technologies are standard in the care of vulnerable neonates in high-resource health settings and study participants in our low-resource health setting valued their importance for improving

For peer review only

quality of care and expressed appreciation for user-friendly design features. However, acceptability and systemic factors within their organizational and infrastructural context emerged as critical domains impacting capacity for scale-up, spread, and sustainability. Our study helps to fill the current gap in understanding these domains for MCPM technologies for neonates in resource-limited settings where they are not yet routinely implemented.

In comparison to the qualitative evaluation of the investigational technologies at AKUH (Ginsburg 2021), a private, tertiary hospital in Nairobi, Kenya, there were a number of similar usability and acceptability themes. Potential harmful side effects from wireless connections and mistrust of novel technologies were voiced as concerns largely by caregivers at both hospitals. Similarly, the fears regarding the novel technologies appeared to be alleviated among some caregivers with adequate HCP explanation. The concerns around electrical fields appeared to cross socio-economic groups in Kenya as almost all of the caregivers interviewed at AKUH had university education and professional employment, compared to secondary education and lack of employment outside of the home for the majority of caregivers interviewed at PMH. Similar design features were highlighted by respondents from both PMH and AKUH to support usability of the investigational technologies, including their ease of use and ability to measure multiple vital signs as well as concerns about EarlySense technology monitoring disruptions when neonates were restless or off the mattress. Trained HCP at both hospitals were observed to effectively use the investigational technologies without difficulties.

Additionally, caregivers at both hospitals disliked the nasal capnography feature of the Masimo Rad-97 reference technology, which was associated with neonate discomfort and fears around oxygen therapy. Both AKUH and PMH groups mentioned that associations with oxygen therapy made the situation seem more dire, as if the neonate was critically ill. Caregiver anxiety around nasal oxygen and tubing also have been reported with other neonatal interventions such as bubble continuous positive airway pressure in Malawi where oxygen therapies were associated with severe illness (15). HCP counselling was helpful to alleviate caregiver concerns in both healthcare settings.

However, the context at AKUH was different than at PMH. AKUH had a ratio of three neonates to a nurse, reliable back-up electrical systems, a maintenance team on staff, and were less reliant on donor and partner support to purchase new equipment. Consequently, equipment costs,

For peer review only - http://bmjopen.bmj.com/site/about/guidelines.xhtml

electrical outages, technology malfunction, and maintenance were not emphasized as feasibility concerns at AKUH. By contrast, all of these issues were voiced as serious concerns among PMH study respondents. Overcrowding, unreliable electricity, lack of access to computers, and short staffing emerged as critical challenges to the feasibility of both the investigational and reference MCPM technologies at PMH. The identification of the general level of infrastructure and human resources are considered to be important in the development of technologies intended for use in low- and middle-income countries (LMICs) (5). The experience at PMH may be reflective of feasibility constraints in other large public hospitals in sub-Saharan Africa where adequate human, equipment, and infrastructural resources have been identified as limiting factors in the implementation of newborn health innovations (16,17). The qualitative evaluations of the investigational MCPM technologies at two urban tertiary hospitals in Nairobi, Kenya also highlighted that differences between LMICs healthcare settings may be just as important as those between high-income countries and LMICs. In particular, findings from our ETNA qualitative study support existing literature on the dramatically different hospital infrastructure and human resources between private and public hospitals in Kenya (18), which has implications for the feasibility of effective scale-up of neonatal technologies.

A limitation of the study included that only two respondents had direct experience with the investigational and reference technologies; the HCP-I and HCA interviewed did not. Though we did not find major differences in themes reported between direct and indirect users, there is a possibility that the HCP-I interviewed may shift responses given some direct experience with the technologies. Additionally, the study was cross-sectional, which captures findings within a specific point in time. The qualitative study at PMH was conducted during the COVID-19 pandemic and a healthcare worker strike in Kenya, which may have impacted findings. Furthermore, the qualitative approach was exploratory to identify themes but the purposeful sampling design was limited in its ability to quantify their representative frequency. However, conducting IDIs with caregivers, HCP, and HCA allowed an expanded understanding of feasibility, usability, and acceptability from a wide range of perspectives. The triangulation of direct observations with IDIs helped to strengthen reliability of findings, and the comparison with qualitative research recently conducted with a similar methodology and the same technologies in another healthcare setting in Nairobi, Kenya helped to deepen understanding of contextual factors.

For peer review only - http://bmjopen.bmj.com/site/about/guidelines.xhtml

CONCLUSIONS

MCPM technologies are an essential part of strengthening access to and quality of hospital-based neonatal care. In moving from the need to assess multiple vital signs individually and manually, MCPM technologies have the potential to enable ongoing multiparameter clinical monitoring and improve efficiency in care centrally monitored by HCP to ultimately improve health outcomes and save lives. This has implications for overburdened clinical staff attempting to provide high-quality neonatal care in resource-constrained healthcare settings. Identification of more at-risk neonates through the use of MCPM technologies also helps to improve access to the care they may require. Overall, study participants were positive about the usability of the investigational MCPM technologies but highlighted implementation challenges that require further consideration. New, innovative technologies need to be implemented within enabling environments. While thoughtful, user-friendly design factors can support usability, technology on its own cannot overcome feasibility challenges of basic infrastructural gaps and the continued need for adequate and trained staffing to effectively engage caregivers and support quality neonatal care. Innovative MCPM technologies have the potential to significantly improve neonatal care in sub-Saharan African healthcare facilities, but health system strengthening is also critical to support their sustainable uptake into routine care.

FOOTNOTES

Acknowledgements: The authors are grateful to the health professionals and caregivers for sharing their thoughts and experiences with us and the local hospital and community for their support. We would also like to thank Marianne Vidler for her insights on study design and the data collection tools as well as the research team who collected and managed the data without whom this study would not have been possible.

Contributors: ASG, JMA, and WMM conceptualized the ETNA platform. ASG supervised this qualitative study and designed the data collection instruments with critical input and support from VN, JR, JC, BH, and WMM. Among the authors, VN, DC, MW, and WMM were responsible for data collection and local project administration. MWK and VN performed the

For peer review only - http://bmjopen.bmj.com/site/about/guidelines.xhtml

analyses. MWK and ASG wrote the first draft of the manuscript. All authors provided feedback and review of the manuscript.

Funding: This work is supported by grants from the Bill & Melinda Gates Foundation (BMGF) (OPP1203136) and the Save the Children Innovation Fund. Following BMGF input into trial design, funders had no role in data collection, analysis, or interpretation, or writing of the report.

Conflicts of interest: None declared.

Ethics approval: Ethics approvals were obtained from Western Institutional Review Board 20 191 102 (Puyallup, Washington, USA), and the Aga Khan University Nairobi Research Ethics Committee 2019/REC-02 (v2) (Nairobi, Kenya).

Data sharing statement: De-identified data are available on request.

For peer review only

REFERENCES

1. Strong KL, Pedersen J, White Johansson E, Cao B, Diaz T, Guthold R, et al. Patterns and trends in causes of child and adolescent mortality 2000–2016: setting the scene for child health redesign. BMJ Glob Heal. 2021 Mar 17;6(3):e004760.

2. Lawn JE, Kinney M V, Belizan JM, Mason EM, McDougall L, Larson J, et al. Born too soon: accelerating actions for prevention and care of 15 million newborns born too soon. Reprod Health. 2013;10 Suppl 1(Suppl 1):S6.

3. Kinshella M-LW, Salimu S, Chiwaya B, Chikoti F, Chirambo L, Mwaungulu E, et al. "So sometimes, it looks like it's a neglected ward": Health worker perspectives on implementing kangaroo mother care in southern Malawi. Gurgel RQ, editor. PLoS One. 2020 Dec 17;15(12):e0243770.

4. Nyondo-Mipando AL, Kinshella MLW, Bohne C, Suwedi-Kapesa LC, Salimu S, Banda M, et al. Barriers and enablers of implementing bubble Continuous Positive Airway Pressure (CPAP): Perspectives of health professionals in Malawi. Ameh CA, editor. PLoS One. 2020 Feb 13;15(2):e0228915.

5. Maynard KR, Causey L, Kawaza K, Dube Q, Lufesi N, Maria Oden Z, et al. New technologies for essential newborn care in under-resourced areas: what is needed and how to deliver it. Paediatr Int Child Health. 2015 Aug 8;35(3):192–205.

6. Lunze K, Higgins-Steele A, Simen-Kapeu A, Vesel L, Kim J, Dickson K. Innovative approaches for improving maternal and newborn health - A landscape analysis. BMC Pregnancy Childbirth. 2015 Dec 17;15(1):1–19.

7. Tong A, Sainsbury P, Craig J. Consolidated criteria for reporting qualitative research (COREQ): a 32-item checklist for interviews and focus groups. Int J Qual Heal Care. 2007 Sep 16;19(6):349–57.

8. Ginsburg AS, Nkwopara E, MacHaria W, Ochieng R, Waiyego M, Zhou G, et al. Evaluation of non-invasive continuous physiological monitoring devices for neonates in Nairobi, Kenya: A research protocol. BMJ Open. 2020 Apr 12;10(4):35184.

9. Ginsburg AS, Tawiah Agyemang C, Ambler G, Delarosa J, Brunette W, Levari S, et al. mPneumonia, an Innovation for Diagnosing and Treating Childhood Pneumonia in Low-Resource Settings: A Feasibility, Usability and Acceptability Study in Ghana. Simeoni U, editor. PLoS One. 2016 Oct 27;11(10):e0165201.

10. Nielson J. Usability engineering. San Diego, CA: Academic Press Inc.; 1993.

11. Braun V, Clarke V. Using thematic analysis in psychology. Qual Res Psychol. 2006;3(2):77–101.

12. Zhu Z, Liu T, Li G, Li T, Inoue Y. Wearable Sensor Systems for Infants. Sensors. 2015 Feb 5;15(2):3721–49.

13. Memon SF, Memon M, Bhatti S. Wearable technology for infant health monitoring: A survey. IET Circuits, Devices Syst. 2020;14(2):115–29.

For peer review only - http://bmjopen.bmj.com/site/about/guidelines.xhtml

14. Greenhalgh T, Abimbola S. The NASSS Framework – A Synthesis of Multiple Theories of Technology Implementation. Stud Health Technol Inform. 2019;263:193–204.

15. Salimu S, Kinshella MLW, Vidler M, Banda M, Newberry L, Dube Q, et al. Health workers' views on factors affecting caregiver engagement with bubble CPAP. BMC Pediatr. 2020 Apr 23;20(1):180.

16. Kinshella MLW, Walker CR, Hiwa T, Vidler M, Nyondo-Mipando AL, Dube Q, et al. Barriers and facilitators to implementing bubble CPAP to improve neonatal health in sub-Saharan Africa: A systematic review. Public Health Rev. 2020 Apr 28;41(1):6.

17. Leonard E, de Kock I, Bam W. Barriers and facilitators to implementing evidence-based health innovations in low- and middle-income countries: A systematic literature review. Eval Program Plann. 2020 Oct 1;82:101832.

18. Gathara D, Serem G, Murphy GAV, Obengo A, Tallam E, Jackson D, et al. Missed nursing care in newborn units: a cross-sectional direct observational study. BMJ Qual Saf. 2020 Jan 1;29(1):19–30.

For peer review only

For peer review only - http://bmjopen.bmj.com/site/about/guidelines.xhtml

FIGURE LEGEND

Figure 1: Overview of the three multiparameter continuous physiological monitoring technologies

For peer review only - http://bmjopen.bmj.com/site/about/guidelines.xhtml

Figure 1. Overview of the three multiparameter continuous physiological monitoring technologies

INVESTIGATIONAL TECHNOLOGIES	
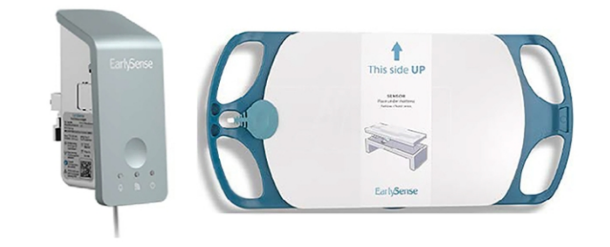	**EarlySense**<br>- Non-contact mattress pad attached to main power outlet<br>- Continuous monitoring of respiratory rate and heart rate wirelessly transmitted to an external screen<br>- Commercially available and used in health facilities in Europe and North America for adult and pediatric patients
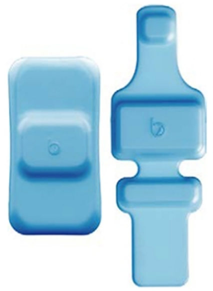	**Sibel**<br>- Set of two wireless, reusable, self-contained, direct contact skin sensors with rechargeable batteries<br>- Together, chest and limb sensors monitor respiratory rate heart rate, bio-impedance, pulse oximtery, and movement<br>- Disposable hydrogel adhesive and Velcro bands keep sensors on the neonate<br>- Data saved locally on sensor and with live showing of vital signs via iPad
REFERENCE TECHNOLOGY	
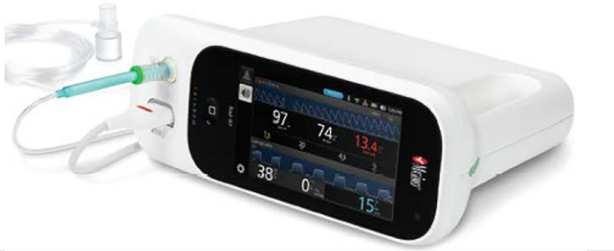	**Masimo Rad-97**<br>- Disposable skin sensor for heart rate and pulse oximetry<br>- Nasal capnography records carbon dioxide levels and measures respiratory rate<br>- Commercially available and extensively used worldwide in patients of all ages

Overview of three multiparameter continuous physiological monitoring technologies

100x104mm (300 x 300 DPI)

S2 File ETNA Qualitative Study Interview Guides

2.1 In-Depth Interview Guide – Caregiver

**Administrative information**	
**Caregiver ID number:**	**Sex:** ☐ Female ☐ Male
**Date caregiver informed consent form (ICF) signed:** |D|D| - |M|M|M| - |Y|Y|Y|Y|	
**Caregiver ICF signed prior to any study questions?** ☐ Yes ☐ No	
**Name of research staff who explained the ICF:**	
**Does the caregiver agree to be audio recorded?** ☐ Yes ☐ No<br>If Yes, was the interview audio recorded? ☐ Yes ☐ No<br>If No, why was the interview not recorded? ________________	
**Name of interviewer:**	
**Date of interview:** |D|D| - |M|M|M| - |Y|Y|Y|Y|	
**Location of interview:**<br>☐ **Aga Khan University – Nairobi Hospital**<br>☐ **Pumwani Maternity Hospital**	
**Interview start time:** |H|H| : |M|M| *military time*	

<u>Instructions for qualitative research staff</u>:

Use this document as a guide to conduct the interviews with the **caregivers**.
Conduct the interview in the language with which the caregiver feels most comfortable.
The interview should take place in a quiet place that allows privacy.
Please introduce each question separately. The interview must flow as a conversation. If you notice that the caregiver is hesitant in answering, does not give an in-depth response, or the response is not satisfactory, please probe or ask follow-up questions, but do NOT prompt any specific answer. Several probes are suggested, and you may also ask follow-up questions that are not listed in this guide but are necessary for the complete expression of the caregiver's views.
Please **record the interview** using the audio recorder (if caregiver consent is provided) and state the ETNA Caregiver ID number.
All comments from the caregiver should be recorded/written verbatim.
Please cross-check the narratives written with the recorded version as a reference and correct as necessary.
All responses must be kept confidential. Do not discuss or share responses with anyone outside of the ETNA study team.

Script to initiate the interview

"Hello, my name is ___________ and I am a researcher with the ETNA project and we are evaluating monitoring devices for newborns. We want to hear about your experiences and learn from your thoughts and feelings. We will keep what you tell us today confidential, which means that nothing you say will be directly linked to you so please feel free to share. If you feel uncomfortable with any questions, let me know and we will skip it. Before we start, do you have any questions for me? Is it ok to begin? Thank you, I will start the audio-recording now."

A. Demographic information

For peer review only

1. First, we will start with some questions about yourself, what is your age?
2. Did you attend any schooling? If so, what class (level) did you complete?
3. Would you be able to tell us a little about yourself and what you/your husband do for a living?
4. Where do you and your family live? How far away is it from this hospital?
5. How many people live together in your house and what is their relationship to you?
6. How many children do you have? What is your role in caring for your newborn?

B. Birth history, pregnancy and healthcare facility experience
If this some of this information could be abstracted from patient hospital records, could consider using these records as a resource BEFORE the interview. Otherwise, please ask these questions.

1. How many pregnancies have you (or your wife, daughter-in-law, daughter) had? How many live births?
2. We would like to learn more about your experience with your most recent pregnancy. Could you tell us if you had any issues or complications during the pregnancy, labor or delivery?

Probes: What were your symptoms during pregnancy, length of labor, mode of delivery, how long admitted to the hospital?

3. Was your baby born early? If yes, do you know how early? (Another way to phrase this is "When were you expecting the baby and was the baby born earlier?")
4. Did your newborn have any health issues when he/she was born? If yes, what were they?

Probes: Examples include low birth weight (kangaroo mother care), infection at time of birth, birth defect, respiratory distress (trouble breathing), neonatal jaundice (put under the blue light), inability to breastfeed, etc.

5. What healthcare services did you and your newborn receive here at the hospital?
6. Are you happy with the quality of care you and your newborn received at this hospital? Could you explain with an example? What do you think could make the quality of care at this hospital better?

Probes: How did the staff treat you and your family? Did they seem trained/knowledgeable? Did they have enough equipment/supplies to care for you and your newborn?

7. How did you get to this hospital and how long did it take you to get here from your home? Why did you and your family decide to come to this hospital for delivery (or newborn care depending on their narrative)?
8. What other health facilities do you usually go to when you or your family needs medical care? When do you go to those other health facilities instead of this hospital?

C. Monitoring devices

1. What are your experiences with how healthcare providers monitor newborns receiving care at this hospital? How often do they come by to check your newborn and what do they usually check?
2. Do healthcare providers use any devices or technologies when they are doing a checkup on your newborn?
3. Are there any devices or machines that you are aware of that are used to monitor the newborn between checks by the healthcare provider?
4. Do you have any concerns about these devices? If so, could you explain with an example?

D. EarlySense InSight investigational device

Research staff shows EarlySense InSight device to the caregiver and explains how it works.
"The EarlySense InSight device is a contact-free newborn monitoring system. The system includes a sensor pad that is placed under the newborn's mattress to measure heart rate, breathing rate, motion, and sleep status. There is no physical contact between the newborn and the sensor pad. Information from the sensor pad is continuously transmitted to a monitor or tablet that can be read by hospital staff."

For peer review only

Allow them to touch the InSight device, the mattress pad and the cable that goes between them.

1. Have you ever seen this device before? Was this device used in the care of your newborn?
   If yes,
   a. What was the first thought that came to your mind when the healthcare provider told you about this device? How did you feel about this device being used for your newborn? How do you feel about it now? How did your husband (or family) react when they learned that your newborn was on this device?
   b. What did you like (if anything) about this device and how it was used? What did you dislike (if anything) about this device and how it was used? Please explain.
   c. Did the healthcare provider using the device run into any difficulties? What did they do?
   If no,
   a. Imagine if a doctor recommended using this device for your newborn, how would you feel? What do you think your husband (or family) would think if your newborn was put on this device?
2. Are there any problems you can think of with this device? Any concerns or parts/features you think might be harmful to newborns? In what situations? Please explain.
3. Do you think this device should be used in the care of newborns in this hospital? Why or why not?
4. Do you see any problems with using this device at this hospital? If so, could you explain with an example?
5. If there was a healthcare facility that used this device regularly to help care for newborns, would that make you want to go to that facility more or less? Why?

E. Sibel ANNE investigational device

Research staff shows Sibel ANNE device to caregiver and explains how it works.
"The Sibel ANNE device uses non-invasive sensors to continuously measure and record a newborn's heart rate, breathing rate, level of oxygen in the blood, and skin temperature. One sensor is attached to the newborn's chest and contains a battery. The second sensor is battery-free, ultra-thin, and is applied to the newborn's hand or foot. Information from the sensors is wirelessly transmitted to a monitor or tablet that can be read by hospital staff."
Allow them to touch the ANNE chest and limb sensors, the hydrogel, and iPad display fully.

1. Have you ever seen this device before? Was this device used in the care of your newborn?
   If yes,
   a. What was the first thought that came to your mind when the healthcare provider told you about this device? How did you feel about this device being used for your newborn? How do you feel about it now? How did your husband (or family) react when they learned that your newborn was on this device?
   b. What did you like (if anything) about this device and how it was used? What did you dislike (if anything) about this device and how it was used? Please explain.
   c. Did the healthcare provider using the device run into any difficulties? What did they do?
   If no,
   a. Imagine if a doctor recommended using this device for your newborn, how would you feel? What do you think your husband (or family) would think if your newborn was put on this device?
2. Are there any problems you can think of with this device? Any concerns or parts/features you think might be harmful to newborns? In what situations? Please explain.
3. Do you think this device should be used in the care of newborns in this hospital? Why or why not?
4. Do you see any problems with using this device at this hospital? If so, could you explain with an example?
5. If there was a healthcare facility that used this device regularly to help care for newborns, would that make you want to go to that facility more or less? Why

F. Masimo RAD-97 reference device
**Research staff shows Masimo Rad-97 device to caregiver and explains how it works.**<br>*"The Masimo Rad-97 is a non-invasive device that measures a newborn's heart rate, breathing rate, and level of oxygen in the blood. Information is collected through a skin sensor that is applied to the newborn's hand or foot and a tube that is inserted into the newborn's nostrils. The information is then continuously transmitted to a monitor that can be read by hospital staff."*<br>**Allow them to touch the Rad-97, skin sensor and capnography tube fully.**<br>1. Have you ever seen this device before? Was this device used in the care of your newborn?<br>**If yes,**<br>a. What was the first thought that came to your mind when the healthcare provider told you about this device? How did you feel about this device being used for your newborn? How do you feel about it now? How did your husband (or family) react when they learned that your newborn was on this device?<br>b. What did you like (if anything) about this device and how it was used? What did you dislike (if anything) about this device and how it was used? Please explain.<br>c. Did the healthcare provider using the device run into any difficulties? What did they do?<br>**If no,**<br>a. Imagine if a doctor recommended using this device for your newborn, how would you feel? What do you think your husband (or family) would think if your newborn was put on this device?<br>2. Are there any problems you can think of with this device? Any concerns or parts/features you think might be harmful to newborns? In what situations? Please explain.<br>3. Do you think this device should be used in the care of newborns in this hospital? Why or why not?<br>4. Do you see any problems with using this device at this hospital? If so, could you explain with an example?<br>5. If there was a healthcare facility that used this device regularly to help care for newborns, would that make you want to go to that facility more or less? Why

H. Closing
1. Taking into consideration the monitoring devices we have talked about today, could you rank the device (if any) you think is the best, second best and third choice in your opinion? Please explain why.<br>2. Do you have any other comments about any of the three devices that we did not talk about?<br>3. Do you have any other comments about newborn monitoring devices or any other comments or concerns overall that we did not get to talk about?
"Thank you for your time and the helpful information you have provided. Your feedback, along with feedback from other people we talk to will be used to recommend solutions for better care."
**Interview end time:** |H|H| : |M|M| *military time*

2.2 In-Depth Interview Guide – Healthcare Administrator (HCA)

Administrative information	
**HCA ID number:**	**Sex:** ☐ Female ☐ Male
**Date HCA informed consent form (ICF) signed:** |D|D| - |M|M|M| - |Y|Y|Y|Y|	
**HCA ICF signed prior to any study questions?** ☐ Yes ☐ No	

For peer review only

**Name of research staff who explained the ICF:**
**Does the HCA agree to be audio recorded?** ☐ Yes ☐ No<br>If Yes, was the interview audio recorded? ☐ Yes ☐ No<br>If No, why was the interview not recorded? ____________
**Name of interviewer:**
**Date of interview:** |D|D| - |M|M|M| - |Y|Y|Y|Y|
**Location of interview:**<br>☐ **Aga Khan University – Nairobi Hospital**<br>☐ **Pumwani Maternity Hospital**<br>☐ **Other:** ____________
**Interview start time:** |H|H| : |M|M| *military time*

<u>Instructions for qualitative research staff</u>:

- Use this document as a guide to conduct the interviews with the **healthcare administrators (HCA)**.
- Conduct the interview in the language with which the HCA feels most comfortable.
- The interview should take place in a quiet place that allows privacy.
- Please introduce each question separately. The interview should flow as a conversation. If you notice that the HCA is hesitant in answering, does not give an in-depth response, or the response is not satisfactory, please probe or ask follow-up questions, but do NOT prompt any specific answer. Several probes are suggested, and you may also ask follow-up questions that are not listed in this guide but are necessary for the complete expression of the HCA's views.
- Please **record the interview** using the audio recorder (if HCA consent is provided) and state the ETNA HCA ID number.
- All comments from the HCA should be recorded/written verbatim.
- Please cross-check the narratives written with the recorded version as a reference and correct as necessary.
- All responses must be kept confidential. Do not discuss or share responses with anyone outside of the ETNA study team.

Script to initiate the interview

"Hello, my name is ___________ and I am a researcher with the ETNA project and we are evaluating monitoring devices for newborns. We want to hear about your experiences and learn from your thoughts and feelings. We will keep what you tell us today confidential, which means that nothing you say will be directly linked to you so please feel free to share. If you feel uncomfortable with any questions, let me know and we will skip it. Before we start, do you have any questions for me? Is it ok to begin? Thank you, I will start the audio-recording now."

G. Demographic information

1. First, we will start with some questions about yourself, what is your age?
2. How many years of education and training have you received and what is your highest level of education completed? What is your medical background (e.g., doctor, nurse, technician, etc.)? Were you ever involved in patient care? In the care of newborns?
3. How long did you work in the medical field before working as a healthcare administrator? How long have you been working as a healthcare administrator?

H. Healthcare administrator role

1. What is your job title and current role here at this healthcare facility? How long (years, months if less than one year) have you been in the current position at this facility?
2. What are your responsibilities as a healthcare administrator at this facility?
Probes: What is your involvement (if any) in policy development for newborn care such as creating new protocols and/or adapting national guidelines? Please share what a typical day as a hospital administrator would be like for you.

I. Facility

1. What is the process of purchasing medical equipment at this healthcare facility?
Probes: Who makes the decision to identify what medical equipment will be used in the hospital? Who makes the decisions on what to purchase? Are these decisions made on an individual hospital basis or decided at a local or national level by Ministry of Health?
2. What are the current constraints (if any) to providing care to newborns at this facility?
Probes: What makes care more difficult? What would make it easier?
3. Does this facility have reliable access to electricity? When was the last electricity outage and how long do they typically last? What happens during power outages at your facility?
Probes: How do power outages affect patient care? Is there a back-up power supply? If so, what is the process of using the backup power supply and are there any issues around its use (e.g., does it cover all of the equipment needed, any issues in getting permission for its use, fuel prices? Any voltage issues?)
4. Are you aware of any technologies that are being used in the delivery and newborn care wards at this facility, and if so, can you describe them? What are some concerns you have, or gaps in the technologies available, for maternal and newborn care at this facility?
Probes: Which healthcare providers use the technologies? What technologies/brands are used? Do healthcare providers use any handheld or portable devices for maternal or newborn care (e.g., tablets or Smartphones)? If yes, please describe the technologies and their use.

J. Monitoring devices

We would especially like to learn about your perspectives on continuous monitoring devices.

1. Before this study, had you used continuous monitoring devices or seen them in use? Are you aware of any continuous monitoring devices being used at this healthcare facility outside of the ETNA study?
Probes: If yes, where in the facility? For what purpose? How frequently are they used?
2. What do you think are some of the benefits of using continuous monitoring devices at your facility? What impacts do you think they have (if in current use) or would have (if not in current use) on routine care at this facility?
3. Do you have any concerns about using continuous monitoring devices? What are the challenges to using such devices at this facility? Are there any situations you think the use of monitoring devices would not be useful? If so, can you explain with an example?
Probes: Tell me about how newborns are monitored in your facility? How is this different (if at all) for sick newborns?
4. What do you think would be needed to scale up the use of continuous monitoring devices at this facility? What enablers do you think could support this process?
5. Imagine if monitoring devices were scaled up at this facility, how do you think the nurses and doctors that work here would react? How do you think caregivers (mothers, parents, guardians, etc.) would react? What about outside stakeholders and decision-makers at local, county, and national levels?

K. EarlySense InSight investigational device

Research staff shows EarlySense InSight device to HCA and explains how it works.

For peer review only

"The EarlySense InSight device is a contact-free newborn monitoring system. The system includes a sensor pad that is placed under the newborn's mattress to measure heart rate, breathing rate, motion, and sleep status. There is no physical contact between the newborn and the sensor pad. Information from the sensor pad is continuously transmitted to a monitor or tablet that can be read by hospital staff. The system has been previously tested for safety in neonates."
Allow them to touch the InSight device, the mattress pad and the cable that goes between them.

6. Could you share what you like (if anything) about this device? What do you think would be useful in the care of newborns at your healthcare facility?
7. Could you share what you dislike (if anything) about this device? What about the device do you think could create difficulties in caring for newborns at your facility?
8. Do you think this device is suitable for use in your facility? What do you think would need to happen in order to successfully use this device in your facility?
   Probes: For example, staffing availability and skill to use the device, training, complexity of the device, availability of equipment and infrastructure needed for its use, durability and maintenance of device and components, access to spare parts, protocols and guidelines for use, counselling caregivers and informational materials? What could be the benefits/drawbacks?
9. How do you think healthcare providers, like doctors and nurses, would feel about this device?
10. How do you think caregivers, such as mothers, fathers, mothers-in-law and other family members, would feel about this device?
11. How do you think other healthcare administrators and decision-makers at local, county and national levels would react to a recommendation to implement this device at this facility
12. In your opinion, how much would your facility pay for a device like this? (Circle Response)

<$5000 KSh	$5000 – $10000 KSh	$10000 – $15000 KSh
$15000 - $20000 KSh	$20000 - $25000 KSh	>$25000 KSh

Please explain

L. Sibel ANNE investigational device

Research staff shows Sibel ANNE device to HCA and explains how it works.
"The Sibel ANNE device uses non-invasive sensors to continuously measure and record a newborn's heart rate, breathing rate, level of oxygen in the blood, and skin temperature. One sensor is attached to the newborn's chest and contains a battery. The second sensor is battery-free, ultra-thin, and is applied to the newborn's hand or foot. Information from the sensors is wirelessly transmitted to a monitor or tablet that can be read by hospital staff. The system has previously been tested for safety in neonates."
Allow them to touch the ANNE chest and limb sensors, the hydrogel, and iPad display fully.

1. Could you share what you like (if anything) about this device? What do you think would be useful in the care of newborns at your healthcare facility?
2. Could you share what you dislike (if anything) about this device? What about the device do you think could create difficulties in caring for newborns at your facility?
3. Do you think this device is suitable for use in your facility? What do you think would need to happen in order to successfully use this device in your facility?
   Probes: For example, staffing availability and skill to use the device, training, complexity of the device, availability of equipment and infrastructure needed for its use, durability and maintenance of device and components, access to spare parts, protocols and guidelines for use, counselling caregivers and informational materials? What could be the benefits/drawbacks?
4. How do you think healthcare providers, like doctors and nurses, would feel about this device?
5. How do you think caregivers, such as mothers, fathers, mothers-in-law and other family members, would feel about this device?

For peer review only

6. How do you think other healthcare administrators and decision-makers at local, county and national levels would react to a recommendation to implement this device at this facility?
7. In your opinion, how much would your facility pay for a device like this? (Circle Response)

<$5000 KSh	$5000 – $10000 KSh	$10000 – $15000 KSh	
$15000 - $20000 KSh	$20000 - $25000 KSh	>$25000 KSh	

Please explain

M. Masimo RAD-97 reference device

Research staff shows Masimo Rad-97 device to HCA and explains how it works.

"The Masimo Rad-97 is a non-invasive device that measures a newborn's heart rate, breathing rate, and level of oxygen in the blood. Information is collected through a skin sensor that is applied to the newborn's hand or foot and a tube that is inserted into the newborn's nostrils. The information is then continuously transmitted to a monitor that can be read by hospital staff. The system has previously been tested for safety in neonates."

Allow them to touch the Rad-97, skin sensor and capnography tube fully.

1. Could you share what you like (if anything) about this device? What do you think would be useful in the care of newborns at your healthcare facility?
2. Could you share what you dislike (if anything) about this device? What about the device do you think could create difficulties in caring for newborns at your facility?
3. Do you think this device is suitable for use in your facility? What do you think would need to happen in order to successfully use this device in your facility?
   Probes: For example, staffing availability and skill to use the device, training, complexity of the device, availability of equipment and infrastructure needed for its use, durability and maintenance of device and components, access to spare parts, protocols and guidelines for use, counselling caregivers and informational materials? What could be benefits/drawbacks?
4. How do you think healthcare providers, like doctors and nurses, would feel about this device?
5. How do you think caregivers, such as mothers, fathers, mothers-in-law and other family members, would feel about this device?
6. How do you think other healthcare administrators and decision-makers at local, county and national levels would react to a recommendation to implement this device at this facility?
7. In your opinion, how much would your facility pay for a device like this? (Circle Response)

<$5000 KSh	$5000 – $10000 KSh	$10000 – $15000 KSh
$15000 - $20000 KSh	$20000 - $25000 KSh	>$25000 KSh

Please explain.

H. Closing

1. Taking into consideration the three monitoring devices we have talked about today, can you rank the device (if any) you think is the best, second best and third choice in your opinion? Please explain why.
2. In terms of feasibility, which device (if any) do you think would be the most appropriate device for your healthcare facility and why?
3. In terms of acceptability, which device (if any) do you think healthcare providers would like the best and why? Which device (if any) do you think caregivers would prefer and why?
4. Do you have any other comments about any of the three devices that we did not talk about?
5. Do you have any other comments about newborn monitoring devices or any other comments or concerns overall that we did not get to talk about?

"Thank you for your time and the helpful information you have provided. Your feedback, along with feedback from other people we talk to will be used to recommend solutions for better care."

**Interview end time:** |H|H| : |M|M| *military time*

2.3 In-Depth Interview Guide – Healthcare Provider (HCP) Direct Use

**Administrative information**	
**HCP ID number:**	**Sex:** ☐ Female ☐ Male
**Date HCP informed consent form (ICF) signed:** |D|D| - |M|M|M| - |Y|Y|Y|Y|	
**HCP ICF signed prior to any study questions?** ☐ Yes ☐ No	
**Name of research staff who explained the ICF:**	
**Does the HCP agree to be audio recorded?** ☐ Yes ☐ No<br>If Yes, was the interview audio recorded? ☐ Yes ☐ No<br>If No, why was the interview not recorded? ___________________________	
**Name of interviewer:**	
**Date of interview:** |D|D| - |M|M|M| - |Y|Y|Y|Y|	
**Location of interview:**<br>☐ **Aga Khan University – Nairobi Hospital**<br>☐ **Pumwani Maternity Hospital**	
**Interview start time:** |H|H| : |M|M| *military time*	

Instructions for qualitative research staff:

- Use this document as a guide to conduct the interviews with **healthcare providers (HCP) directly using the devices**.
- Conduct the interview in the language with which the HCP feels most comfortable.
- The interview should take place in a quiet place that allows privacy.
- Please introduce each question separately. The interview must flow as a conversation. If you notice that the HCP is hesitant in answering, does not give an in-depth response, or the response is not satisfactory, please probe or ask follow-up questions, but do NOT prompt any specific answer. Several probes are suggested, and you may also ask follow-up questions that are not listed in this guide but are necessary for the complete expression of the HCP's views.
- Please **record the interview** using the audio recorder (if HCP consent is provided) and state the ETNA HCP ID number.
- All comments from the HCP should be recorded/written verbatim.
- Please cross-check the narratives written with the recorded version as a reference, and correct as necessary.
- All responses must be kept confidential. Do not discuss or share responses with anyone outside of the ETNA study team.

**Script to initiate the interview**
"Hello, my name is ___________ and I am a researcher with the ETNA project and we are evaluating monitoring devices for newborns. We want to hear about your experiences and learn from your thoughts and feelings. We will keep what you tell us today confidential, which means that nothing you say will be directly linked to you so please feel free to share. If you feel uncomfortable with any questions, let me know and we will skip it. Before we start, do you have any questions for me? Is it ok to begin? Thank you, I will start the audio-recording now."

For peer review only - http://bmjopen.bmj.com/site/about/guidelines.xhtml

N. Demographic information
7. First, we will start with some questions about yourself, what is your age?<br>8. How many years of education and training have you completed and what is your highest level of education completed? What is your medical background/designation? (e.g., doctor, nurse, technician, etc.)<br>9. How long have you worked as ‘doctor/nurse/technician/etc.?

O. Healthcare provider role
4. How long have you been employed at this healthcare facility?<br>5. What is your job title and current role here at this facility? How long have you been in this role at this facility?<br>6. What are your responsibilities in this role?<br>*Probes: Please share what a typical day as a healthcare provider would be like for you.*<br>*7.* Are you involved in patient care? If yes, please explain your patient care responsibilities.

P. Facility
5. What are the current constraints to providing care to newborns at this healthcare facility?<br>*Probes: What makes care more difficult? What would make it easier?*<br>6. How are newborns monitored at this facility? How is this different (if at all) for sick newborns?<br>7. Does this facility have reliable access to electricity? When was the last electricity outage and how long do they typically last? What happens during power outages at your facility?<br>*Probes: How do power outages affect patient care? Is there a back-up power supply? If so, what is the process of using the backup power supply and are there any issues around its use (e.g., does it cover all of the equipment needed, any issues in getting permission for its use, fuel prices? Any voltage issues?*<br>8. Do you have regular access to computers at this facility? If yes, do they work well?<br>*Probes: Do computers breakdown often? If yes, please describe how the computer breakdowns affect your work as a healthcare provider?*<br>9. Could you describe the technologies that are being used in the delivery and newborn care wards at this facility? What are some concerns you have, or gaps in the technologies available, for maternal and newborn care at this facility?<br>*Probes: Which healthcare providers use the technologies? What technologies/brands are used? Do healthcare providers use any handheld or portable devices for maternal or newborn care (e.g., tablets or Smartphones)? If yes, please describe the technologies and their use.*

Q. Monitoring devices
1. What is your role with the Evaluation of Technologies for Neonates in Africa (ETNA) research study? What are your ETNA-related responsibilities?<br>**We would especially like to learn about your perspectives on continuous monitoring devices.**<br>2. Before this study, had you used continuous monitoring devices or seen them used? Tell me about your experience with continuous monitoring devices.<br>*Probes: List devices used, then discuss each device sequentially (where used, for what purpose?). How frequently have you used these types of devices? Did you find them to be useful? If yes, how so? If no, why not? What sort of training did you receive for the use of these devices?*<br>3. Apart from the devices used in the ETNA study, are continuous monitoring devices used at this healthcare facility?<br>*Probes: If yes, where in the facility? For what purpose? How frequently do you use these devices?*<br>4. What do you think are some of the benefits of using continuous monitoring devices? What impacts do you think they could have on routine care at this facility?

For peer review only

5. Do you have any concerns about using continuous monitoring devices? What are the challenges to using such devices at this facility? Are there any situations you think the use of monitoring devices would not be useful? If so, could you explain with an example?
6. What do you think would be needed to scale up the use of continuous monitoring devices at this facility? What enablers do you think could support this process?
7. Imagine if continuous monitoring devices were scaled up at this facility, how do you think the nurses and doctors that work here would react? How do you think caregivers (mothers, parents, guardians, etc.) would react?

E. EarlySense InSight investigational device

The next set of question will focus on your experiences with the EarlySense InSight device.

Usability

13. Do you think that healthcare providers in this facility could develop the skills necessary to use this continuous monitoring device? Why or why not?
14. What sort of training did you receive on this device before you began using it?
    Probes: Please describe length and method of training. Who provided training? Was training adequate? What additional training do you wish you had received? What sort of training do you think would be required for healthcare providers in this facility to use this device?
15. Which aspects of using this device were easy to learn? Which aspects were difficult?
    Probes: Did using the device become easier or more difficult over time?
16. If you now feel comfortable using the device, how long did it take you to become comfortable? If not comfortable, why not?
17. What kind of support did you receive during this period? Please explain.
    Probes: From device manufacturers, supervisors, coworkers, etc.?
18. What did you like (if anything) about this device overall? What did you dislike (if anything)? Are there any changes you would make to this device? If so, what are they?
    Probes: For example, overall device setup/interface, ease of use, etc.? What about the different features: InSIght device, mattress pad, cable, mobile application, monitor screen/display, etc.?
19. Did this device make providing care to newborns at this facility easier or more difficult? How so?
    Probes: For example, enable more care, interrupt care, etc.
20. Do you think the device would make care easier or more difficult if you could use the information collected and displayed by this device? How so?
21. Were there any questions you had about this device while you were using it? Please explain.
22. Did caregivers or other hospital staff ask you any questions about this device while you were using it? If so, what did they ask?
23. Are there situations where you think this device should not be used? If so, what are they?

Acceptability

24. How do you think other healthcare providers, like doctors and nurses, would feel about this device?
25. Based on your encounters with caregivers, such as mothers, fathers, mothers-in-law and other family members, how do you think they would feel about this device?
26. How do you think healthcare administrators and decision-makers at local, district and national levels would react to a recommendation to implement this device at this facility?
    Probes: Discuss at each level (local, district, national) sequentially. What stakeholders would influence the uptake of this technology?
27. Do you think healthcare providers would consider information collected and displayed by this device trustworthy? Why or why not?
28. Would you like to see your facility incorporate this device into newborn care? Why or why not?

Feasibility

29. Do you think this device is suitable for your facility? What would need to happen in order to integrate this device successfully at this facility? Please explain.

For peer review only

Probes: For example, staffing availability and skill to use the device, training, complexity of the device, availability of equipment and infrastructure needed for its use, durability and maintenance of device and components, access to spare parts, protocols and guidelines for use, counselling caregivers and informational materials? What could be benefits/drawbacks?
Probes: For example, ease of use during a patient visit, integration into current flow of hospital operations, acceptance by administrators, etc.?

30. Do you have any other comments about this device that we did not talk about?

F. Sibel ANNE investigational device

The next set of question will focus on your experiences with the Sibel ANNE device.

Usability

1. Do you think that healthcare providers in this facility could develop the skills necessary to use this continuous monitoring device? Why or why not?
2. What sort of training did you receive on this device before you began using it?
   Probes: Please describe length and method of training. Who provided training? Was training adequate? What additional training do you wish you had received? What sort of training do you think would be required for healthcare providers in this facility to use this device?
3. Which aspects of using this device were easy to learn? Which aspects were difficult?
   Probes: Did using the device become easier or more difficult over time?
4. If you now feel comfortable using the device, how long did it take you to become comfortable? If not comfortable, why not?
5. What kind of support did you receive during this period? Please explain.
   Probes: From device manufacturers, supervisors, coworkers, etc.?
6. What did you like (if anything) about this device overall? What did you dislike (if anything)? Are there any changes you would make to this device? If so, what are they?
   Probes: For example, overall device setup/interface, ease of use, etc.? What about the different features: chest and limb sensors, hydrogel, mobile application, iPad screen/display, etc.?
7. Did this device make providing care to newborns at this facility easier or more difficult? How so?
   Probes: For example, enable more care, interrupt care, etc.
8. Do you think the device would make care easier or more difficult if you could use the information collected and displayed by this device? How so?
9. Were there any questions you had about this device while you were using it? Please explain.
10. Did caregivers or other hospital staff ask you any questions about this device while you were using it? If so, what did they ask?
11. Are there situations where you think this device should not be used? If so, what are they?

Acceptability

12. How do you think other healthcare providers, like doctors and nurses, would feel about this device?
13. Based on your encounters with caregivers, such as mothers, fathers, mothers-in-law and other family members, how do you think they would feel about this device?
14. How do you think healthcare administrators and decision-makers at local, district and national levels would react to a recommendation to implement this device at this facility?
    Probes: Discuss at each level (local, district, national) sequentially. What stakeholders would influence the uptake of this technology?
15. Do you think healthcare providers would consider information collected and displayed by this device trustworthy? Why or why not?
16. Would you like to see your facility incorporate this device into newborn care? Why or why not?

Feasibility

17. Do you think this device is suitable within your facility? What would need to happen in order to integrate this device successfully at this facility? Please explain.

For peer review only

Probes: For example, staffing availability and skill to use the device, training, complexity of the device, availability of equipment and infrastructure needed for its use, durability and maintenance of device and components, access to spare parts, protocols and guidelines for use, counselling caregivers and informational materials? What could be benefits/drawbacks?   *Probes: For example, ease of use during a patient visit, integration into current flow of hospital operations, acceptance by administrators, etc.?*   18. Do you have any other comments about this device that we did not talk about?

G. Masimo RAD-97 reference device

The next set of question will focus on your experiences with the Masimo Rad-97 device.

Usability

1. Do you think that healthcare providers in this facility could develop the skills necessary to use this continuous monitoring device? Why or why not?
2. What sort of training did you receive on this device before you began using it?
   Probes: Please describe length and method of training. Who provided training? Was training adequate? What additional training do you wish you had received? What sort of training do you think would be required for healthcare providers in this facility to use this device?
3. Which aspects of using this device were easy to learn? Which aspects were difficult?
   Probes: Did using the device become easier or more difficult over time?
4. If you now feel comfortable using the device, how long did it take you to become comfortable? If not comfortable, why not?
5. What kind of support did you receive during this period? Please explain.
   Probes: From device manufacturers, supervisors, coworkers, etc.?
6. What did you like (if anything) about this device overall? What did you dislike (if anything)? Are there any changes you would make to this device? If so, what are they?
   Probes: For example, overall device setup/interface, ease of use, etc.? What about the different features: Rad-97 device, skin sensor, capnography tube, mobile application, monitor screen/display, etc.?
7. Did this device make providing care to newborns at this facility easier or more difficult? How so?
   Probes: For example, enable more care, interrupt care, etc.
8. Do you think the device would make care easier or more difficult if you could use the information collected and displayed by this device? How so?
9. Were there any questions you had about this device while you were using it? Please explain.
10. Did caregivers or other hospital staff ask you any questions about this device while you were using it? If so, what did they ask?
11. Are there situations where you think this device should not be used? If so, what are they?

Acceptability

12. How do you think other healthcare providers, like doctors and nurses, would feel about this device?
13. Based on your encounters with caregivers, such as mothers, fathers, mothers-in-law and other family members, how do you think they would feel about this device?
14. How do you think healthcare administrators and decision-makers at local, district and national levels would react to a recommendation to implement this device at this facility?
    Probes: Discuss at each level (local, district, national) sequentially. What stakeholders would influence the uptake of this technology?
15. Do you think healthcare providers would consider information collected and displayed by this device trustworthy? Why or why not?
16. Would you like to see your facility incorporate this device into newborn care? Why or why not?

Feasibility

For peer review only

17. Do you think this device is suitable within your facility? What would need to happen in order to integrate this device successfully at this facility? Please explain.<br>*Probes: For example, staffing availability and skill to use the device, training, complexity of the device, availability of equipment and infrastructure needed for its use, durability and maintenance of device and components, access to spare parts, protocols and guidelines for use, counselling caregivers and informational materials? What could be benefits/drawbacks?*<br>*Probes: For example, ease of use during a patient visit, integration into current flow of hospital operations, acceptance by administrators, etc.?*<br>18. Do you have any other comments about this device that we did not talk about?

**H. Closing**
6. Taking into consideration the three monitoring devices we have talked about today, could you rank the device (if any) you think is the best, second best and third choice in your opinion? Please explain why.<br>7. In terms of feasibility, which device (if any) do you think would be the most appropriate device for your healthcare facility and why?<br>8. In terms of acceptability, which device (if any) do you think healthcare providers would like the best and why? Which device (if any) do you think caregivers would prefer and why?<br>9. Do you have any other comments about any of the three devices that we did not talk about?<br>10. Do you have any other comments about newborn monitoring devices or any other comments or concerns overall that we did not get to talk about?
"Thank you for your time and the helpful information you have provided. Your feedback, along with feedback from other people we talk to will be used to recommend solutions for better care."
**Interview end time:** |H|H| : |M|M| *military time*

2.4 In-Depth Interview Guide – Healthcare Provider (HCP) In-Direct Use

**Administrative information**	
**HCP ID number:**	**Sex:** ☐ Female ☐ Male
**Date HCP informed consent form (ICF) signed:** |D|D| - |M|M|M| - |Y|Y|Y|Y|	
**HCP ICF signed prior to any study questions?** ☐ Yes ☐ No	
**Name of research staff who explained the ICF:**	
**Does the HCP agree to be audio recorded?** ☐ Yes ☐ No<br>If Yes, was the interview audio recorded? ☐ Yes ☐ No<br>If No, why was the interview not recorded? ______________________	
**Name of interviewer:**	
**Date of interview:** |D|D| - |M|M|M| - |Y|Y|Y|Y|	
**Location of interview:**<br>☐ **Aga Khan University – Nairobi Hospital**	

For peer review only - http://bmjopen.bmj.com/site/about/guidelines.xhtml

☐ **Pumwani Maternity Hospital**

Interview start time: |H|H| : |M|M| *military time*

<u>Instructions for qualitative research staff</u>:

Use this document as a guide to conduct the interviews with the **healthcare providers (HCP) not directly using the devices**.

Conduct the interview in the language with which the HCP feels most comfortable.

The interview should take place in a quiet place that allows privacy.

Please introduce each question separately. The interview must flow as a conversation. If you notice that the HCP is hesitant in answering, does not give an in-depth response, or the response is not satisfactory, please probe or ask follow-up questions, but do NOT prompt any specific answer. Several probes are suggested, and you may also ask follow-up questions that are not listed in this guide but are necessary for the complete expression of the HCP's views.

Please **record the interview** using the audio recorder (if HCP consent is provided) and state the ETNA HCP ID number.

All comments from the HCP should be recorded/written verbatim.

Please cross-check the narratives written with the recorded version as a reference, and correct as necessary.

All responses must be kept confidential. Do not discuss or share responses with anyone outside of the ETNA study team.

Script to initiate the interview

"Hello, my name is ___________ and I am a researcher with the ETNA project and we are evaluating monitoring devices for newborns. We want to hear from your experiences and learn from your thoughts and feelings. We will keep what you tell us today confidential, which means that nothing you say will be directly linked to you so please feel free to share. If you feel uncomfortable with any questions, let me know and we will skip it. Before we start, do you have any questions for me? Is it ok to begin? Thank you, I will start the audio-recording now."

R. Demographic information

10. First, we will start with some questions about yourself, what is your age?
11. How many years of education and training have you completed and what is your highest level of education completed? What is your medical background/designation (e.g., doctor, nurse, technician, etc.)?
12. How long have you worked as 'doctor/nurse/technician/etc.?

S. Healthcare provider role

8. How long have you been employed at this healthcare facility?
9. What is your job title and current role here at this facility? How long have you been in this role at this facility?
10. What are your responsibilities in this role?
    Probes: Please share what a typical day as a healthcare provider would be like for you.
11. Are you involved in patient care? If yes, please explain your patient care responsibilities.

T. Facility

For peer review only

10. What are the current constraints to providing care to newborns at this healthcare facility?
    Probes: What makes care more difficult? What would make it easier?
11. How are newborns monitored at this facility? How is this different (if at all) for sick newborns?
12. Does this facility have reliable access to electricity? When was the last electricity outage and how long do they typically last? What happens during power outages at your facility?
    Probes: How do power outages affect patient care? Is there a back-up power supply? If so, what is the process of using the backup power supply and are there any issues around its use (e.g., does it cover all of the equipment needed, any issues in getting permission for its use, fuel prices? Any voltage issues?)
13. Do you have regular access to computers at this facility? If yes, do they work well?
    Probes: Do computers breakdown often? If yes, please describe how the computer breakdowns affect your work as a healthcare provider?
14. Could you describe the technologies that are being used in the delivery and newborn care wards at this facility? What are some concerns you have, or gaps in the technologies available, for maternal and newborn care at this facility?
    Probes: Which healthcare providers use the technologies? What technologies/brands are used? Do healthcare providers use any handheld or portable devices for maternal or newborn care (e.g.,, tablets or Smartphones)? If yes, please describe the technologies and their use.

U. Monitoring devices

1. Are you familiar with the Evaluation of Technologies for Neonates in Africa (ETNA) research study? Are you involved with the study?
   Probes: Are you familiar with the purpose of the study and/or study procedures? Have you previously spoken with any study staff?

We would especially like to learn about your perspectives on continuous monitoring devices.

2. Are continuous monitoring devices used in any capacity at this healthcare facility?
   Probes: If yes, where in the facility? For what purpose? How frequently do you use these devices?
3. Tell me about your experience with continuous monitoring devices. Have you used devices yourself or seen them used?
   Probes: List devices used, then discuss each device sequentially (where, for what purpose?). How frequently have you used these types of devices? Did you find them to be useful? If yes, how so? If no, why not? What sort of training did you receive for the use of these devices?
4. What do you think are some of the benefits (if any) of using continuous monitoring devices? What impacts do you think they could have on routine care at this facility?
5. Do you have any concerns about using continuous monitoring devices? What are the challenges (if any) to using such devices at this facility? Are there any situations you think the use of monitoring devices would not be useful? If so, could you explain with an example?
6. What do you think would be needed to scale up the use of continuous monitoring devices at this facility? What enablers do you think could support this process?
7. Imagine if continuous monitoring devices were scaled up at this facility, how do you think the nurses and doctors that work here would react? How do you think caregivers (mothers, parents, guardians, etc.) would react?

V. EarlySense InSight investigational device

31. Are you familiar with the EarlySense InSight device?
32. Have you ever seen this device before? Have you used it or seen it being used?

Research staff shows EarlySense InSight to HCP and explains how it works.

"The EarlySense InSight is a contact-free physiological monitoring system. The system includes a sensor pad that is placed under the neonate's mattress and can measure pulse, respiratory rate, motion, and sleep status. There is no direct physical contact between the neonate and the sensor pad.

For peer review only

Information from the sensor pad is continuously transmitted to a central display that can be read by hospital staff."

Allow them to touch the InSight device, the mattress pad and the cable that goes between them.

Usability

33. Do you think that healthcare providers in this facility have the skills necessary to use this device? Why or why not?
34. What sort of training do you think would be required for providers in this facility to be able to use this device?
    Probes: What do think the appropriate length of time and method of delivery of training?
35. Which aspects of using this device do you think would be easy to learn? Which aspects would be difficult?
    Probes: What barriers do you anticipate?
36. How do you think using this device would affect providing care to newborns at this facility?
    Probes: Would using this device make provision of care easier or more difficult?
37. What kind of questions do you think caregivers or other hospital staff would have about this device?
38. Are there any changes you would make to this device? If so, what are they?
39. Are there situations where you think this device should not be used? If so, what are they?

Acceptability

40. What do you like (if anything) about this device overall? What do you dislike (if anything)?
    Probes: For example, overall device setup/interface, InSIght device, mattress pad, cable, mobile application, monitor screen/display, etc.?
41. How do you think other healthcare providers, like doctors and nurses, would feel about this device?
42. How do you think caregivers, such as mothers, fathers, mothers-in-law and other family members, would feel about this device?
43. How do you think healthcare administrators and decision-makers at local, district and national levels would react to a recommendation to implement this device at this facility?
    Probes: Discuss at each level (local, district, national) sequentially. What stakeholders would influence the uptake of this technology?
44. Would you consider information collected and displayed by this device trsutworthy? Why or why not?
45. Do you think this device could be useful for monitoring newborns at this facility? Why or why not?
46. Would you like to see your facility incorporate this device into newborn care? Why or why not?

Feasibility

47. Do you think this device is suitable for your facility? What would need to happen in order to integrate this device successfully at this facility? Please explain.
    Probes: For example, staffing availability and skill to use the device, training, complexity of the device, availability of equipment and infrastructure needed for its use, durability and maintenance of device and components, access to spare parts, protocols and guidelines for use, counselling caregivers and informational materials? What could be benefits/drawbacks?
    Probes: For example, ease of use during a patient visit, integration into current flow of hospital operations, acceptance by administrators, etc.?
48. Do you have any other comments about this device that we did not talk about?

W. Sibel ANNE investigational device

1. Are you familiar with the Sibel ANNE device?
2. Have you ever seen this device before? Have you used it/seen it being used?

Research staff show Sibel Advanced Neonatal Epidermal (ANNE) system to HCP and explains how it works:

For peer review only - http://bmjopen.bmj.com/site/about/guidelines.xhtml

"The Sibel Advanced Neonatal Epidermal System, referred to as the ANNE system, uses non-invasive sensors to continuously measure and record a neonate's pulse, respiratory rate, level of oxygen in the blood or SpO2, and skin temperature. One sensor is attached to the neonate's chest and the second sensor is applied to the neonate's foot. Information from the sensors is wirelessly transmitted to a monitor or tablet that can be read by hospital staff."

Allow them to touch the ANNE chest and limb sensors, hydrogel, and tablet display fully.

Usability

3. Do you think that healthcare providers in this facility have the skills necessary to use this device? Why or why not?
4. What sort of training do you think would be required for providers in this facility to be able to use this device?
   Probes: What do think the appropriate length of time and method of delivery of training?
5. Which aspects of using this device do you think would be easy to learn? Which aspects would be difficult?
   Probes: What barriers do you anticipate?
6. How do you think using this device would affect providing care to newborns at this facility?
   Probes: Would using this device make provision of care easier or more difficult?
7. What kind of questions do you think caregivers or other hospital staff would have about this device?
8. Are there any changes you would make to this device? If so, what are they?
9. Are there situations where you think this device should not be used? If so, what are they?

Acceptability

10. What do you like (if anything) about this device overall? What do you dislike (if anything)?
    Probes: For example, overall device setup/interface, ANNE chest and limb sensor, hydrogel, tablet, mobile application, monitor screen/display, etc.?
11. How do you think other healthcare providers, like doctors and nurses, would feel about this device?
12. How do you think caregivers, such as mothers, fathers, mothers-in-law and other family members, would feel about this device?
13. How do you think healthcare administrators and decision-makers at local, district and national levels would react to a recommendation to implement this device at this facility?
    Probes: Discuss at each level (local, district, national) sequentially. What stakeholders would influence the uptake of this technology?
14. Would you trust the information collected and displayed by this device? Why or why not?
15. Do you think this device could be useful for monitoring newborns at this facility? Why or why not?
16. Would you like to see your facility incorporate this device into newborn care? Why or why not?

Feasibility

17. Do you think this device is suitable for your facility? What would need to happen in order to integrate this device successfully at this facility? Please explain.
    Probes: For example, staffing availability and skill to use the device, training, complexity of the device, availability of equipment and infrastructure needed for its use, durability and maintenance of device and components, access to spare parts, protocols and guidelines for use, counselling caregivers and informational materials? What could be benefits/drawbacks?
    Probes: For example, ease of use during a patient visit, integration into current flow of hospital operations, acceptance by administrators, etc.?
18. Do you have any other comments about this device that we did not talk about?

**X. Masimo RAD-97 reference device**
1. Are you familiar with the Masimo RAD-97 device?

For peer review only

2. Have you ever seen this device before? Have you used it/seen it being used?

Research staff shows Masimo Rad-97 device to HCP and explains how it works.

"The Masimo Rad-97 is a non-invasive device that measures a neonate's pulse, respiratory rate, and level of oxygen in the blood or SpO2, in a hospital setting. Information is collected through a skin sensor that is applied to the newborn's hand or foot and a cannula tube that is inserted into the neonate's nostrils. The information is then continuously transmitted to a monitor that can be read by hospital staff."

Allow them to touch the Rad-97, skin sensor and capnography tube fully.

Usability

3. Do you think that healthcare providers in this facility have the skills necessary to use this device? Why or why not?
4. What sort of training do you think would be required for providers in this facility to be able to use this device?
   Probes: What do think the appropriate length of time and method of delivery of training?
5. Which aspects of using this device do you think would be easy to learn? Which aspects would be difficult?
   Probes: What barriers do you anticipate?
6. How do you think using this device would affect providing care to newborns at this facility?
   Probes: Would using this device make provision of care easier or more difficult?
7. What kind of questions do you think caregivers or other hospital staff would have about this device?
8. Are there any changes you would make to this device? If so, what are they?
9. Are there situations where you think this device should not be used? If so, what are they?

Acceptability

10. What do you like (if anything) about this device overall? What do you dislike (if anything)?
    Probes: For example, overall device setup/interface, Rad-97 device, skin sensor, capnography tube, mobile application, monitor screen/display, etc.?
11. How do you think other healthcare providers, like doctors and nurses, would feel about this device?
12. How do you think caregivers, such as mothers, fathers, mothers-in-law and other family members, would feel about this device?
13. How do you think healthcare administrators and decision-makers at local, district and national levels would react to a recommendation to implement this device at this facility?
    Probes: Discuss at each level (local, district, national) sequentially. What stakeholders would influence the uptake of this technology?
14. Would you trust the information collected and displayed by this device? Why or why not?
15. Do you think this device could be useful for monitoring newborns at this facility? Why or why not?
16. Would you like to see your facility incorporate this device into newborn care? Why or why not?

Feasibility

17. Do you think this device is suitable for your facility? What would need to happen in order to integrate this device successfully at this facility? Please explain.
    Probes: For example, staffing availability and skill to use the device, training, complexity of the device, availability of equipment and infrastructure needed for its use, durability and maintenance of device and components, access to spare parts, protocols and guidelines for use, counselling caregivers and informational materials? What could be benefits/drawbacks?
    Probes: For example, ease of use during a patient visit, integration into current flow of hospital operations, acceptance by administrators, etc.?
18. Do you have any other comments about this device that we did not talk about?

H. Closing

For peer review only

11. Taking into consideration the monitoring devices we have talked about today, could you rank the device (if any) you think is the best, second best and third choice in your opinion? Please explain why.<br>12. In terms of feasibility, which device (if any) do you think would be the most appropriate for your healthcare facility and why?<br>13. In terms of acceptability, which device (if any) do you think healthcare providers would like the best and why? Which device (if any) do you think caregivers would prefer and why?<br>14. Do you have any other comments about any of the three devices that we did not talk about?<br>15. Do you have any other comments about newborn monitoring devices or any other comments or concerns overall that we did not get to talk about?
"Thank you for your time and the helpful information you have provided. Your feedback, along with feedback from other people we talk to will be used to recommend solutions for better care."
**Interview end time:** |H|H| : |M|M| *military time*

For peer review only

S3 File Healthcare Provider (HCP) Direct Observation Guide

**A. Administrative information**
**HCP ID number:**
**Date HCP informed consent form (ICF) signed:** |D|D| - |M|M| - |Y|Y|Y|Y|
**HCP ICF signed prior to any observation?** ☐ Yes ☐ No<br>If no, please do not make any observations until the ICF has been completed.
**Name of observer:**
**Neonate ID number:**
**Date of observation:** |D|D| - |M|M| - |Y|Y|Y|Y|
**Location of observation:**<br>☐ **Aga Khan University Hospital – Nairobi**<br>☐ **Pumwani Maternity Hospital**
**Observation start time:** |H|H| : |M|M| *military time*<br>*(Time HCP began device preparation)*
**There are three different phases that can be observed and reported in the fields below:**<br>1. Device preparation and initial application: observing HCP prepare and place device on neonate.<br>2. Ongoing device monitoring and troubleshooting: observing HCP perform regular checks of device placement on neonate (and repositioning if necessary) and data quality, including troubleshooting.<br>3. Device disconnection, removal, and cleaning: observing HCP remove device from neonate, clean and store.<br>**Instructions for qualitative research staff**:<br>Use this document as a guide to conduct observations of one HCP during one or more of the phases described above. Indicate in checklist below which phase(s) were included in this observation session.<br>**Use a new form for each HCP**. Two different HCP should not be included on the same form. **Use a new form for each neonate and for each observation session day**. Two different neonates should not be included on the same form. Two different observation session days should not be included on the same form. Multiple observations of the same neonate by the same HCP on the same day can be included on the same form.

Record observations. All observations must be kept confidential. Do not discuss or share observations with anyone outside of the ETNA study team.

For peer review only

B. Phase(s) observed during this session on the same neonate on the same day (check all that apply)
☐ Device preparation and initial application<br>☐ Ongoing device monitoring and troubleshooting<br>☐ Device disconnection, removal and cleaning

C. Which devices did the HCP use during today's observation?		
EarlySense InSight investigational device	□ Yes	□ No
Sibel ANNE investigational device	□ Yes	□ No
Masimo Rad-97 reference device	□ Yes	□ No

D. PHASE 1: Device initial application
**EarlySense InSight investigational device** *(if device not used, skip to next section)*
Application start time: |H|H| : |M|M| military time<br>Application end time: |H|H| : |M|M| military time<br><br>□ Did not complete device preparation and initial application
Please check those steps that you observed. Comments and observations can be made below.<br><br>***Preparation***<br>☐ Remove neonate from bed/bassinet<br>☐ Place pad under neonate's mattress<br>☐ Gently place neonate back on bed/bassinet with chest above middle of pad<br>☐ Attach pad cord to InSight device<br>☐ Confirm InSight device is seen on EarlySense laptop/CDS<br>***Admission***<br>☐ Correct name of admitting nurse selected in EarlySense laptop/CDS<br>☐ Enter PTID into EarlySense laptop/CDS admit patient screen in MRN (ID) box
Please provide comments if HCP <u>did not complete</u> device preparation and initial application.<br>Also, if HCP was not able to complete steps correctly, what did they do instead?

For peer review only

Did you observe HCP have any challenges or difficulties preparing and/or applying device? What were the problems and how were they resolved?
Did HCP require any assistance when preparing and/or applying device?<br>□ Yes □ No<br>If yes, who assisted HCP?<br>If yes, what kind of assistance was required?
Did you observe any risky situations where mistakes could potentially happen, such as times when HCP almost made a mistake? If yes, please explain.
Did HCP make any other comments to you or their colleagues related to preparing and/or applying device? If yes, record comments verbatim and provide context as necessary.
**Sibel ANNE investigational device** *(if device not used, skip to next section)*
Application start time: |H|H| : |M|M| military time<br>Application end time: |H|H| : |M|M| military time<br>□ Did not complete device preparation and initial application
Please check those steps that you observed. Comments and observations can be made below.<br>***Preparation for data collection***<br>□ ANNE Connect application opened immediately after Sibel iPad unlocked<br>□ Participant ID entered to start data collection session<br>□ Correct chest and limb sensors selected from within ANNE Connect app<br>***Application of ANNE chest sensor***<br>□ Open new hydrogel package and apply hydrogel adhesive to chest sensor or neonate's chest, with gentle but firm pressure<br>□ Place chest sensor on the torso of the neonate and apply gentle but firm pressure to secure sensor to hydrogel adhesive<br>***Application of ANNE limb sensor***<br>□ Insert limb sensor into Velcro strap holes Apply LED to bottom of neonate's foot<br>□ Apply limb sensor on neonate's foot with LED to bottom of neonate's foot Check that photodiode is aligned with LED<br>□ Confirm proper limb sensor placement by checking ANNE Connect application to verify that an error message is not displayed<br>***Confirmation of data collection***<br>□ Correctly close ANNE Connect application (without disconnecting within Connect app)<br>□ Open ANNE Stream application to check quality of vital signs signals
Please provide comments if HCP did not complete device preparation and initial application.<br>Also, if HCP was not able to complete steps correctly and **in order**, what did they do instead?
Did you observe HCP have any challenges or difficulties preparing and/or applying device? What were the problems and how were they resolved?
Did HCP require any assistance when preparing and/or applying device?<br>□ Yes □ No<br>If yes, who assisted HCP?<br>If yes, what kind of assistance was required?
Did you observe any risky situations where mistakes could potentially happen, such as times when HCP almost made a mistake? If yes, please explain.

For peer review only

Did HCP make any other comments to you or their colleagues related to preparing and/or applying device? If yes, record comments verbatim and provide context as necessary.
**Masimo Rad-97 reference device** *(if device not used, skip to next section)*
Application start time: |H|H| : |M|M| military time<br>Application end time: |H|H| : |M|M| military time<br>□ Did not complete device preparation and initial application
Please check those steps that you observed. Comments and observations can be made below.<br>☐ Power on Rad-97 device<br>☐ Plug in a RD Rainbow SET Series Patient Cable to Patient Cable Port on front of Rad-97 device<br>☐ Plug in new, unused NomoLine Infant Cannula to round NomoLine Capnography Input Connector on front of Rad-97 device<br>☐ Attach RD SET Series SpO2 Disposable Sensor to Patient Cable<br>☐ Apply skin sensor to hand or foot<br>☐ Ensure sensor wrapped securely but not too tightly and ensure correct alignment of light and detector<br>☐ Cover sensor to avoid interference from external light sources (as needed)<br>☐ Insert capnography tubing into nostrils, ensuring that the cannula is not obstructed from collecting CO_2<br>☐ Secure cannula in place using neonate-safe adhesive as needed<br>☐ Ensure good quality (square) capnography waveform and high signal quality (perfusion index or PI) on Rad-97 monitor
Please provide comments if HCP <u>did not complete</u> device preparation and initial application.<br>Also, if HCP was not able to complete steps correctly, what did they do instead?
Did you observe HCP have any challenges or difficulties preparing and/or applying device? What were the problems and how were they resolved?
Did HCP require any assistance when preparing and/or applying device?<br>□ Yes □ No<br>If yes, who assisted HCP?<br>If yes, what kind of assistance was required?
Did you observe any risky situations where mistakes could potentially happen, such as times when HCP almost made a mistake? If yes, please explain.
Did HCP make any other comments to you or their colleagues related to preparing and/or applying device? If yes, record comments verbatim and provide context as necessary.
**E. PHASE 2: Ongoing device monitoring and troubleshooting**
**EarlySense InSight investigational device** *(if device not used, skip to next section)*
Did you observe HCP have any challenges or difficulties with device monitoring and/or troubleshooting? What were the problems and how were they resolved?
Did HCP do any troubleshooting during ongoing monitoring? If so, please describe what the issues were, how the HCP addressed them and an estimate for how long it took.

Issue	Solution	Start Time	End Time

For peer review only

Did HCP require any assistance when monitoring the EarlySense InSIght investigational device?<br>□ Yes □ No<br>If yes, who assisted HCP?<br>If yes, what kind of assistance was required?
Did you observe any risky situations where mistakes could potentially happen, such as times when HCP almost made a mistake? If yes, please explain.
Did HCP make any other comments to you or their colleagues related to device monitoring and/or troubleshooting? If yes, record comments verbatim and provide context as necessary.
**Sibel ANNE investigational device** *(if device not used, skip to next section)*
Did HCP complete the following steps correctly? Please check those steps that you observed. Comments and observations can be made below.<br>☐ Open ANNE Stream application to check quality of vital signs waveforms (lines) and perfusion index (PI).<br>☐ Take corrective measures to address signal quality issues (if needed)?
If signal quality issues needed to be addressed, what corrective measures did they take?
Did you observe HCP have any challenges or difficulties with device monitoring and/or troubleshooting? What were the problems and how were they resolved?
Did HCP do any troubleshooting during ongoing monitoring? If so, please describe what the issues were, how the HCP addressed them and an estimate for how long it took.

Issue	Solution	Start Time	End Time

Did HCP require any assistance when monitoring the Sibel ANNE investigational device?<br>□ Yes □ No<br>If yes, who assisted HCP?<br>If yes, what kind of assistance was required?
Did you observe any risky situations where mistakes could potentially happen, such as times when HCP almost made a mistake? If yes, please explain.
Did HCP make any other comments to you or their colleagues related to device monitoring and/or troubleshooting? If yes, record comments verbatim and provide context as necessary.
**Masimo Rad-97 reference device** *(if device not used, skip to next section)*
Did HCP complete the following steps correctly? Please check those steps that you observed. Comments and observations can be made below.<br>☐ Confirm adequate signal quality (PI) for skin sensor<br>☐ Confirm adequate signal quality (waveform) for capnography tube
If signal quality issues needed to be addressed, what corrective measures did they take?<br>☐ Confirm placement of skin sensor<br>☐ Confirm placement of cannula<br>☐ Confirm connection of Patient Cable to Patient Cable port<br>☐ Confirm connection of Capnography Input Connector<br>☐ Other ____

For peer review only

Did you observe HCP have any challenges or difficulties with device monitoring and/or troubleshooting? What were the problems and how were they resolved?
Did HCP do any troubleshooting during ongoing monitoring? If so, please describe what the issues were, how the HCP addressed them and an estimate for how long it took.

Issue	Solution	Start Time	End Time

Did HCP require any assistance when monitoring the Masimo Rad-97 reference device?<br>□ Yes □ No<br>If yes, who assisted HCP?<br>If yes, what kind of assistance was required?
Did you observe any risky situations where mistakes could potentially happen, such as times when HCP almost made a mistake? If yes, please explain.
Did HCP make any other comments to you or their colleagues related to device monitoring and/or troubleshooting? If yes, record comments verbatim and provide context as necessary.
**F. PHASE 3: Device disconnection, removal, and cleaning**
**EarlySense InSight investigational device** *(if device not used, skip to next section)*
Did HCP discharge neonate from EarlySense laptop/CDS correctly? If not, what did HCP do instead?
Did you observe HCP have any challenges or difficulties with device disconnection, removal, and/or cleaning? What were the problems and how were they resolved?
Did the HCP require any assistance with device disconnection, removal, and/or cleaning?<br>□ Yes □ No<br>If yes, who assisted the HCP?<br>If yes, what kind of assistance was required?
Did you observe any risky situations where mistakes could potentially happen, such as times when HCP almost made a mistake? If yes, please explain.
Did HCP make any other comments to you or their colleagues related to device disconnection, removal, and/or cleaning? If yes, record comments verbatim and provide context as necessary.
**Sibel ANNE investigational device** *(if device not used, skip to next section)*
Please check those steps that you observed. Comments and observations can be made below.<br>☐ Disconnect chest and limb sensors from Devices tab of ANNE Stream application<br>☐ Close ANNE Stream application<br>☐ Close ANNE Sync application by swiping up on application<br>☐ Re-open ANNE Connect application<br>☐ Disconnect limb sensor first<br>☐ Disconnect chest sensor<br>☐ End session by selecting "End Session" button from ANNE Connect application<br>☐ Sanitize hands according to study site infection control policy<br>☐ Remove chest sensor by gently pulling off, away from the neonate, on one corner<br>☐ Gently remove any residual adhesive using a saline cleaning wipe<br>☐ Unfasten Velcro button from strap and remove limb sensor

☐ Dispose of used Velcro strap<br>☐ Clean chest and limb sensors, wipe both sides<br>☐ Dispose used cleaning wipe
If HCP was not able to complete steps correctly and in order, what did they do instead?
Did you observe HCP have any challenges or difficulties with device disconnection, removal, and/or cleaning? What were the problems and how were they resolved?
Did the HCP require any assistance with device disconnection, removal, and/or cleaning?<br>□ Yes □ No<br>If yes, who assisted the HCP?<br>If yes, what kind of assistance was required?
Did you observe any risky situations where mistakes could potentially happen, such as times when HCP almost made a mistake? If yes, please explain.
Did HCP make any other comments to you or their colleagues related to device disconnection, removal, and/or cleaning? If yes, record comments verbatim and provide context as necessary.
**Masimo Rad-97 reference device** *(if device not used, skip to next section)*
Did HCP complete the following steps correctly? Please check those steps that you observed. Comments and observations can be made below.<br>☐ Remove adhesive (if present) and capnography tube gently from neonate<br>☐ Carefully remove skin sensor from neonate<br>☐ Dispose of single use capnography tube and disposable skin sensor<br>☐ Unplug capnography tube and patient cable from Rad-97<br>☐ Unplug skin sensor from patient cable<br>☐ Turn off Rad-97
If HCP was not able to complete steps correctly, what did they do instead?
Did you observe HCP have any challenges or difficulties with device disconnection, removal, and/or cleaning? What were the problems and how were they resolved?
Did the HCP require any assistance with device disconnection, removal, and/or cleaning?<br>□ Yes □ No<br>If yes, who assisted the HCP?<br>If yes, what kind of assistance was required?
Did you observe any risky situations where mistakes could potentially happen, such as times when HCP almost made a mistake? If yes, please explain.
Did HCP make any other comments to you or their colleagues related to device disconnection, removal, and/or cleaning? If yes, record comments verbatim and provide context as necessary.

Please note below any further comments that may have not already been covered in above sections. In particular, if you have any observations comparing the HCP's use of the different devices, if applicable.

S4 File Coding tree

Nodes	Sub-nodes	Description
A. Social-demographics information	1. Age	Age of participant
	2. Job title	Job title and current role at the facility
	3. Employment duration at facility	Duration of employment at the healthcare facility
	4. Work experience	Duration worked as a physician, nurse, technician, etc.
	5. Education	Years of education and training completed, highest level of education completed, medical background/designation (e.g., physician, nurse, technician, etc.)
	6. Healthcare provider role	Responsibilities, patient care responsibilities
B. Health system factors	1. Current constraints	Description of the current constraints to providing care to newborns at the healthcare facility. Factors that make care more difficult or easy
	2. Monitoring of newborns at the facility	Methods of newborn monitoring at the facility. How it is different (if at all) for sick newborns
	3. Access to electricity	Description of whether the facility have reliable access to electricity. The last electricity outage and how long do they typically last. What happens during power outages at the facility. How do power outages affect patient care. A back-up power supply. The process of using the backup power supply and any issues around its use (e.g., does it cover all of the equipment needed, any issues in getting permission for its use, fuel prices. Any voltage issues.
	4. Access to computers	Description of whether they have regular access to computers at this facility Whether they work well. Computers breakdown. Ways in which the computer breakdowns affect ones work as a healthcare provider
	5. Technologies used in delivery and newborn unit	Description of the technologies that are being used in the delivery and newborn care wards at this facility. Concerns or gaps in the technologies available, for maternal and newborn care at the facility. Type of healthcare providers who use the technologies. Technologies/ brands used. Whether the healthcare providers use

For peer review only

C. Monitoring devices	1. Familiarity with role and responsibilities with ETNA	Role with the ETNA research study and any ETNA-related responsibilities
	2. Use of continuous monitoring devices	Use of continuous monitoring devices or seen them used. Experience with continuous monitoring devices. List of devices used, how frequently one has used the types of devices. usefulness. Training received for the use of the devices.
	3. Experience with continuous monitoring devices	Description of whether continuous monitoring devices apart from the ETNA devices are used at the healthcare facility. If so, where in the facility, their purpose and frequency of use.
	4. Benefits	Benefits of using continuous monitoring devices and impacts on routine care at the facility
	5. Concerns	Any concerns about using continuous monitoring devices. Challenges to using such devices at this facility. Any situations in which the use of monitoring devices would not be useful.
	6. Need for scale up	What would be needed to scale up the use of continuous monitoring devices at the facility. Enablers that could support the process.
	7. Reaction on use of monitoring devices	Reaction of the nurses and physicians if use of continuous monitoring devices were scaled up at the facility. Reaction of caregivers (mothers, parents, guardians, etc.)
	8. Training	Any mention around training and training needs for monitoring devices in general
D. EarlySense investigational device	A. Familiarity with device	Previous experience with the device
	B. Usability	Discussions around device usability, likes and dislikes about the device, situations where the device should not be used
	C. Acceptability	Feelings of healthcare providers, administrators and caregivers about the device, whether they trusted results and if device should be incorporated
	D. Feasibility	Discussions whether the device would be suitable within their health setting
	A. Familiarity with device	Previous experience with the device

For peer review only

E. Sibel investigational device	B. Usability	Discussions around device usability, likes and dislikes about the device, situations where the device should not be used
	C. Acceptability	Feelings of healthcare providers, administrators and caregivers about the device, whether they trusted results and if device should be incorporated
	D. Feasibility	Discussions whether the device would be suitable within their health setting
F. Masimo RAD-97 reference device	A. Familiarity with device	Previous experience with the device
	B. Usability	Discussions around device usability, likes and dislikes about the device, situations where the device should not be used
	C. Acceptability	Feelings of healthcare providers, administrators and caregivers about the device, whether they trusted results and if device should be incorporated
	D. Feasibility	Discussions whether the device would be suitable within their health setting
G. Closing	A. Rank device	Rank of the device as the best, second best and third choice
	B. Feasibility – most appropriate device	In terms of feasibility, device (if any) that would be the most appropriate device for the healthcare facility
	C. Acceptability – most preferred device	In terms of acceptability, device (if any) that the healthcare providers and caregiver would like the best.
	D. Other comments about the devices	Any other comments about three ETNA study devices in general
	E. Any other comments about newborn monitoring devices or any other comments or concerns	Any other comments about newborn monitoring devices or any other comments or concerns

For peer review only

COREQ Checklist

Consolidated criteria for reporting qualitative studies (COREQ): 32-item checklist

No. Item	Guide questions/description	Reported on Page #
Domain 1: Research team and reflexivity		
Personal Characteristics		
1. Inter viewer/facilitator	Which author/s conducted the interview or focus group?	Pg 6
2. Credentials	What were the researcher's credentials? E.g. PhD, MD	Pg 6
3. Occupation	What was their occupation at the time of the study?	Pg 6
4. Gender	Was the researcher male or female?	Pg 6
5. Experience and training	What experience or training did the researcher have?	Pg 6
Relationship with participants		
6. Relationship established	Was a relationship established prior to study commencement?	Pg 6
7. Participant knowledge of the interviewer	What did the participants know about the researcher? e.g. personal goals, reasons for doing the research	Pg 6
8. Interviewer characteristics	What characteristics were reported about the inter viewer/facilitator? e.g. Bias, assumptions, reasons and interests in the research topic	Pg 6
Domain 2: study design		
Theoretical framework		
9. Methodological orientation and Theory	What methodological orientation was stated to underpin the study? e.g.	Pg 5

For peer review only

	grounded theory, discourse analysis, ethnography, phenomenology, content analysis	
Participant selection		
10. Sampling	How were participants selected? e.g. purposive, convenience, consecutive, snowball	Pg 5
11. Method of approach	How were participants approached? e.g. face-to-face, telephone, mail, email	Pg 6
12. Sample size	How many participants were in the study?	Pg 7
13. Non-participation	How many people refused to participate or dropped out? Reasons?	Pg 7
Setting		
14. Setting of data collection	Where was the data collected? e.g. home, clinic, workplace	Pg 6
15. Presence of non-participants	Was anyone else present besides the participants and researchers?	Pg 6
16. Description of sample	What are the important characteristics of the sample? e.g. demographic data, date	Pg 7
Data collection		
17. Interview guide	Were questions, prompts, guides provided by the authors? Was it pilot tested?	Pg 6; supplementary file 1 and 2
18. Repeat interviews	Were repeat interviews carried out? If yes, how many?	Pg 6
19. Audio/visual recording	Did the research use audio or visual recording to collect the data?	Pg 6
20. Field notes	Were field notes made during and/or after the interview or focus group?	Pg 6
21. Duration	What was the duration of the interviews or focus group?	Pg 6
22. Data saturation	Was data saturation discussed?	Pg 6

For peer review only

23. Transcripts returned	Were transcripts returned to participants for comment and/or correction?	Pg 6
Domain 3: analysis and findings		
Data analysis		
24. Number of data coders	How many data coders coded the data?	Pg 6-7
25. Description of the coding tree	Did authors provide a description of the coding tree?	Pg 7, supplementary file 3
26. Derivation of themes	Were themes identified in advance or derived from the data?	Pg 6-7
27. Software	What software, if applicable, was used to manage the data?	Pg 6-7
28. Participant checking	Did participants provide feedback on the findings?	Pg 6-7
Reporting		
29. Quotations presented	Were participant quotations presented to illustrate the themes/findings? Was each quotation identified? e.g. participant number	Pg 7-14
30. Data and findings consistent	Was there consistency between the data presented and the findings?	Pg 7-14
31. Clarity of major themes	Were major themes clearly presented in the findings?	Pg 7-14
32. Clarity of minor themes	Is there a description of diverse cases or discussion of minor themes?	Pg 7-14

For peer review only
