# Supplementary material for: Qualitative study exploring the feasibility, usability and acceptability of neonatal continuous monitoring technologies at a public tertiary hospital in Nairobi, Kenya
Source: BMJ Open. 2022 Jan 11;12(1):e053486. doi: 10.1136/bmjopen-2021-053486 (PMC8753390; doi:10.1136/bmjopen-2021-053486)
Supplement: Reviewer comments [file bmjopen-2021-053486.reviewer_comments.pdf]

PEER REVIEW HISTORY

BMJ Open publishes all reviews undertaken for accepted manuscripts. Reviewers are asked to complete a checklist review form (**http://bmjopen.bmj.com/site/about/resources/checklist.pdf**) and are provided with free text boxes to elaborate on their assessment. These free text comments are reproduced below.

ARTICLE DETAILS

**TITLE (PROVISIONAL)**	A qualitative study exploring the feasibility, usability, and acceptability of neonatal continuous monitoring technologies at a public tertiary hospital in Nairobi, Kenya
**AUTHORS**	Kinshella, Mai-Lei Woo; Naanyu, Violet; Chomba, Dorothy; Waiyego, Mary; Rigg, Jessica; Coleman, Jesse; Hwang, Bella; Ansermino, J. Mark; Macharia, William; Ginsburg, Amy Sarah

VERSION 1 – REVIEW

**REVIEWER**	Johnson, Ayesha<br>University of South Florida, College of Nursing
**REVIEW RETURNED**	30-Jun-2021

**GENERAL COMMENTS**	1. Please include clarification of why the reference technology was the reference?<br>2. Please also describe in greater detail how the the accuracy, reliability, and performance of novel MCPM technologies in comparison with verified reference was conducted as described on page 6.

**REVIEWER**	Sharpe, Cynthia<br>Starship Children's Health, Neurology
**REVIEW RETURNED**	04-Aug-2021

**GENERAL COMMENTS**	1.Most of the manuscript discusses two investigational technologies, however three systems were studied. It is a little unclear what was and wasnt studied with respect to the third reference Masimo technology. Please clarify.<br>2. Was the earlysense technology trialed in babies sharing a cot during this study?<br>3. Questionnaires show that interviewee's were invited to rank the technologies, but this data is not presented. Is there a reason why?

**REVIEWER**	Egan, Kieren<br>University of Strathclyde, Computer and Information Science
**REVIEW RETURNED**	04-Oct-2021

**GENERAL COMMENTS**	This work was designed to assess the feasibility usability and acceptability of continuous physiological monitoring technologies for neonates in sub-Saharan Africa (EarlySense and Sibel compared to Masimo Rad-97). The approach of the work is on in-depth interviews alongside direct observations. As stated in the conclusions the technologies/systems- there is potential for these systems to deliver equity of access to healthcare services however health system strengthening is required.

	Generally speaking, this is a really well written piece: my main comments are as follows:<br><br>1. References are generally lacking for a paper of this type (13 in total)<br>a. For example there is no reference to the NASSS framework (GreenhalghandAbimbolaTheNASSSFrameworkASynthesisofMultipleTheoriesofTechnologyImplementation.pdf). Although this setting of sub-Saharan Africa will bring its own specific challenges/opportunities, I think it is useful to refer to this given its prominence in the field and the way it captures almost systematically, barriers to implementation/uptake.<br>b. There are also interesting findings here that could be discussed much further, e.g. about caregivers/parents and their acceptance of these technologies- it would be interesting to compare this to other relevant literature.<br><br>2. For both Insight and Sibel, it looks like respondents are asked about how much they think their facility would pay for a device. Given that cost is a key theme stated in the results, would the authors be able to expand further about the feedback they received on this question?<br>a. Further, given maintenance and lack of electricity is an ongoing issue, to what extent can these issues be overcome- i.e. is it feasible that clinicians could truly rely upon the EarlySense and Sibel or would the costs of the [devices]+ [stability of electricity supply]+ [Maintenance] become insurmountable?<br><br>3. The in depth interviews are suggested to take place within 30 to 45 minutes. Was this true in practice? Looking at the in depth interview guide for healthcare providers- (pages 37 to 42) there are something like 50 questions here (possibly more). It would be interesting to know should others be looking to replicate your methodology.<br><br>4. Do the authors have any reflections about how generalizable these findings could be across Sub-Saharan Africa? Are these hospital settings (e.g. resource and infrastructure) typical?<br><br>5. Some further context as a reader would be useful- who are the key decision makers for a setting like the Pumwani Maternity Hospital? What evidence for use/purchasing is generally required should this equipment be used/how are devices purchased?

VERSION 1 – AUTHOR RESPONSE

REVIEWER: 1
Dr. Ayesha Johnson, University of South Florida
Comments to the Author:
1. Please include clarification of why the reference technology was the reference?
Response: Thank you for your comment. We have added the following text under Methods: Study Design and Setting to clarify, “Frequently used in hospitals worldwide, the Masimo Rad-97 reference technology was selected based on its capability for high resolution data collection and neonatal capnometry and pulse oximetry.”
2. Please also describe in greater detail how the the accuracy, reliability, and performance of novel MCPM technologies in comparison with verified reference was conducted as described on page 6.
Response: Thank you for the opportunity to clarify. We have reworked the description to clarify under Methods: Recruitment and Data Collection, “To investigate the accuracy, reliability, and performance of the technologies, IDIs included questions regarding reactions to technology use, consideration of result trustworthiness,

advantages and concerns about using each technology, local health system constraints, and suitability within their facility (Supplementary file 1). … Additionally, direct observations of HCP-D using the technologies covered three different phases of usage for each of the MCPM technologies: 1) technology preparation and initial application; 2) ongoing technology monitoring and troubleshooting; and 3) technology disconnection, removal, and cleaning (Supplementary file 2)."

REVIEWER: 2

Dr. Cynthia Sharpe, Starship Children's Health, University of California San Diego

Comments to the Author:

1. Most of the manuscript discusses two investigational technologies, however three systems were studied. It is a little unclear what was and wasnt studied with respect to the third reference Masimo technology. Please clarify.

Response: Thank you for the opportunity to clarify. We have added that "While the focus of the study is to understand the feasibility, usability, and acceptability of the investigational technologies, the same questions were asked about all three technologies to allow for contextualization and comparison." under Methods: Recruitment and Data Collection.

2. Was the earlysense technology trialed in babies sharing a cot during this study?

Response: In this study, the EarlySense technology was not trials in neonates sharing a cot. According to manufacturer's instructions, the EarlySense technology was to be used with one neonate in each cot. Respondents described this as a challenge due to overcrowding at their facility. As described under the Results section, Feasibility: Numbers of neonates to monitor, a study nurse is quoted in saying that "We've not used [the EarlySense technology] where babies are sharing the baby cot…"

3. Questionnaires show that interviewee's were invited to rank the technologies, but this data is not presented. Is there a reason why?

Reponses: A summary of the participant ranking of the three technologies has now been added to the results. Described under the Results section: Comparison of the investigational and reference technologies, "Of the three technologies, 7 of 10 caregivers rated EarlySense as the most preferable. There was more diversity of responses among health professionals but overall, the Sibel technology was most frequently favorably rated. Seven of 15 HCP who responded to the question rated the Sibel technology as their top choice among the three technologies."

REVIEWER: 3

Dr. Kieren Egan, University of Strathclyde

Comments to the Author:

This work was designed to assess the feasibility usability and acceptability of continuous physiological monitoring technologies for neonates in sub-Saharan Africa (EarlySense and Sibel compared to Masimo Rad-97). The approach of the work is on in-depth interviews alongside direct observations. As stated in the conclusions the technologies/systems- there is potential for these systems to deliver equity of access to healthcare services however health system strengthening is required.

Generally speaking, this is a really well written piece: my main comments are as follows:

1. References are generally lacking for a paper of this type (13 in total)

a. For example there is no reference to the NASSS framework (GreenhalghandAbimbolaTheNASSSFrameworkASynthesisofMultipleTheoriesofTechnologyImplementation.pdf). Although this setting of sub-Saharan Africa will bring its own specific challenges/opportunities, I think it is useful to refer to this given its prominence in the field and the way it captures almost systematically, barriers to implementation/uptake.

b. There are also interesting findings here that could be discussed much further, e.g. about caregivers/parents and their acceptance of these technologies- it would be

interesting to compare this to other relevant literature.
Response: Thank you for your thoughtful review and constructive comments. In response to comment 1, we have added additional references, including two reviews of wearable continuous monitoring sensors for neonates, as well as discussion of our results within the NASSS framework. The following paragraph has been added to the discussion (paragraph 2): "Currently, there are two reviews available of wearable continuous monitoring sensors for neonates, but these only compiled existing products and their key features (12,13). Acceptability and implementation factors were not explored (12,13). The NASSS (non-adoption, abandonment, scale-up, spread, and sustainability) framework posits that increasingly, complexity across seven domains (health condition, technology, value, adopters, organizational capacity, wider system context, and embedding/adaption over time) contributes to the non-adoption of novel health technologies (14). Addressing the first three domains, MCPM technologies are standard in the care of vulnerable neonates in high-resource health settings and study participants in our low-resource health setting valued their importance for improving quality of care and expressed appreciation for user-friendly design features. However, acceptability and systemic factors within their organizational and infrastructural context emerged as critical domains impacting capacity for scale-up, spread, and sustainability. Our study helps to fill the current gap in understanding these domains for MCPM technologies for neonates in resource-limited settings where they are not yet routinely implemented."
Overall, additional references added to the manuscript include:
• Zhu Z, Liu T, Li G, Li T, Inoue Y. Wearable Sensor Systems for Infants. Sensors. 2015 Feb 5;15(2):3721–49.
• Memon SF, Memon M, Bhatti S. Wearable technology for infant health monitoring: A survey. IET Circuits, Devices Syst. 2020;14(2):115–29.
• Greenhalgh T, Abimbola S. The NASSS Framework – A Synthesis of Multiple Theories of Technology Implementation. Stud Health Technol Inform. 2019;263:193–204.
• Kinshella MLW, Walker CR, Hiwa T, Vidler M, Nyondo-Mipando AL, Dube Q, et al. Barriers and facilitators to implementing bubble CPAP to improve neonatal health in sub-Saharan Africa: A systematic review. Public Health Rev. 2020 Apr 28;41(1):6.
• Leonard E, de Kock I, Bam W. Barriers and facilitators to implementing evidence-based health innovations in low- and middle-income countries: A systematic literature review. Eval Program Plann. 2020 Oct 1;82:101832.
2. For both Insight and Sibel, it looks like respondents are asked about how much they think their facility would pay for a device. Given that cost is a key theme stated in the results, would the authors be able to expand further about the feedback they received on this question?
a. Further, given maintenance and lack of electricity is an ongoing issue, to what extent can these issues be overcome- i.e. is it feasible that clinicians could truly rely upon the EarlySense and Sibel or would the costs of the [devices]+ [stability of electricity supply]+ [Maintenance] become insurmountable?
Response: Thank you for raising the important topic of cost. Because these are investigational technologies, we do not have good estimates of the final costs to provide accurate ranges and three of five healthcare administrators elected to not respond to the question on at least one of the technologies. While administrators hesitated to speculate on an amount their facility would pay, they did talk about the issue of costs more generally including both initial costs to obtain the technology but also maintenance costs, which are included in the results.
We agree with Dr Egan that maintenance and lack of electricity are critical issues that require addressing for effective and sustainable implementation. We highlight in the discussion and conclusion that "technology on its own cannot overcome feasibility

challenges of basic infrastructural gaps" and that "Innovative MCPM technologies have the potential to significantly improve neonatal care in sub-Saharan African healthcare facilities, but health system strengthening is also critical to support their sustainable uptake into routine care."

3. The in depth interviews are suggested to take place within 30 to 45 minutes. Was this true in practice? Looking at the in depth interview guide for healthcare providers- (pages 37 to 42) there are something like 50 questions here (possibly more). It would be interesting to know should others be looking to replicate your methodology.

Response: Good point. We clarified with our colleague conducting the interviews and she reported that interviews took between 18 to 78 minutes to conduct with an average length of 46.6 minutes. This has been updated in the manuscript under the Methods: Recruitment and data collection section. Shorter interviews were among caregivers who often gave more brief responses and the longest interviews were with study nurses (HCP-D). Due to the semi-structured format of the interviews, some questions were skipped if they were already answered in a previous question.

4. Do the authors have any reflections about how generalizable these findings could be across Sub-Saharan Africa? Are these hospital settings (e.g. resource and infrastructure) typical?

Response: We added an extra line to reflect on the generalizability of findings across hospitals in sub-Saharan Africa. Added to the discussion, "The experience at PMH may be reflective of feasibility constraints in other large public hospitals in sub-Saharan Africa where adequate human, equipment, and infrastructural resources have been identified as limiting factors in the implementation of newborn health innovations (16,17)."

5. Some further context as a reader would be useful- who are the key decision makers for a setting like the Pumwani Maternity Hospital? What evidence for use/purchasing is generally required should this equipment be used/how are devices purchased?

Response: We have provided additional contextual information on the procurement process at Pumwani Maternity Hospital. Added further elaboration to the Feasibility: Cost and Maintenance subsection under results: "As a public hospital, HCA shared that PMH followed the government procurement process, and while there were a procurement and budget committee and a health management board at PMH that took into account what HCP needed in their department, the medical superintendent had to approve the purchase and the Kenya Medical Supplies Authority (KEMSA) did most of the purchasing. Consequently, HCA said that a lack of funds at PMH to purchase equipment is a challenge. HCA shared that PMH was often reliant on donors and partners to fill the gaps, "not having funds for the equipment is a big issue because money from the county or NMS (Nairobi Metropolitan Services) is not available to us, and we have to look for donors and partners who are able to procure the equipment for us" (HCA, 4)."

Again, we are grateful for the opportunity to respond to these comments and to resubmit our revised manuscript. Please do not hesitate to contact us should you have any questions or areas that need further clarification.

Best regards,

Mai-Lei Woo Kinshella and Amy Sarah Ginsburg, on behalf of the authors

VERSION 2 – REVIEW

**REVIEWER**	Sharpe, Cynthia<br>Starship Children's Health, Neurology
**REVIEW RETURNED**	28-Nov-2021

**GENERAL COMMENTS**	Excellent study of a vitally important research issue.
